# Supplementary material for: Medication adherence and its associated factors among oral pre-exposure prophylaxis (PrEP) users in China: The Real-world E-consumer Cohort of PrEP study
Source: PLoS Med. 2026 Feb 26;23(2):e1004733. doi: 10.1371/journal.pmed.1004733 (PMC12944781; doi:10.1371/journal.pmed.1004733)
Supplement: S1 Fig — The bar chart presents the distribution of self-reported reasons for not adhering to PrEP among ED users at baseline and 1-, 3-, and 6-month follow-ups. (PPTX) [file pmed.1004733.s001.pptx]

## Slide 1
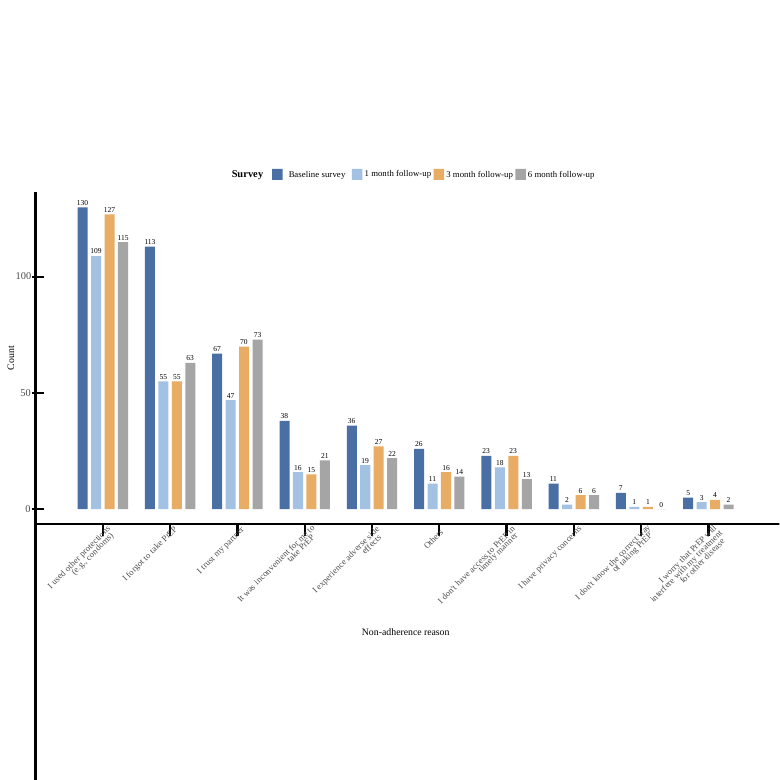

1 month follow-up
Survey
3 month follow-up
6 month follow-up
Baseline survey
130
127
115
113
109
100
73
70
67
Count
63
55
55
50
47
38
36
27
26
23
23
22
21
19
18
16
16
15
14
13
11
11
7
6
6
5
4
3
2
2
1
1
0
0
Others
effects
take PrEP
I trust my partner
timely manner
of taking PrEP
I forgot to take PrEP
(e.g., condoms)
I worry that PrEP will
I used other protections
I have privacy concerns
I experience adverse side
for other disease
I don't know the correct way
It was inconvenient for me to
I don't have access to PrEP in
interfere with my treatment
Non-adherence reason
