# Supplementary material for: Medication adherence and its associated factors among oral pre-exposure prophylaxis (PrEP) users in China: The Real-world E-consumer Cohort of PrEP study
Source: PLoS Med. 2026 Feb 26;23(2):e1004733. doi: 10.1371/journal.pmed.1004733 (PMC12944781; doi:10.1371/journal.pmed.1004733)
Supplement: S2 Fig — The bar chart presents the distribution of self-reported reasons for not adhering to PrEP among daily users at baseline and 1-, 3-, and 6-month follow-ups. (PPTX) [file pmed.1004733.s002.pptx]

## Slide 1
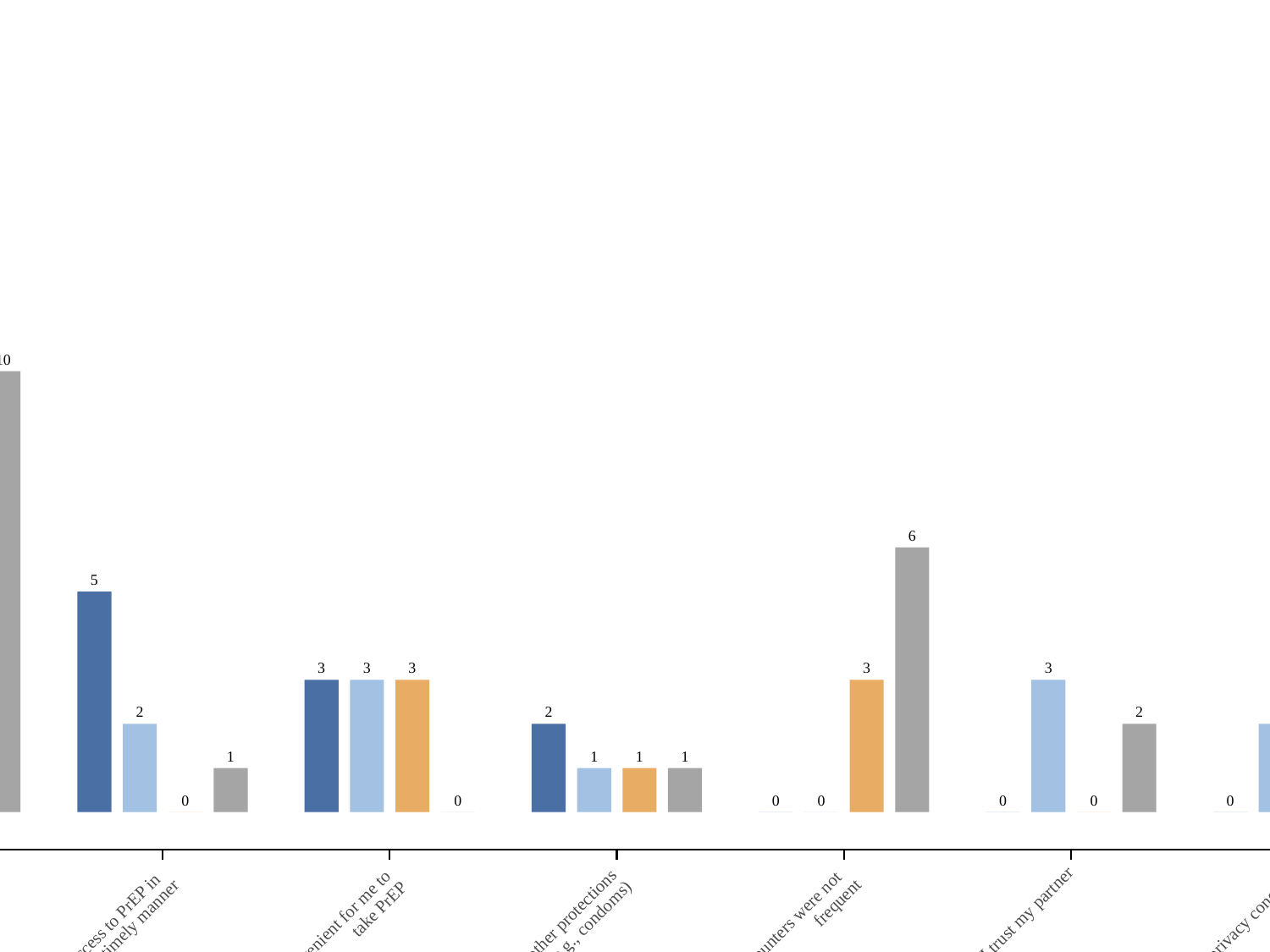

Survey
1 month fllow-up
3 month fllow-up
6 month fllow-up
Baseline survey
17
15
13
12
10
10
Count
6
5
5
3
3
3
3
3
2
2
2
2
2
1
1
1
1
0
0
0
0
0
0
0
0
0
0
0
0
0
Others
frequent
take PrEP
I trust my partner
timely manner
I forgot to take PrEP
(e.g., condoms)
I used other protections
I have privacy concerns
It was inconvenient for me to
I don't have access to PrEP in
My sexual encounters were not
Non-adherence reason
