# Supplementary material for: Medication adherence and its associated factors among oral pre-exposure prophylaxis (PrEP) users in China: The Real-world E-consumer Cohort of PrEP study
Source: PLoS Med. 2026 Feb 26;23(2):e1004733. doi: 10.1371/journal.pmed.1004733 (PMC12944781; doi:10.1371/journal.pmed.1004733)
Supplement: S1 Table — This table presents the patterns of missing survey responses across study timepoints, showing the number and proportion of participants who missed one, two, or three surveys out of the total cohort, thereby describing participant retention in the longitudinal study. (DOCX) [file pmed.1004733.s003.docx]

**S1 Table****.** Patterns of missing survey responses

| **Patterns** | **n** | **% (n/657)** |
| --- | --- | --- |
| Missing survey responses at three waves | 47 | 7.1% |
| Missing survey responses at two waves | 56 | 8.5% |
| Missing survey responses at one wave | 78 | 11.9% |
| Total | 181 | 27.5% |
