# Supplementary material for: Medication adherence and its associated factors among oral pre-exposure prophylaxis (PrEP) users in China: The Real-world E-consumer Cohort of PrEP study
Source: PLoS Med. 2026 Feb 26;23(2):e1004733. doi: 10.1371/journal.pmed.1004733 (PMC12944781; doi:10.1371/journal.pmed.1004733)
Supplement: S2 Table — This table summarizes the distribution of demographic, sexual behavior, PrEP-related, and psychosocial variables among participants who had not initiated PrEP across four time points (baseline and 1-, 3-, and 6-month follow-ups), illustrating changes or stability in participant characteristics over the follow-up period. (DOCX) [file pmed.1004733.s004.docx]

**S2 Table.** Distribution of sexual behaviors, PrEP-related variables, and psychosocial variables among participants who have not initiated PrEP

| **Variables** | **Baseline**  **(n=36)** | **1 month**  **(n=22)** | **3 month**  **(n=13)** | **6 month**  **(n=10)** |
| --- | --- | --- | --- | --- |
| **Age (years, Mean±SD)** | 28.7±6.1 | 29.4±6.2 | 29.1±6.5 | 28.8±5.6 |
| 18-29 | 22 (61.1) | 12 (54.5) | 7 (53.8) | 5 (50.0) |
| 30-39 | 11 (30.6) | 8 (36.4) | 5 (38.5) | 5 (50.0) |
| >40 | 3 (8.3) | 2 (9.1) | 1 (7.7) | 0 (0.0) |
| **Designated Sex at Birth** |  |  |  |  |
| Male | 36 (100.0) | 22 (100.0) | 13 (100.0) | 10 (100.0) |
| Female | 0 (0.0) | 0 (0.0) | 0 (0.0) | 0 (0.0) |
| **Ethnicity** |  |  |  |  |
| Han | 33 (91.7) | 21 (95.5) | 11 (84.6) | 10 (100.0) |
| Others | 3 (8.3) | 1 (4.5) | 2 (15.4) | 0 (0.0) |
| **Employment** |  |  |  |  |
| Students/Unemployed | 7 (19.4) | 4 (18.2) | 3 (23.1) | 2 (20.0) |
| Employed | 29 (80.6) | 18 (81.8) | 10 (76.9) | 8 (80.0) |
| **Education Level** |  |  |  |  |
| Junior high school or below | 0 (0.0) | 0 (0.0) | 0 (0.0) | 0 (0.0) |
| Senior high school / Vocational high school / Technical secondary school | 0 (0.0) | 0 (0.0) | 0 (0.0) | 0 (0.0) |
| College diploma (Associate degree) / Bachelor’s degree | 29 (80.6) | 17 (77.3) | 10 (76.9) | 7 (70.0) |
| Master’s degree or above | 7 (19.4) | 5 (22.7) | 3 (23.1) | 3 (30.0) |
| **Marital Status** |  |  |  |  |
| Married or living with a partner | 2 (5.6) | 3 (13.6) | 0 (0.0) | 0 (0.0) |
| Unmarried/Divorced/Separated/Widowed | 34 (94.4) | 19 (86.4) | 13 (100.0) | 10 (100.0) |
| **Monthly income (CNY) ^a^** |  |  |  |  |
| ≤3000 | 6 (16.7) | 5 (22.7) | 3 (23.1) | 2 (20.0) |
| 3001-5000 | 5 (13.9) | 1 (4.5) | 0 (0.0) | 0 (0.0) |
| 5001-7000 | 3 (8.3) | 2 (9.1) | 0 (0.0) | 0 (0.0) |
| 7001-10000 | 8 (22.2) | 7 (31.8) | 5 (38.5) | 4 (40.0) |
| 10001-15000 | 7 (19.4) | 2 (9.1) | 2 (15.4) | 1 (10.0) |
| 15001-20000 | 4 (11.1) | 2 (9.1) | 2 (15.4) | 2 (20.0) |
| >20000 | 3 (8.3) | 3 (13.6) | 1 (7.7) | 1 (10.0) |
| **Sexually active** |  |  |  |  |
| Yes | 32 (88.9) | 15 (68.2) | 10 (76.9) | 8 (80.0) |
| No | 4 (11.1) | 7 (31.8) | 3 (23.1) | 2 (20.0) |
| **Chemsex** |  |  |  |  |
| Yes | 10 (31.1) | 4 (26.7) | 1 (10.0) | 3 (37.5) |
| No | 22 (68.8) | 11 (73.3) | 9 (90.0) | 5 (62.5) |
| **Condom use** |  |  |  |  |
| Inconsistent | 8 (25.0) | 4 (26.7) | 1 (10.0) | 3 (37.5) |
| Consistent | 24 (75.0) | 11 (73.3) | 9 (90.0) | 5 (62.5) |
| **Sexual partner** |  |  |  |  |
| 1 | 9 (28.1) | 6 (40.0) | 3 (30.0) | 2 (25.0) |
| >1 | 23 (71.9) | 9 (60.0) | 7 (70.0) | 6 (75.0) |
| **Sexual role** |  |  |  |  |
| Receptive or versatile | 11 (34.4) | 4 (26.7) | 2 (20.0) | 0 (0.0) |
| Insertive | 21 (65.6) | 11 (73.3) | 8 (80.0) | 8 (100.0) |
| **Commercial sex** |  |  |  |  |
| Yes | 2 (6.2) | 0 (0.0) | 1 (10.0) | 2 (25.0) |
| No | 30 (93.8) | 15 (100.0) | 9 (90.0) | 6 (75.0) |
| **PrEP knowledge** |  |  |  |  |
| Correct | 23 (63.9) | 11 (50.0) | 8 (61.5) | 6 (60.0) |
| In correct | 13 (36.1) | 11 (50.0) | 5 (38.5) | 4 (40.0) |
| **Self-efficacy of PrEP adherence** | 4.3±1.8 | 4.1±2.1 | 4.7±1.9 | 4.6±2.0 |
| **PrEP-related stigma** | 27.8±6.7 | - | 24.6±7.0 | 26.9±6.8 |
| **Depressive symptoms** | 7.9±5.5 | - | 8.0±2.6 | 10.3±7.4 |
| No | 11 (30.6) | - | 1 (7.7) | 3 (30.0) |
| Mild to severe | 25 (69.4) | - | 12 (92.3) | 7 (70.0) |
| **Resilience** | 4.5±1.7 | - | 5.3±1.4 | 6.3±2.3 |

PrEP, Pre-exposure prophylaxis; SD, Standard deviation; ED, event-driven; CNY, Chinese Yuan.

a 1 CNY=0.14 USD
