# Supplementary material for: Medication adherence and its associated factors among oral pre-exposure prophylaxis (PrEP) users in China: The Real-world E-consumer Cohort of PrEP study
Source: PLoS Med. 2026 Feb 26;23(2):e1004733. doi: 10.1371/journal.pmed.1004733 (PMC12944781; doi:10.1371/journal.pmed.1004733)
Supplement: S3 Table — The table presents the proportions of non-adherent ED users who missed the correct dose at each critical dosing time point of the ‘2-1-1’ dosing schedule during the past month, across four survey waves. (DOCX) [file pmed.1004733.s005.docx]

**S3 Table.** Patterns of missing doses among non-adherent event-driven (ED) PrEP users in the past month at each survey wave.

| **Non-adherence to ‘2-1-1’ Dosing Schedule ^a^** | **Baseline**  **(n=219) ^b^** | **1-month follow-up**  **(n=193) ^b^** | **3-month follow-up**  **(n=168) ^b^** | **6-month follow-up**  **(n=144)^b^** |
| --- | --- | --- | --- | --- |
| Missed correct dose at dosing time point A | 77 (35.2) | 91 (47.2) | 89 (53.0) | 85 (59.0) |
| Missed correct dose at dosing time point B | 85 (38.9) | 96 (49.7) | 93 (55.4) | 91 (63.2) |
| Missed correct dose at dosing time point C | 100 (45.7) | 111 (57.5) | 104 (61.9) | 93 (64.6) |

a: Dosing time point A refers to the double dose taken 2–24 hours before sex, dosing time point B refers to the first single dose taken 24 hours the first dose, and dosing time point C refers to the second single dose taken 48 hours after the first dose.

b: The denominator for each percentage includes only those ED users at each follow-up who reported suboptimal adherence in the past month.
