# Supplementary material for: Medication adherence and its associated factors among oral pre-exposure prophylaxis (PrEP) users in China: The Real-world E-consumer Cohort of PrEP study
Source: PLoS Med. 2026 Feb 26;23(2):e1004733. doi: 10.1371/journal.pmed.1004733 (PMC12944781; doi:10.1371/journal.pmed.1004733)
Supplement: S4 Table — This table presents PrEP adherence across four study timepoints among users of daily and event-driven (ED) regimens. It reports the number and proportion of participants achieving optimal adherence, as well as the mean adherence rate (mean ± SD) for the daily regimen, illustrating adherence patterns and consistency over time. (DOCX) [file pmed.1004733.s006.docx]

**S4 Table.** Adherence among PrEP users across four study timepoints

| PrEP regimen | Visit | N | Optimal adherence n (%)^a^ | Adherence rate  (%; mean±SD)^b^ |
| --- | --- | --- | --- | --- |
| Daily | Baseline | 92 | 77 (83.7) | 98.7 ± 4.3 |
|  | 1 month | 102 | 83 (81.4) | 97.8 ± 10.2 |
|  | 3 month | 98 | 86 (87.8) | 99.3 ± 2.4 |
|  | 6 month | 89 | 75 (84.3) | 97.1 ± 13.1 |
| ED (past one month) | Baseline | 273 | 154 (41.3) | N/A |
|  | 1 month | 339 | 146 (43.1) | N/A |
|  | 3 month | 307 | 139 (45.3) | N/A |
|  | 6 month | 304 | 160 (52.6) | N/A |
| ED (past three month) | Baseline | 458 | 177 (38.6) | N/A |
|  | 3 month | 377 | 163 (43.3) | N/A |
|  | 6 month | 374 | 165 (44.1) | N/A |

PrEP: Pre-exposure Prophylaxis; ED, event-driven

a: “Optimal adherence” was defined as taking all prescribed doses without any missed doses in the past month for participants on the daily PrEP regimen, and as strict compliance with the recommended ‘2-1-1’ dosing schedule (two pills 2–24 hours before sex, one pill 24 hours after, and one pill 48 hours after) for those on the ED PrEP regimen.

b: Adherence rate (%) = (30-missed doses)/30 × 100.
