# Supplementary material for: Medication adherence and its associated factors among oral pre-exposure prophylaxis (PrEP) users in China: The Real-world E-consumer Cohort of PrEP study
Source: PLoS Med. 2026 Feb 26;23(2):e1004733. doi: 10.1371/journal.pmed.1004733 (PMC12944781; doi:10.1371/journal.pmed.1004733)
Supplement: S5 Table — The table presents univariate and multivariable logistic regression results (Model 2) assessing factors associated with optimal PrEP adherence in the past one month among ED users across four survey waves. Odds ratios (OR), adjusted odds ratios (aOR), and corresponding 95% confidence intervals (CI) are reported. (DOCX) [file pmed.1004733.s007.docx]

**S5 Table.** Factors associated with optimal medication adherence in the past month across four surveys among ED users.

| **Variables** | **Model 2: Optimal adherence in the past one month** | | | |
| --- | --- | --- | --- | --- |
|  | **OR** | **95% CI** | **aOR** | **95% CI** |
| Age | 1·00 | (0·96, 1·04) | 1·01 | (0·97, 1·05) |
| Ethnicity (Others vs. Han) | 1·31 | (0·95, 1·82) | 0·54 | (0·21, 1·38) |
| Monthly income (>3000 CNY vs. ≤3000 CNY) | 1·21 | (0·75, 1·94) | 0·97 | (0·50, 1·89) |
| Marital status (Unmarried/Divorced/Separated/Widowed vs. Married/Living with a Partner) | 1·10 | (0·65, 1·86) | 0·62 | (0·31, 1·23) |
| Education (College and above vs. High school or below) | 0·67*** | (0·66, 0·67) | 0·56 | (0·23, 1·36) |
| Employment status (Employed vs. Students/Unemployed) | 1·00 | (0·53, 1·89) | 0·80 | (0·36, 1·81) |
| Knowledge of event-driven regimen (Correct vs. Incorrect) | 1·73*** | (1·72, 1·73) | 1·64 | (0·99, 2·71) |
| Having multiple homosexual partners in the past three months (Multiple vs. One same-sex partne) | 0·55** | (0·36, 0·84) | 0·55* | (0·33, 0·92) |
| Sexual role in the past three months (Receptive or versatile vs. Insertive) | 1·13 | (0·76, 1·69) | 1·18 | (0·76, 1·83) |
| Chemsex in the past three months (Having chemsex vs. No chemsex) | 0·64*** | (0·64, 0·64) | 0·80 | (0·51, 1·25) |
| Condom use in the past three months (Inconsistent vs. Consistent use) | 1·26 | (0·87, 1·82) | 1·44 | (0·92, 2·25) |
| Commercial sex in the past three months (Having commercial sex vs. No commercial sex) | 0·83 | (0·43, 1·59) | 0·99 | (0·46, 2·15) |
| Self-efficacy of being adhere to PrEP | 1·69*** | (1·52, 1·88) | 1·58*** | (1·38, 1·82) |
| Resilience | 1·04 | (0·95, 1·19) | 0·99 | (0·88, 1·12) |
| Depressive symptoms | 0·98 | (0·94, 1·01) | 0·96 | (0·92, 1·01) |
| PrEP-related stigma | 0·97*** | (0·96, 0·97) | 0·98 | (0·95, 1·01) |

ED, event-driven; PrEP, Pre-exposure prophylaxis; CNY, Chinese Yuan; OR, odds ratio; CI, confidence interval; aOR, adjusted odds ratio
