# Supplementary material for: Medication adherence and its associated factors among oral pre-exposure prophylaxis (PrEP) users in China: The Real-world E-consumer Cohort of PrEP study
Source: PLoS Med. 2026 Feb 26;23(2):e1004733. doi: 10.1371/journal.pmed.1004733 (PMC12944781; doi:10.1371/journal.pmed.1004733)
Supplement: S6 Table — This table presents univariate and multivariable logistic regression results (Model 3) examining factors associated with consistent adherence across the 3- and 6-month follow-ups. Odds ratios (OR), adjusted odds ratios (aOR), and their corresponding 95% confidence intervals (CI) are reported. (DOCX) [file pmed.1004733.s008.docx]

**S6 Table.** Factors associated with consistent medication adherence during 3- and 6-month follow-ups among ED users.

| **Variables** | **Model 3: Consistent adherence during the follow-ups** ^d^ | | | |
| --- | --- | --- | --- | --- |
|  | **OR** | **95% CI** | **aOR** | **95% CI** |
| Age | 1·04* | (1·01, 1·08) | 1·06** | (1·02, 1·11) |
| Ethnicity (Others vs. Han) | 1·26 | (0·45, 3·30) | 0·91 | (0·29, 2·81) |
| Monthly income (>3000 CNY vs. ≤3000 CNY) | 1·27 | (0·79, 2·07) | 1·14 | (0·59, 2·23) |
| Marital status (Unmarried/Divorced/Separated/Widowed vs. Married/Living with a Partner) | 0·91 | (0·49, 1·65) | 0·54 | (0·25, 1·11) |
| Education (College and above vs. High school or below) | 0·49 | (0·17, 1·48) | 0·99 | (0·24, 3·95) |
| Employment status (Employed vs. Students/Unemployed) | 1·40 | (0·78, 2·61) | 1·38 | (0·60, 3·19) |
| Knowledge of event-driven regimen ^a^ (Correct vs. Incorrect) | 1·01 | (0·59, 1·75) | 1·10 | (0·58, 2·13) |
| Having multiple same-sex partners ^b^ (Yes vs. No) | 0·51* | (0·28, 0·93) | 0·53 | (0·26, 1·07) |
| Sexual role ^b^ (Receptive or versatile vs. Insertive) | 1·22 | (0·76, 1·96) | 1·05 | (0·60, 1·85) |
| Having chemsex ^b^ (Yes vs. No) | 0·47** | (0·29, 0·76) | 0·42* | (0·23, 0·74) |
| Having inconsist condom use ^b^ (Yes vs. No) | 0·83 | (0·52, 1·33) | 0·94 | (0·54, 1·64) |
| Having commercial sex ^b^ (Yes vs. No) | 0·57 | (0·23, 1·25) | 0·59 | (0·22, 1·46) |
| Self-efficacy of being adhere to PrEP ^c^ | 1·85*** | (1·52, 2·28) | 1·91*** | (1·55, 2·41) |
| Resilience ^c^ | 1·29* | (1·06, 1·58) | 1·16 | (0·90, 1·51) |
| Depressive symptoms ^c^ | 0·96 | (0·90, 1·02) | 0·98 | (0·90, 1·06) |
| PrEP-related stigma ^c^ | 0·96 | (0·91, 1·01) | 0·99 | (0·93, 1·05) |

ED, event-driven; PrEP, Pre-exposure prophylaxis; CNY, Chinese Yuan; OR, odds ratio; CI, confidence interval; aOR, adjusted odds ratio

a: PrEP knowledge was recoded as “correct” if participants answered correctly in both follow-ups.

b: Multiple same-sex partners, sexual role, chemsex, consistent condom use, and commercial sex were classified as “yes” if participants reported engaging in these behaviors at least once across the two follow-up assessments.

c: Self-efficacy for being adhere to PrEP, resilience, depressive symptoms, and PrEP-related stigma were assessed using the mean scores from the two follow-up assessments.

d: Consistent medication adherence was defined as reporting adherence in the past 3 months at both the 3-month and 6-month follow-ups.
