# Supplementary material for: Medication adherence and its associated factors among oral pre-exposure prophylaxis (PrEP) users in China: The Real-world E-consumer Cohort of PrEP study
Source: PLoS Med. 2026 Feb 26;23(2):e1004733. doi: 10.1371/journal.pmed.1004733 (PMC12944781; doi:10.1371/journal.pmed.1004733)
Supplement: S7 Table — This table presents the results of univariable linear mixed-effects regression analyses examining factors associated with adherence rate (%) among daily PrEP users. It reports regression coefficients (β), 95% confidence intervals (CI), and P-values for demographic, behavioral, and psychosocial variables to identify potential predictors of adherence. (DOCX) [file pmed.1004733.s009.docx]

**S7 Table.** Univariable linear mixed-effects regression results for adherence rate (%) among daily PrEP users

| **Variables** | **Daily users** | | |
| --- | --- | --- | --- |
|  | ***β*** | **95% CI** | ***P*-value** |
| Age | -0.03 | (-0.22, 0.16) | 0.753 |
| Ethnicity (Others vs. Han) | 0.94 | (-2.47, 4.34) | 0.586 |
| Monthly income (>3000 CNY vs. ≤3000 CNY) | -0.67 | (-3.70, 2.37) | 0.665 |
| Marital status (Unmarried/Divorced/Separated/Widowed vs. Married/Living with a Partner) | -3.51 | (-6.30, -0.72) | **0.014** |
| Education (College and above vs. High school or below) | -1.43 | (-5.08, 2.21) | 0.437 |
| Employment status (Employed vs. Students/Unemployed) | 2.87 | (-2.31, 8.06) | 0.278 |
| Knowledge of event-driven regimen (Correct vs. Incorrect) | -0.56 | (-2.28, 1.16) | 0.525 |
| Having multiple homosexual partners in the past three months (Multiple vs. One same-sex partner) | 1.82 | (-0.36, 3.99) | 0.102 |
| Sexual role in the past three months (Receptive or versatile vs. Insertive) | 0.38 | (-1.67, 2.43) | 0.718 |
| Chemsex in the past three months (Having chemsex vs. No chemsex) | 0.37 | (-1.54, 2.28) | 0.705 |
| Condom use in the past three months (Inconsistent vs. Consistent use) | 0.20 | (-1.76, 2.16) | 0.839 |
| Commercial sex in the past three months (Having commercial sex vs. No commercial sex) | 0.77 | (-1.95, 3.50) | 0.578 |
| Self-efficacy of being adhere to PrEP | 0.64 | (0.05, 1.23) | **0.034** |
| Resilience | -0.19 | (-0.70, 0.32) | 0.457 |
| Depressive symptoms | -0.01 | (-0.17, 0.17) | 0.978 |
| PrEP-related stigma | 0.01 | (-0.12, 0.14) | 0.866 |

PrEP, Pre-exposure prophylaxis; CNY, Chinese Yuan; CI, confidence interval
