# Supplementary material for: Medication adherence and its associated factors among oral pre-exposure prophylaxis (PrEP) users in China: The Real-world E-consumer Cohort of PrEP study
Source: PLoS Med. 2026 Feb 26;23(2):e1004733. doi: 10.1371/journal.pmed.1004733 (PMC12944781; doi:10.1371/journal.pmed.1004733)
Supplement: S1 File — This file provides details of the study design, data collection procedures, outcome definitions, and prespecified analytical methods. (DOCX) [file pmed.1004733.s010.docx]

**Study Protocol for the Real-world E-Consumer Cohort of pre-exposure prophylaxis (RECOPE) in China**

1. **Rationale of the study**

HIV/AIDS remains a major public health challenge in China and worldwide. According to the Joint United Nations Programme on HIV/AIDS (UNAIDS), 39.9 million people are living with HIV globally in 2023, with approximately 1.5 million new infections reported that year [1]. In China, over 1.2 million individuals were living with HIV by the end of 2022, and more than 107,000 new infections were reported in the same year [2]. The epidemic continues to disproportionately affect vulnerable populations such as men who have sex with men (MSM) and sex workers.

Pre-exposure prophylaxis (PrEP) is a proven biomedical prevention tool that reduces the risk of HIV acquisition through sexual contact by up to 99% when taken correctly [3]. Two main PrEP regimens are available: (1) the daily regimen, recommended for all high-risk populations, which involves taking one pill daily; and (2) the event-driven (ED) regimen, also known as “2-1-1,” recommended for MSM, which involves 2 pills 2–24 hours before sex, followed by 1 pill 24 hours and 48 hours later. The World Health Organization and Chinese Expert Consensus both strongly recommend PrEP for individuals at high risk of HIV[4,5]. PrEP is officially approved in China in 2020, marking an important step toward HIV prevention scale-up.

With the rapid development of internet hospitals and online health platforms, new pathways for PrEP access have emerged. Online platforms offer discreet and convenient services, reducing barriers related to stigma and healthcare access. For MSM and other key populations, online purchase of PrEP may overcome barriers encountered in traditional clinic settings. However, unlike face-to-face clinical services, online platforms may lack structured mechanisms for adherence counseling and follow-up.

In China, BluedHealth, a leading internet-based health platform, has become a major channel for PrEP distribution. In 2023, BluedHealth reported an average of over 3,000 PrEP sales per month, reflecting increasing acceptance of online PrEP services. Despite this expansion, there is little evidence regarding adherence patterns and associated factors among real-world online PrEP consumers in China[6].

To address this gap, the Real-world E-Consumer Cohort of pre-exposure prophylaxis (RECOPE) is established as the first prospective cohort study targeting individuals purchasing PrEP online in China.

1. **Study Objective:**
2. **Primary Objective**

To evaluate medication adherence among real-world e-consumers of PrEP in China, with a focus on both event-driven (ED) and daily regimens, over baseline and three follow-up visits (1, 3, and 6 months), to identify socio-demogrphic and psycho-behavioral factors associated with PrEP adherence, including self-efficacy, PrEP-related stigma, depressive symptoms, and sexual behaviors (e.g., number of partners, chemsex, condom use, sexual role), and to provide evidence to inform interventions that optimize real-world PrEP effectiveness.

1. **Secondary Objectives**

- To assess the utilization of other HIV-related services (e.g., willingness to use long-acting injectable PrEP, HIV testing behavior)
- To evaluated the factors associated with the utilization of other HIV-related services.

1. **Study Design and Methodology**

**3.1 Study Design**

This study is a prospective cohort study designed to follow real-world e-consumers of pre-exposure prophylaxis (PrEP) in China. Eligible participants who purchased PrEP through BluedHealth, the largest internet-based platform for PrEP distribution in China, will be enrolled. Participants will complete a baseline survey and follow-up assessments at 1, 3, and 6 months.

**3.2 Sample Size Calculation**

The required sample size is calculated using Cochran’s formula for estimating a single population proportion, incorporating finite population correction and a design effect adjustment, as shown in Equation (1). Based on previous studies, we assumed an optimal adherence prevalence of 60%, a two-sided significance level of α = 0.05, and a desired precision (margin of error) of 5%.

A design effect (deff) is included to account for potential clustering introduced by the recruitment workflow, since participants will be conveniently recruited by the designated recruiters through the same platform channels may share unobserved similarities. Following the standard formula deff = 1 + (m – 1) × ICC, we assumed an average cluster size of approximately 11 participants per recruiter and a small intraclass correlation coefficient (ICC) of 0.01. This yielded deff ≈ 1.1, which is applied as a conservative adjustment [7,8].

In addition, a 15% anticipated loss to follow-up is included. The final minimum required sample size is calculated to be 424 participants using PASS software.

n = deff × [N p̂ q̂] / [(d² / 1.96²)(N – 1) + p̂ q̂] (1)

**3.3 Study Population**

Participants will be recruited through BluedHealth, the main online PrEP sales platform in China, operated by Beijing Blued Health Management Co., Ltd. The study population will consist of individuals in China who purchase PrEP online.

**Inclusion criteria:**

- Age ≥18 years.
- Purchased PrEP online via BluedHealth.
- Eligible for PrEP use according to national guidelines (e.g., HIV-negative, no contraindications to PrEP such as significant renal impairment, at high risk of acquiring HIV such as having condomless anal sex, having a HIV-positive partner whose viral load is not confirmed as suppressed, have been newly diagnosed with a sexually transmitted infection, inject drugs and share equipment, or have repeatedly used post-exposure prophylaxis).
- Planning to use PrEP over the next six months.
- Willing to provide informed consent.

**Exclusion criteria:**

- Communication barriers or reading disabilities reported by recruiters or researchers (e.g., being unable to read the invitation message or difficult to communicate with the recruiters).

**3.4 Recruitment Procedure**

Recruitment will be conducted in collaboration with BluedHealth. Staff from BluedHealth will distribute recruitment materials (texts and posters) containing a unique identification key and contact details. The unique keys will be generated using a four-digit code plus a letter (e.g., 1001c, 1002d), ensuring each participant can be individually tracked.

The first round of recruitment will target individuals who purchased PrEP within one month prior to study initiation. If fewer than 650 participants are enrolled within two weeks, subsequent rounds will expand eligibility to those who purchased PrEP within the past two or three months, until the target sample size is reached (ideally within 30 days).

Recruiters will contact eligible clients through BluedHealth customer service channels or directly at the time of PrEP purchase, inviting them to join the study. Interested individuals will scan a WeChat QR code to connect with study investigators. Prospective participants must include their unique key when adding the investigator to confirm identity. Investigators will verify inclusion/exclusion criteria, obtain informed consent, and finalize enrollment.

A reminder mechanism will be implemented: after receiving the QR code, potential participants will receive up to two reminders within 48 hours. If they do not respond, the key will be invalidated. Each participant’s unique key will serve as their identifier throughout the study(figure 1).


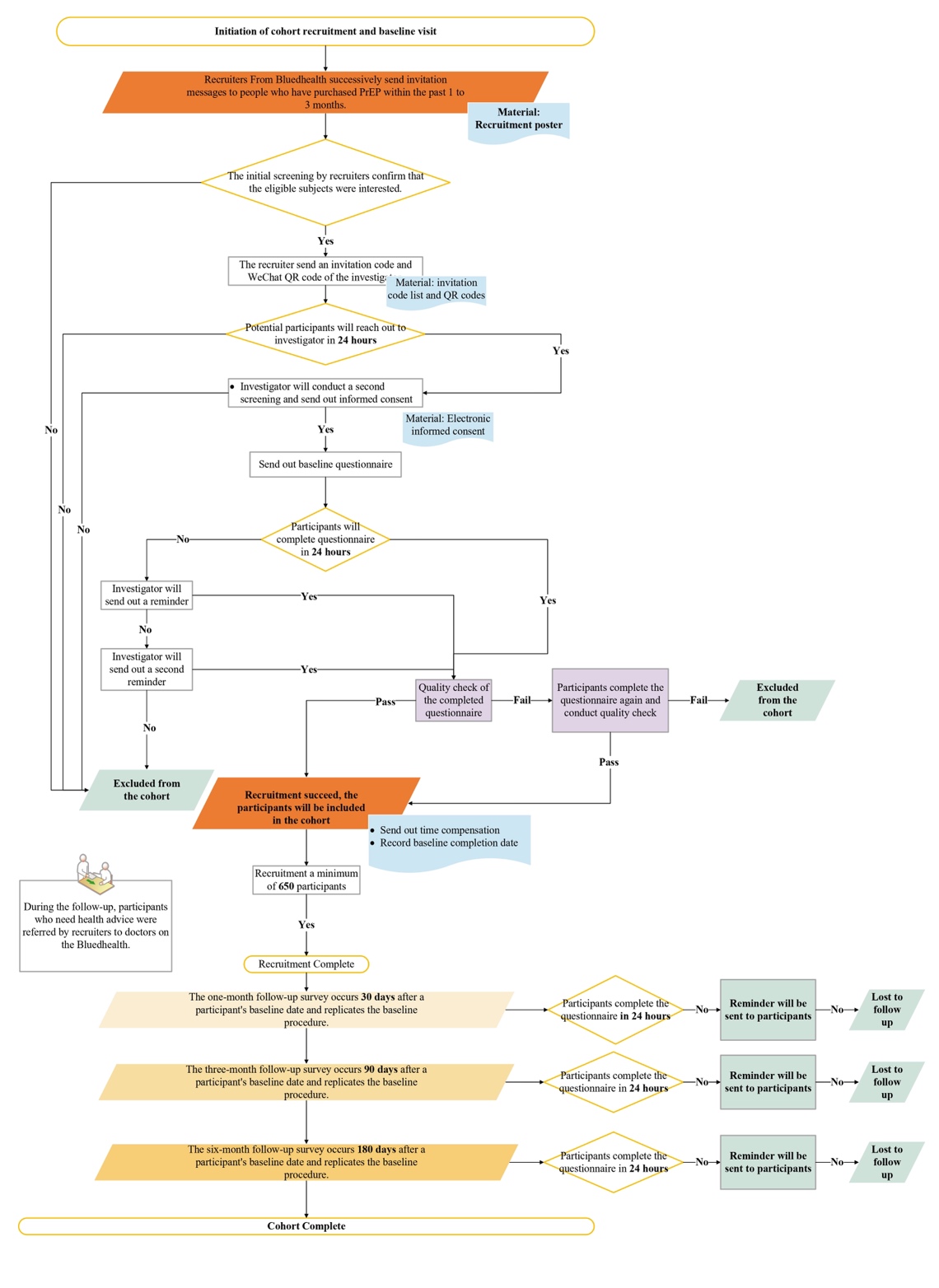


Figure 1 Flowchart of the study procedure

**3.5 Baseline and Follow-up Visits**

Following enrollment, participants will complete a baseline questionnaire delivered through Wenjuanxing (an online survey platform) via WeChat. The questionnaire covers socio-demographics, PrEP regimen, adherence, psychosocial measures, and sexual behaviors. Completion time is approximately 10-15 minutes. Data will undergo quality checks, and participants passing these checks will receive a 50 CNY BluedHealth voucher, valid for three months.

Follow-up assessments will occur at 1, 3, and 6 months after baseline. Survey links will be delivered individually via WeChat, and participants will again receive vouchers upon quality-verified completion.

**3.6 Questionnaires and Measures**

The baseline and follow-up survey questionnaires are developed based on the study aims, previous literature, and expert consultation. The draft questionnaires will be pilot tested among 5-10 e-consumers of PrEP, and be modified and finalized based on their comments and suggestion.

**Primary Outcome**

The primary outcome of this study is PrEP adherence, assessed separately for daily and event-driven (ED) regimen users at each follow-up visit (1, 3, and 6 months after baseline).

- **ED users:** Adherence to ED regimen is assessed by four items. First, the participants are asked whether they had engaged in sexual activity in the past one and three months, respectively (the 1-month follow-up survey only asked this question for the past one month). Those who answer “no” or reported to have never initiated PrEP in a previous question were treated as missing data for PrEP adherence. Those who answer “yes” are then asked if they had taken PrEP for each sexual encounter in the corresponding time period, including those with both primary (e.g., main or long-term partners) and non-primary (e.g., casual or commercial partners) sexual partners (1 = every time, 2 = sometimes, and 3 = never). The participants who choose “every time” or “sometimes” are subsequently asked three adherence questions based on the ‘2-1-1’ dosing guideline: (i) whether (yes or no) they took two pills of PrEP 2 to 24 hours before the sexual activity for each medication (timepoint A); (ii) whether they took one pill 24 hours after the first dose for each medication (timepoint B); and (iii) whether they took another pill 48 hours after the first dose for each medication (timepoint C). The participants who reported to take PrEP for each sexual encounter and chose “yes” to all three adherence questions are classified as “being adherent,” otherwise, they would be classified as “non-adherent.” Lastly, three binary outcome variables are constructed: past 1-month adherence based on baseline and all follow-up data, past 3-month adherence based on baseline and 3- and 6-months follow-up data, and consistent adherence based on 3- and 6-months follow-up data (defined as having optimal past 3-month adherence at both 3- and 6-month follow-up surveys).
- **Daily users:** Adherence to daily PrEP regimen is assessed with a single item: “In the past month, have you ever failed to take PrEP on time and in a correct dosage? (yes/no).” Participants who answer “no” are classified as “adherent,” and those who chose “yes” are categorized as “non-adherent”

**Other measures**

***Demographic characteristics***

The participants will be asked about their age (in years), sex (male, female), ethnicity (Han, others), employment status (students, unemployed, employed), education level (Junior high school or below, senior high school or equivalent, bachelor degree or equivalent, master degree or above), marital status (married/living with a partner, unmarried/divorced/separated/widowed), and monthly income (≤3000, 3001-5000, 5001-7000, 7001-10000, 10001-15000, 15001-20000, >20000 CNY). Income is further recoded into a binary variable using 3000 CNY (approximately 700 USD) as a threshold as it is the national average monthly income in China.

***Sexual behaviors***

The participants’ sexual behaviors are measured, including whether having had chemsex (Yes/ No), number of sexual partners, commercial sexual behavior (Yes/ No), roles in sexual intercourse (Insertive/ Receptive/ Versatile), and frequency of condoms use for each intercourse (Never/ Occasionally/ Sometimes/ Often/ Always) in the past follow-up months. For this study, frequency of condom use is re-categorized as inconsistent condom use (Never/ Occasionally/ Sometimes) and consistent condom use (Often/ Always).

***PrEP-related variables***

Regarding the participants’ understanding of PrEP dosing instructions, the ED PrEP users are asked “Which of the following are correct ways to take PrEP on demand?” They could choose responses from: Take 2 pills orally 2 to 24 hours before anticipated sexual activity; Take 1 pill orally 2 to 24 hours before anticipated sexual activity; Take 1 additional pill 24 hours after the initial dose following sexual activity; Take 1 additional pill 48 hours after the initial dose following sexual activity. The daily PrEP users are asked, “Which of the following are correct ways to take PrEP daily. They could choose responses from: Take 1 pill every 24 hours; Take 2 pills every 24 hours; High-risk behavior for HIV should only be undertaken after taking the medication daily for 7 consecutive days. The responses include Yes, No, and Uncertain. Only the participants who selected all the correct options are coded as “having correct PrEP adherence knowledge”.

PrEP-related stigma is measured by the Chinese version of HIV PrEP Stigma Scale (HPSS)[9]. The scale consisted of 11 items (e.g., “Someone taking PrEP would be seen by others slutty). Each item is rated on a 5-point Likert scale ranging from 1 (strongly disagree) to 5 (strongly agree). A summative score ranging from 11 to 55 is calculated by summing the scores of all items. After reverse scoring, a summative score is calculated, with higher total scores indicating greater stigma associated with taking PrEP.

***Social-psychological variables***

Self-efficacy of keeping optimal PrEP adherence is evaluated by an adapted version of the Condom Use Self-Efficacy Scale [10], which has been validated among Chinese MSM populations [11]. The adapted scale included six items related to consistent PrEP use (e.g., “Even if my sexual partner is unwilling, I am confident in continuing PrEP use”). Each item is rated on a 5-point Likert scale, ranging from 1 (strongly disagree) to 5 (strongly agree). A summative score ranging from 6 to 30 is created by adding up the score of each item, with a higher total score indicating greater self-efficacy in adhering to PrEP.

Depressive symptoms are assessed by the Patient Health Questionnaire-9 (PHQ-9) [12], which has been validated in Chinese MSM populations and demonstrated strong psychometric properties [13]. The questionnaire asked participants about the frequency of specific symptoms experienced over the past two weeks, with each item rated on a 4-point Likert scale ranging from 0 (not at all) to 3 (nearly every day). A summative score ranging from 0 to 27 is created by adding up the score of each item.

Psychological resilience is measured by the 2-item Connor-Davidson Resilience Scale (CD-RISC-2) [14], which has been validated among MSM populations [15]. The items included “Able to adapt to change” and “Tend to bounce back after illness or hardship”. Responses are rated on a 5-point Likert scale ranging from 0 (never) to 4 (always). A summative score ranging from 0 to 8 is created by adding up the scores of 2 items.

**3.7 Data Management and Quality Control**

Survey responses are automatically recorded on the Wenjuanxing platform and exported as encrypted files. All datasets are stored on password-protected institutional servers, accessible only to authorized study staff. Each participant is assigned a unique identifier at recruitment, which is used throughout all surveys to ensure anonymity. No personal identifiers are collected or stored.

To ensure data quality, each submission is reviewed for completeness, internal logic consistency, and reasonable completion time. Submissions failing these checks are excluded from analysis and do not qualify for incentives. Logic constraints are built into the questionnaire to minimize careless or contradictory responses.

During data management, double validation procedures are applied at the time of dataset export and cleaning. Backup copies are securely maintained, and audit trails are preserved to document data handling. All analytic datasets used for statistical analyses will be fully anonymized.

**3.8 Statistical Analysis Plan**

All statistical analyses will be pre-specified and conducted according to this plan. Analyses will distinguish between event-driven (ED) and daily PrEP regimen users. Both descriptive and inferential statistics will be used to evaluate adherence and associated factors across baseline, 1-, 3-, and 6-month follow-ups.

**Descriptive Analyses**

- **Baseline characteristics:** Continuous variables (e.g., age, psychosocial scores) will be summarized as means (standard deviations) or medians (interquartile ranges), depending on distribution. Categorical variables (e.g., education, income category, sexual role) will be summarized as frequencies and percentages.
- **Adherence prevalence:** The prevalence of optimal PrEP adherence at each wave will be calculated separately for ED and daily users.
- **Scale Reliability:** Internal consistency of psychosocial measures (e.g., PHQ-9, self-efficacy scale, stigma scale) will be evaluated at baseline using Cronbach’s alpha. Alpha values ≥0.80 will be considered acceptable.

**Regression Analyses**

**Event-driven (ED) users:** Three regression models will be fitted:

- Model 1: A univariable and multivariable generalized linear mixed model (GLMM) with a logit link and random intercept to evaluate factors associated with past three-month adherence using data from baseline, 3-month, and 6-month visits. In the multivariable GLMM, all the exposure variables of interest will be involved.
- Model 2: A univariable and multivariable GLMM with the same structure to evaluate factors associated with past one-month adherence using data from baseline and all follow-up visits.
- Model 3: A logistic regression model for consistent adherence, defined using data from the 3- and 6-month follow-ups, with baseline socio-demographics and averaged time-varying covariates included as independent variables.

**Daily users:** Regression analysis is planned only for past one-month adherence (Model 2), using a GLMM with the same structure as for ED users.

All models will include fixed effects for exposure variables of interest. Univariable GLMMs will be fitted first to estimate crude odds ratios (ORs) and 95% confidence intervals (CIs) for each covariate. Multivariable GLMMs will then be fitted including all pre-specified, theory-driven covariates—regardless of their univariable associations—to estimate independent effects while adjusting for potential confounding.

Missing data will be handled under a complete case framework. Specifically, missing data are expected to arise primarily from loss to follow-up. No imputation will be performed. GLMM models inherently allow for unbalanced data across repeated measures, and participants with incomplete follow-up will contribute all available observations. Attrition patterns will be described in supplementary analyses.

All analyses will be performed using R (version 4.4.2). Statistical significance will be defined as a two-sided p-value < 0.05.

1. **Ethics and Confidentiality**

The study protocol will be reviewed and approved by the Institutional Review Board of Tsinghua University. All procedures will be conducted in accordance with the ethical standards of the Declaration of Helsinki and relevant national guidelines.

Informed consent will be obtained electronically from all participants prior to enrollment. Consent is obtained through a two-step electronic informed consent process conducted anonymously: first, candidates received the e-consent form via WeChat and are enrolled only after replying “agree to participate”; second, the baseline e-questionnaire began with the same consent text and required click-to-consent (“I agree”) before any survey items are displayed. No names or direct identifiers are collected.

Confidentiality of participant data is strictly protected. Each participant is assigned a unique identification key at recruitment, which is used for all subsequent data collection and analysis. No personal identifiers (e.g., name, phone number, address) are collected. Survey data are stored in encrypted files on secure institutional servers with password protection and restricted access. Only authorized research staff have access to the datasets.

Data sharing will follow institutional and journal requirements. De-identified datasets may be made available to qualified researchers upon reasonable request and approval by the corresponding institutional review board.

1. **Anticipated Challenges/Limitations**

- First, the convenience sampling method used in the study (conveniently choosing Bluedhealth as the recruitment channel and conveniently recruited the participants from Bluedhealth PrEP clients) may cause limited generalization ability and selection bias in the study.
- Second, the reliance on self-reported measures to assess PrEP adherence and other factors may introduce recall and social desirability biases. Besides, because participants are repeatedly surveyed at 1, 3, and 6 months, the follow-up process itself may have heightened their awareness of PrEP adherence and modestly influenced self-reported adherence. This monitoring effect could contribute to overestimation of adherence and should be considered when interpreting our results.
- Third, some other factors potentially associated with PrEP adherence (e.g., risk perception, peer support, prior health service use) are not included in the study and need further investigation.

1. **Dissemination Plan**

The findings from this study will be disseminated through multiple channels. Results will be submitted for publication in peer-reviewed journals and presented at national and international scientific conferences in the fields of HIV prevention and public health. Summaries of key findings will also be shared with community partners, relevant non-governmental organizations, and public health authorities in China to inform PrEP implementation strategies.

References:

1. UNAIDS. Global data on HIV epidemiology and response. In 2024. Available from: https://kpatlas.unaids.org/dashboard

2. Han MJ. Analysis of the epidemic situation of AIDS in China and prospects for its prevention and control. Chinese Journal of AIDS & STD. 2023;29(03):247–50.

3. Fonner VA, Dalglish SL, Kennedy CE, Baggaley R, O’Reilly KR, Koechlin FM, et al. Effectiveness and safety of oral HIV preexposure prophylaxis for all populations. AIDS. 2016 Jul 31;30(12):1973–83.

4. WHO. Guideline on when to start antiretroviral therapy and on pre-exposure prophylaxis for HIV. In 2015. Available from: https://www.who.int/publications/i/item/9789241509565

5. Jun-Jie X, Xiao-Jie H, Xin-Chao L, Li-Ming W, Yao-Kai C, Hui W, et al. Consensus statement on human immunodeficiency virus pre-exposure prophylaxis in China. Chinese Medical Journal [Internet]. 2020 Dec 5 [cited 2024 Sep 26]; Available from: https://mednexus.org/doi/10.1097/CM9.0000000000001181

6. Shan D, Xue H, Yu F, Zan X, Liu H, Liu J, et al. Understanding the Uptake and Outcomes of Non-occupational Postexposure Prophylaxis Use Through an Online Medical Platform in China: Web-Based Cross-sectional Study. Journal of Medical Internet Research. 2023 May 19;25(1):e42729.

7. Sulaberidze, L., Mirzazadeh, A., Chikovani, I., Shengelia, N., Tsereteli, N., & Gotsadze, G. (2016). Population Size Estimation of Men Who Have Sex with Men in Tbilisi, Georgia; Multiple Methods and Triangulation of Findings. PloS one, 11(2), e0147413. https://doi.org/10.1371/journal.pone.0147413.

8. Wendland, E., Vieira, B. A., Eidt, G., Ikeda, M. L. R., de Souza, F. M. A., Tonini, M. L., Gaspar, P. C., Hallal, R. C., Moherdaui, F., Pereira, G. F. M., & Bidinotto, A. B. (2025). A serological household survey on social determinants of the generalized HIV epidemic in southern Brazil. Scientific reports, 15(1), 25476. https://doi.org/10.1038/s41598-025-06764-6.

9. Siegler AJ, Wiatrek S, Mouhanna F, Amico KR, Dominguez K, Jones J, et al. Validation of the HIV Pre-exposure Prophylaxis Stigma Scale: Performance of Likert and Semantic Differential Scale Versions. AIDS Behav. 2020 Sep 1;24(9):2637–49.

10. Brafford LJ, Beck KH. Development and Validation of a Condom Self-Efficacy Scale for College Students. Journal of American College Health. 1991 Mar 1;39(5):219–25.

11. Sun S, Yang C, Zaller N, Zhang Z, Zhang H, Operario D. PrEP Willingness and Adherence Self-Efficacy Among Men Who have Sex with Men with Recent Condomless Anal Sex in Urban China. AIDS Behav. 2021 Nov 1;25(11):3482–93.

12. Kroenke K, Spitzer RL, Williams JBW. The PHQ‐9: validity of a brief depression severity measure. Journal of General Internal Medicine. 2001;16(9):606–13.

13. Li Q, Zhang Y, Huang S, Xu Y, Zhou J, Li Y, et al. Depressive symptoms and its multifaceted associated factors among young men who have sex with men facing the dual threats of COVID-19 and mpox in China. J Affect Disord. 2024 Oct 15;363:39–46.

14. Vaishnavi S, Connor K, Davidson JRT. An abbreviated version of the Connor-Davidson Resilience Scale (CD-RISC), the CD-RISC2: Psychometric properties and applications in psychopharmacological trials. Psychiatry Research. 2007 Aug 30;152(2):293–7.

15. Dale SK, Sanders J, Safren SA, Ironson G, O’Cleirigh C. Correlates of resilience after childhood sexual abuse among men who have sex with men. Journal of Trauma & Dissociation. 2020 May 26; Available from: https://www.tandfonline.com/doi/abs/10.1080/15299732.2020.1719263
