# Supplementary material for: Medication adherence and its associated factors among oral pre-exposure prophylaxis (PrEP) users in China: The Real-world E-consumer Cohort of PrEP study
Source: PLoS Med. 2026 Feb 26;23(2):e1004733. doi: 10.1371/journal.pmed.1004733 (PMC12944781; doi:10.1371/journal.pmed.1004733)
Supplement: S2 File — This file shows the survey tools for data collection, including items on demographic characteristics, sexual behaviors, psychosocial measures, and PrEP-related variables. (DOCX) [file pmed.1004733.s011.docx]

**Baseline and Follow-up Survey Questionnaire of the RECOPE study**

1. **Sociodemographic Information**
2. Your birth year and birth month is： Year: _____ Month: _____
3. Your assigned sex at birth is:

○Male

○Female

1. Your currently live in：

○Urban area

○Rural area

1. Your ethnicity is：

○Han ○Others, please specify______

1. What is your current occupation category？

○Student

○Head of state agency/enterprise

○Professional and technical personnel

○Officers and related personnel

○Business and service industry personnel

○Agriculture, forestry, animal husbandry, fishing, water conservancy production personnel

○Production and transportation equipment operators and related personnel

○Military personnel

○Unemployed

○Others, please specify______

1. The highest level of school you have completed or currently working on

○Elementary school and below

○Middle school

○High school/professional high school/technical secondary school

○Undergraduate/Community College

○Graduate School (Masters' degree and above)

1. What is your monthly income?

○3000 CNY and below

○3001 - 5000 CNY

○5001-7000 CNY

○7001-10000 CNY

○10001-15000 CNY

○15001-20000 CNY

○20000 CNY and above

1. What is your current marital status?

○Married or Co-living with a partner

○Single

○Divorced or Separated with a partner

○Widow

1. Which gender best describes yourself?

○Male

○Female

○Transgender men

○Transgender women

○Nonbinary gender

○Other

1. **PrEP related Behavior**
2. Have you ever taken PrEP before?？

○Yes（skipped to 16）

○No

1. When do you plan to start taking PrEP？

○In the next month

○In the next 3 months

○In the next 6 months

○Don't know/Not sure

1. Which dosing regimen do you plan to use？

○Daily （skip to 14）

○On Demand（or Event Driven）（skip to 13）

○Not sure/Don’t know（skip to 15）

1. What is the correct answer for events driven use PrEP dosing regime：（skip to 15）

- Take 2 pills 2 -24 hours before the event
- Take 1 pill 2 -24 hours before the event
- Take another pill after the event, at the 24th hour after taking the first pill
- Take another pill after the event, at the 48th hour after taking the first pill
- Don't know/Not sure（Exclusive）

1. What is the correct answer for daily use PrEP dosing regime：

- Take 1 pill every 24 hour
- Take 2 pills every 24 hour
- You need to take PrEP for 7 days consecutively before having high risk activity
- You need to take PrEP for another 7 days after the last high-risk activity
- You can stop taking PrEP immediately after the last high-risk activity
- Don't know/Not sure（Exclusive）

1. When you start taking PrEP, how likely do you feel that you will adhere to PrEP as prescribed？

○Very unlikely（skip to 35）

○Unlikely（skip to 35）

○Half & Half（skip to 35）

○Likely（skip to 35）

○Very likely（skip to 35）

1. What is your first time taken PrEP ever: Year: _____ Month:_____
2. Which dosing plan are you currently on？

○Daily （skip to 29）

○On-demand（or event-driven）

1. What is the correct answer for events driven use PrEP dosing regime：（skip to 15）

- Take 2 pills 2 -24 hours before the event
- Take 1 pill 2 -24 hours before the event
- Take another pill after the event, at the 24th hour after taking the first pill
- Take another pill after the event, at the 48th hour after taking the first pill
- Don't know/Not sure（Exclusive）

1. In the past three months, have you used PrEP each time you engaged in sexual activity?

○I have not had sex in the past three months （skip to 32）

○I have had sex within the past three months and took PrEP each time

○I have had sex within the past three months but did not take PrEP every time


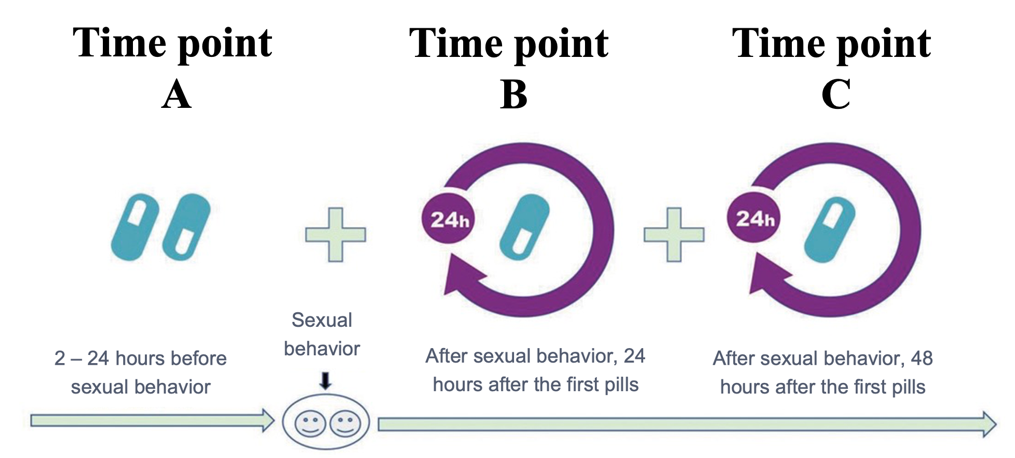


1. In the past three months, have you used PrEP at time point A you engaged in sexual activity？

○Yes

○No

1. In the past three months, have you used PrEP at time point B you engaged in sexual activity?

○Yes

○No

1. In the past three months, have you used PrEP at time point C you engaged in sexual activity?

○Yes

○No

1. In the past three months, what are the reasons for not taking PrEP as instructed?

- I didn't know the correct way of taking PrEP
- I forgot to take PrEP
- I don't have access to PrEP in a timely manner
- Adverse side effects
- I worry that PrEP will interact with my other treatment
- I have concerns about privacy
- People around me are not supportive
- I found taking PrEP is inconvenient
- I used other source of protections (e.g., condoms)
- Other, please specify _______
- N/A (Exclusive)

1. In the past one months, have you used PrEP each time you engaged in sexual activity?

○I have not had sex in the past month （skip to 32）

○I have had sex within the past month and took PrEP each time

○I have had sex within the past month but did not take PrEP every time


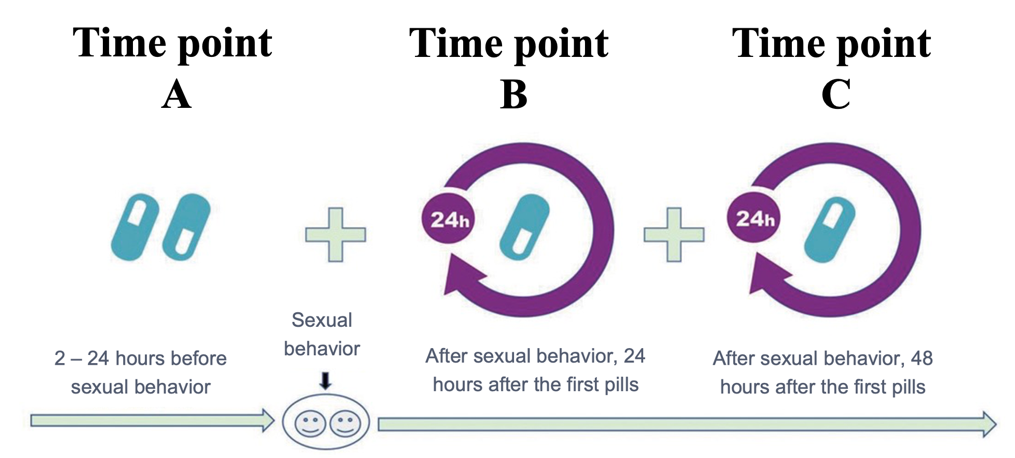


1. In the past month, have you used PrEP at time point A you engaged in sexual activity？

○Yes

○No

1. In the past month, have you used PrEP at time point B you engaged in sexual activity?

○Yes

○No

1. In the past month, have you used PrEP at time point C you engaged in sexual activity?

○Yes

○No

1. In the past month, what are the reasons for not taking PrEP as instructed （skip to 32）

- I didn't know the correct way of taking PrEP
- I forgot to take PrEP
- I don't have access to PrEP in a timely manner
- Adverse side effects
- I worry that PrEP will interact with my other treatment
- I have concerns about privacy
- I trust my partner
- I found taking PrEP is inconvenient
- I used other source of protections (e.g., condoms)
- Other, please specify _______
- N/A (Exclusive)

1. What is the correct answer for daily use PrEP dosing regimen：

- Take 1 pill every 24 hour
- Take 2 pills every 24 hour
- You need to take PrEP for 7 days consecutively before having high risk activity
- You need to take PrEP for another 7 days after the last high-risk activity
- You can stop taking PrEP immediately after the last high-risk activity
- Don't know/Not sure（Exclusive）

1. In the past one month, did you have any occasions when you did not take PrEP on time or following the recommended dosage?

○Yes，the number of pills I have missed is ______（enter a digit）

○No（skip to 32）

1. In the past month, what are the reasons for not taking PrEP as instructed?

- I didn't know the correct way of taking PrEP
- I forgot to take PrEP
- I don't have access to PrEP in a timely manner
- Adverse side effects
- I worry that PrEP will interact with my other treatment
- I have concerns about privacy
- I trust my partner
- My sexual encounters were not frequent
- I found taking PrEP is inconvenient
- I used other source of protections (e.g., condoms)
- Other, please specify _______

1. Have you switched PrEP regime?

- I switched from event driven use to daily use
- I switched from daily use to event driven use
- I switched to Post-exposure Prophylaxis (PEP)
- No

1. Have you experienced any side effects/adverse reactions while taking PrEP?

○Never（skip to 35）

○Rarely

○Occasionally

○Often

○Almost every time

1. What side effects/adverse reactions have you experienced while taking PrEP?

- Dizziness and/or headache
- Nausea and/or vomiting
- Abdominal pain and/or diarrhea
- Liver and kidney damage
- Fever
- Fatigue/drowsiness
- Insomnia/dreaming
- Skin rash
- Anxiety, depression and other mental disorders
- Other side effects/adverse reactions, please specify _______

1. **PrEP knowledge and attitude**
2. PrEP knowledge（8 questions and 1 attention check question）

| Regarding the following questions, please select the option that best suits you： | Correct | Wrong | Don’t know |
| --- | --- | --- | --- |
| 1. When you miss a dose of PrEP, you must take twice the dose the next time you take it | 1 | 2 | 0 |
| 1. If you are tested positive for HIV while taking PrEP medication, you need to stop taking the medication immediately and start antiviral treatment as soon as possible. | 1 | 2 | 0 |
| 1. For PrEP daily medication regimen, unprotected sex is not allowed immediately after starting | 1 | 2 | 0 |
| 1. This is an attention check question, please choose ‘correct’ | 1 | 2 | 0 |
| 1. For an on-demand regimen of PrEP, if the interval between the last dose and the next sexual intercourse is <7 days, resume taking 1 tablet daily until 48 hours after the last sexual intercourse. | 1 | 2 | 0 |
| 1. Before taking PrEP, you must go to a professional medical institution for an HIV test to ensure that you are not infected with HIV. | 1 | 2 | 0 |
| 1. It may lead to the risk of drug resistance and serious adverse consequences of their subsequent treatment if infected but undiagnosed HIV-positive patients take PrEP | 1 | 2 | 0 |
| 1. All individuals taking PrEP should be tested for HIV regularly | 1 | 2 | 0 |
| 1. All individuals taking PrEP should have their kidney function and hepatitis B virus antigen and antibodies tested regularly. | 1 | 2 | 0 |

1. Perceived Benefits, Barriers, and self-efficacy（12 questions）

| For the following statements about maintaining good adherence to PrEP, please indicate your level of agreement： | Strongly disagree | Disagree | Neutral | Agree | Strongly agree |
| --- | --- | --- | --- | --- | --- |
| **Perceived Benefits** |  |  |  |  |  |
| 1. Only by maintaining good PrEP medication compliance can I protect myself from HIV infection. | 1 | 2 | 3 | 4 | 5 |
| 1. Only by maintaining good PrEP medication compliance can I protect those around me from HIV infection. | 1 | 2 | 3 | 4 | 5 |
| **Perceived Barriers** |  |  |  |  |  |
| 1. I am worried about the side effects of taking PrEP. | 1 | 2 | 3 | 4 | 5 |
| 1. I am worried that my sexual partner will find out I am taking medication. | 1 | 2 | 3 | 4 | 5 |
| 1. I find it troublesome to take medicine. | 1 | 2 | 3 | 4 | 5 |
| **Perceived Self-efficacy** |  |  |  |  |  |
| 1. I feel confident taking PrEP while I am/have recently been drinking alcohol or using other drugs | 1 | 2 | 3 | 4 | 5 |
| 1. Even if my sexual partner is not happy, I still have the confidence to continue taking PrEP | 1 | 2 | 3 | 4 | 5 |
| 1. I still have the confidence to keep taking PrEP even when I feel it has side effects | 1 | 2 | 3 | 4 | 5 |
| 1. When using PrEP causes me too much trouble, I still have the confidence to keep taking it | 1 | 2 | 3 | 4 | 5 |
| 1. I feel confident continuing to take PrEP when I believe my risk of contracting HIV is low | 1 | 2 | 3 | 4 | 5 |
| 1. I feel confident that I can continue taking PrEP when I have used other protective measures (such as condoms) PrEP | 1 | 2 | 3 | 4 | 5 |

1. PrEP Stigma（11 questions）

| For the following statements about using PrEP, please indicate your level of agreement: | Strongly disagree | Disagree | Neutral | Agree | Strongly agree |
| --- | --- | --- | --- | --- | --- |
| 1. I would feel ashamed to take PrEP pills in front of others | 1 | 2 | 3 | 4 | 5 |
| 1. Someone taking PrEP should keep their pills hidden | 1 | 2 | 3 | 4 | 5 |
| 1. People experience negative judgment because they take PrEP | 1 | 2 | 3 | 4 | 5 |
| 1. Someone taking PrEP would be seen by others as slutty | 1 | 2 | 3 | 4 | 5 |
| 1. People taking PrEP receive praise for being responsible | 1 | 2 | 3 | 4 | 5 |
| 1. My friends would be supportive of me taking PrEP | 1 | 2 | 3 | 4 | 5 |
| 1. People experience problems when they tell their sex partner(s) they are taking PrEP | 1 | 2 | 3 | 4 | 5 |
| 1. I would feel proud to take PrEP every day | 1 | 2 | 3 | 4 | 5 |
| 1. People taking PrEP experience verbal harassment | 1 | 2 | 3 | 4 | 5 |
| 1. People on PrEP are taking care of their health | 1 | 2 | 3 | 4 | 5 |
| 1. My family would be supportive of me taking PrEP | 1 | 2 | 3 | 4 | 5 |

1. Your current average spending on PrEP is ___ CNY/month
2. How much of a financial burden does purchasing PrEP currently place on you?

○Very low

○Low

○Moderate

○High

○Very high

1. Do you use or intend to use tools or ways to remind yourself to take PrEP medication? (e.g., cell phone alarm clock, electronic pill box, or reminder from friends or relatives)

○Yes，please specify______

○No

1. Which channel did you use to obtain information about purchasing PrEP online？

- Television, traditional newspapers or magazines (e.g., News Feed, People's Daily, etc.)
- Official government websites (e.g., the website of the Center for STD and AIDS Prevention and Control of the Chinese Center for Disease Control and Prevention, the website of the Health Commission, etc.)
- Non-governmental news sites (e.g. Tencent News, Netease News, etc.)
- Social media platform tweets (e.g. WeChat, Jitterbug, Blued, etc.)
- Interaction with professionals (e.g. medical personnel/staff of social organizations)
- Communication with family/friends/colleagues/classmates and other non-professionals
- Other channels

1. Attitude toward purchasing PrEP online（6 questions and 1 attention check question）

| Which of the following descriptions most closely matches your opinion? | Strongly disagree | Somewhat disagree | Slightly disagree | Unsure | Slightly agree | Somewhat agree | Strongly agree |
| --- | --- | --- | --- | --- | --- | --- | --- |
| 1. I am very happy with my internet purchase of PrEP services | 0 | 1 | 2 | 3 | 4 | 5 | 6 |
| 1. The experience of using the Internet to purchase PrEP services exceeded my expectations | 0 | 1 | 2 | 3 | 4 | 5 | 6 |
| 1. I can trust the internet platform selling PrEP to properly address any issues that may arise | 0 | 1 | 2 | 3 | 4 | 5 | 6 |
| 1. This is an attention check question, please choose ‘strongly agree’ | 0 | 1 | 2 | 3 | 4 | 5 | 6 |
| 1. I find the Internet convenient for purchasing PrEP services | 0 | 1 | 2 | 3 | 4 | 5 | 6 |
| 1. I have more trust in the Internet for purchasing PrEP services than in other ways | 0 | 1 | 2 | 3 | 4 | 5 | 6 |
| 1. Platforms for purchasing PrEP services on the Internet do not leak my privacy | 0 | 1 | 2 | 3 | 4 | 5 | 6 |

1. Have you heard of the long-acting injectable PrEP？

○Yes

○No

1. If the long-acting injectable PrEP is available in China, would you be willing to use it?

○Strongly unwillingly

○Unwillingly

○Not sure

○Willingly （skip to 46）

○Strongly willingly （skip to 46）

1. The reason that you are hesitant to use long-acting injectable PrEP？

- The cost might be high（skip to 47）
- Uncomfortable experience/pain from injections（skip to 47）
- Side effects after injections（skip to 47）
- Prefer other ways of preventing HIV infection (e.g., oral PrEP, condom use during sex) （skip to 47）
- Other, please specify____（skip to 47）

1. The reason that you are willing to use long-acting injectable PrEP？

- Perceived as more convenient (e.g., does not require daily medication, etc.）
- Considered to provide long-term prevention of HIV infection (i.e., longer drug protection).
- The modality is considered to have pharmacokinetic advantages (meaning that the drug is better absorbed, distributed, metabolized and eliminated in the body)
- Considered to have better privacy
- Other, please specify____

1. **Lifestyles & HIV testing**
2. Current alcohol use

○Never（skip to 49）

○No more than once a month

○2-4 times a month

○2-3 times a week

○More than 4 times a week

1. In the last 3 months, have you had 5 cans of beer, or 5 small glasses of liquor, or 5 glasses of red/white wine in one day?

○Yes

○No

1. Do you smoke currently?

○Cigarette

○Vape

○Other

○Multiple products

○Used to smoke, now quit

○Never smoke

1. In the past 3 months, have you used any of the following substance?

○Hallucinogens

○Inhalers

○Cannabis

○Amphetamine stimulants

○None of the above substances

1. In the past 3 months, have you had sexual intercourse (including vaginal, anal, or oral sex)?

○Yes, only homosexual

○Yes, only heterosexual（skip to 52）

○Yes, both

○No（skip to 56）

1. In the past 3 months, how many homosexual partners did you have? ________ (Enter digit greater than 0)
2. In the past 3 months, when you had sex with your same-sex partner, your sex role was?

○Insertive

○Mainly Insertive

○Both

○Mainly Receptive

○Receptive

1. In the past 3 months, how often did you use condoms when having sex with a same gender partner?

○Never

○Rarely

○Sometimes

○Often

○Always

1. In the past 3 months, did you have any commercial sex with a same gender partner?

○Yes, I have gained money or resources by having sex with others

○Yes, I have offered money or resources by having sex with others

○No

1. In the past 3 months, did you use any of the following substance during sex with a same gender partner?

○Hallucinogens

○Inhalers

○Cannabis

○Amphetamine stimulants

○None of the above substances

1. In the past 3 months, how many heterosexual partners did you have? ________ (Enter digit greater than 0)

○N/A（skip to 61）

1. In the past 3 months, how frequently have you used condoms during sexual activity with heterosexual partners?

○Never

○Rarely

○Sometimes

○Often

○Always

1. In the past 3 months, did you have any commercial sex with a different gender partner?

○Yes, I have gained money or resources by having sex with others

○Yes, I have offered money or resources by having sex with others

○No

1. In the past 3 months, did you use any of the following substance during sex with a different gender partner?

○Hallucinogens

○Inhalers

○Cannabis

○Amphetamine stimulants

○None of the above substances

1. When you initially planned to use PrEP, did you undergo HIV testing?

○Yes

○No

1. If you are already using PrEP, have you been regularly following the PrEP user guidelines for HIV testing (every three months)?

○Yes

○Yes, I undergo regular testing, but my testing frequency is more than three months

○No, I do not get HIV testing.

○I have not started taking PrEP yet

1. Have you undergone an HIV test in the past 3 months?

○Yes

○No

1. What was the result of your most recent HIV test?

○Never been tested for HIV

○Negative

○Positive

○Uncertain or don't know

1. Have you had any of the following sexually transmitted infections in the past 3 months?

○Syphilis

○Gonorrhea

○Genital Warts

○Genital Herpes

○Genital Chlamydia

○Hepatitis B

○Hepatitis C

○Other sexually transmitted infections, please specify________

○None of the above

1. How has your sleep been in the past month?

○Normal

○Occasionally insomnia (less than twice per month)

○Sometimes insomnia (1-2 times per week)

○Frequent insomnia (3-5 times per week)

○Almost every day insomnia

1. How often have you engaged in physical exercise or fitness activities in the past month?

○None

○Occasionally (less than twice per month)

○Sometimes (1-2 times per week)

○Frequently (3-5 times per week)

○Almost every day

1. **Psychosocial questions**
2. Depression symptoms （10 questions）（PHQ-9）+attention check question

| Over the past two weeks, how often have you been bothered by any of the following problems? | Not at all | Several days | More than half the days | Nearly every day |
| --- | --- | --- | --- | --- |
| 1. Unable to get going or have fun when doing things | 0 | 1 | 2 | 3 |
| 1. Feeling down, depressed, or hopeless | 0 | 1 | 2 | 3 |
| 1. Difficulty falling asleep, staying asleep, or sleeping too much | 0 | 1 | 2 | 3 |
| 1. This is an attention check questions, please choose ‘nearly every day’ | 0 | 1 | 2 | 3 |
| 1. Feeling tired or having little energy | 0 | 1 | 2 | 3 |
| 1. Poor appetite or overeating | 0 | 1 | 2 | 3 |
| 1. Feeling bad about yourself, feeling like a failure, or letting yourself or your family down | 0 | 1 | 2 | 3 |
| 1. Difficulty concentrating on things, such as reading the newspaper or watching TV | 0 | 1 | 2 | 3 |
| 1. Moving or speaking so slowly that others have noticed, or the opposite - being fidgety or restless more than usual | 0 | 1 | 2 | 3 |
| 1. Thoughts of being better off dead or hurting yourself in some way | 0 | 1 | 2 | 3 |

1. Resilience (2 questions)

| Based on your experiences in the past month, please choose the statement below that best describes your situation | Never | Rarely | Sometimes | Often | Always |
| --- | --- | --- | --- | --- | --- |
| 1. I can adapt to changes. | 0 | 1 | 2 | 3 | 4 |
| 1. After going through hardships or illness, I usually recover quickly. | 0 | 1 | 2 | 3 | 4 |
