# Supplementary material for: Medication adherence and its associated factors among oral pre-exposure prophylaxis (PrEP) users in China: The Real-world E-consumer Cohort of PrEP study
Source: PLoS Med. 2026 Feb 26;23(2):e1004733. doi: 10.1371/journal.pmed.1004733 (PMC12944781; doi:10.1371/journal.pmed.1004733)
Supplement: S2 Checklist — This file summarizes how the study adheres to the Consensus-Based Checklist for Reporting of Survey Studies (CROSS) reporting guidelines. (DOCX) [file pmed.1004733.s013.docx]

**S2 Checklist. Checklist for Reporting Of Survey Studies (CROSS)**

| **Section/topic** | **Item** | **Item description** | **Reported on page #** |
| --- | --- | --- | --- |
| **Title and abstract** | | | **Page 2-3** |
| Title and abstract | 1a | State the word “survey” along with a commonly used term in title or abstract to introduce the study’s design. | Page 2-3, Abstract - Methods and findings |
|  | 1b | Provide an informative summary in the abstract, covering background, objectives, methods, findings/results, interpretation/discussion, and conclusions. | Page 2-3, Abstract |
| **Introduction** | | | **Page 5-7** |
| Background | 2 | Provide a background about the rationale of study, what has been previously done, and why this survey is needed. | Page 5-7, Introduction |
| Purpose/aim | 3 | Identify specific purposes, aims, goals, or objectives of the study. | Page 7, Introduction – paragraph 6 |
| **Methods** | | | **Page 7-15** |
| Study design | 4 | Specify the study design in the methods section with a commonly used term (e.g., cross-sectional or longitudinal). | Page 7, Methods - Study Design |
|  | 5a | Describe the questionnaire (e.g., number of sections, number of questions, number and names of instruments used). | Page 9-14, Methods - Measures |
| Data collection methods | 5b | Describe all questionnaire instruments that were used in the survey to measure particular concepts. Report target population, reported validity and reliability information, scoring/classification procedure, and reference links (if any) | - Page 12-14, Methods – Measures - PrEP-related variables – paragraph 2 and 3 - Page 12-14, Methods – Measures - Social-psychological variables |
|  | 5c | Provide information on pretesting of the questionnaire, if performed (in the article or in an online supplement). Report the method of pretesting, number of times questionnaire was pre-tested, number and demographics of participants used for pretesting, and the level of similarity of demographics between pre-testing participants and sample population. | N/A |
|  | 5d | Questionnaire if possible, should be fully provided (in the article, or as appendices or as an online supplement). | Online supplement, S2 File |
| Sample characteristics | 6a | Describe the study population (i.e., background, locations, eligibility criteria for participant inclusion in survey, exclusion criteria). | Page 8-9, Methods - Participant Recruitment – paragraph 2 |
|  | 6b | Describe the sampling techniques used (e.g., single stage or multistage sampling, simple random sampling, stratified sampling, cluster sampling, convenience sampling). Specify the locations of sample participants whenever clustered sampling was applied. | Page 8-9, Methods - Participant Recruitment – paragraph 1 |
|  | 6c | Provide information on 7, along with details of sample size calculation. | S1 File, Section 3.2 |
|  | 6d | Describe how representative the sample is of the study population (or target population if possible), particularly for population-based surveys. | Page 31-32, Discussion – paragraph 9 |
| Survey  administration | 7a | Provide information on modes of questionnaire administration, including the type and number of contacts, the location where the survey was conducted (e.g., outpatient room or by use of online tools, such as SurveyMonkey). | Page 9-10, Methods - Baseline and Follow-up Data Collection |
|  | 7b | Provide information of survey’s time frame, such as periods of recruitment, exposure, and follow-up days. | Page 7-8, Methods - Study Design |
|  | 7c | Provide information on the entry process:  –>For non-web-based surveys, provide approaches to minimize human error in data entry.  –>For web-based surveys, provide approaches to prevent “multiple participation” of participants. | Page 9-10, Methods - Baseline and Follow-up Data Collection |
| Study preparation | 8 | Describe any preparation process before conducting the survey (e.g., interviewers’ training process, advertising the survey). | Page 9-10, Methods - Baseline and Follow-up Data Collection |
| Ethical considerations | 9a | Provide information on ethical approval for the survey if obtained, including informed consent, institutional review board [IRB] approval, Helsinki declaration, and good clinical practice [GCP] declaration (as appropriate). | Page 7-8, Methods - Study Design |
|  | 9b | Provide information about survey anonymity and confidentiality and describe what mechanisms were used to protect unauthorized access. | Page 9, Methods - Participant Recruitment – paragraph 3 |
| Statistical  analysis | 10a | Describe statistical methods and analytical approach. Report the statistical software that was used for data analysis. | Page 14-15, Methods - Statistical Analysis |
|  | 10b | Report any modification of variables used in the analysis, along with reference (if available). | - Page 12-14, Methods – Measures - PrEP-related variables – paragraph 2 and 3 - Page 12-14, Methods – Measures - Social-psychological variables |
|  | 10c | Report details about how missing data was handled. Include rate of missing items, missing data mechanism (i.e., missing completely at random [MCAR], missing at random [MAR] or missing not at random [MNAR]) and methods used to deal with missing data (e.g., multiple imputation). | Page 14-15, Methods - Statistical Analysis-paragraph 3 |
|  | 10d | State how non-response error was addressed. | Page 31-32, Discussion – paragraph 9 |
|  | 10e | For longitudinal surveys, state how loss to follow-up was addressed. | - Page 16, Results - Sample characteristics - paragraph 1 - Page 16, Results - Sample characteristics – figure 1 |
|  | 10f | Indicate whether any methods such as weighting of items or propensity scores have been used to adjust for non-representativeness of the sample. | Page 15-16, Methods - Statistical Analysis |
|  | 10g | Describe any sensitivity analysis conducted. | N/A |
| **Results** | | | **Page 16-26** |
| Respondent characteristics | 11a | Report numbers of individuals at each stage of the study. Consider using a flow diagram, if possible. | Page 16, Results - Sample characteristics - paragraph 1  Page 16, Results - Sample characteristics – figure 1 |
|  | 11b | Provide reasons for non-participation at each stage, if possible. | Page 16, Results - Sample characteristics – figure 1 |
|  | 11c | Report response rate, present the definition of response rate or the formula used to calculate response rate. | Page 16, Results - Sample characteristics - paragraph 1 |
|  | 11d | Provide information to define how unique visitors are determined. Report number of unique visitors along with relevant proportions (e.g., view proportion, participation proportion, completion proportion). | Page 16, Results - Sample characteristics - paragraph 1 |
| Descriptive  results | 12 | Provide characteristics of study participants, as well as information on potential confounders and assessed outcomes. | - Page 16-17, Results - Sample characteristics - paragraph 2 - Page 18-19, Results - Sample characteristics – Table 1 - Page 20, Results - Distributions of Exposure Variables - paragraph 2 - Page 21-22, Results - Distributions of Exposure Variables - Table 2 |
| Main findings | 13a | Give unadjusted estimates and, if applicable, confounder-adjusted estimates along with 95% confidence intervals and p-values. | Page 26, Results – Table 3 |
|  | 13b | For multivariable analysis, provide information on the model building process, model fit statistics, and model assumptions (as appropriate). | Page 26, Results – Table 3 |
|  | 13c | Provide details about any sensitivity analysis performed. If there are considerable amount of missing data, report sensitivity analyses comparing the results of complete cases with that of the imputed dataset (if possible). | N/A |
| **Discussion** | | | **Page 27-33** |
| Limitations | 14 | Discuss the limitations of the study, considering sources of potential biases and imprecisions, such as non-representativeness of sample, study design, important uncontrolled confounders. | Page 31-33, Discussion – paragraph 9 |
| Interpretations | 15 | Give a cautious overall interpretation of results, based on potential biases and imprecisions and suggest areas for future research. | Page 33, Discussion – paragraph 10 |
| Generalizability | 16 | Discuss the external validity of the results. | Page 31-33, Discussion – paragraph 9 |
| **Other sections** | | |  |
| Role of funding source | 17 | State whether any funding organization has had any roles in the survey’s design, implementation, and analysis. | Page 33, Acknowledgments |
| Conflict of interest | 18 | Declare any potential conflict of interest. | Reported in the submission system |
| Acknowledgements | 19 | Provide names of organizations/persons that are acknowledged along with their contribution to the research. | Page 33, Acknowledgments |
