# Supplementary material for: Medication adherence and its associated factors among oral pre-exposure prophylaxis (PrEP) users in China: The Real-world E-consumer Cohort of PrEP study
Source: PLoS Med. 2026 Feb 26;23(2):e1004733. doi: 10.1371/journal.pmed.1004733 (PMC12944781; doi:10.1371/journal.pmed.1004733)
Supplement: Attachment — Submitted filename: plos-response letter-sl.docx [file pmed.1004733.s015.docx]

Dear Editors,

Thank you very much for your review of the manuscript *Medication adherence and its associated factors among oral PrEP users in China: Main results of the real-world e-consumer cohort of pre-exposure prophylaxis (RECOPE)* We have considered your specific comments carefully and provided a point-by-point response below. We believe the manuscript is much more informative and stronger now.

*********************************************************************

**Editor**

Comments to the Author

1.1 We ask that you provide a more detailed description of the methodology.

Response: Thank you for the suggestion. We have made the modifications accordingly, including provision of a more detailed description of the methodology (e.g., elaborated the study design and procedure, add a sample size calculation section)

(Methods, page 6-7)

***Study Design***

*The study established the first ever Real-world E-consumer Cohort of PrEP (RECOPE) in China. It was a prospective cohort of e-consumers of PrEP, designed to monitor PrEP adherence and explore associated risk factors. The participant recruitment and baseline assessment of the cohort were conducted in December 2023 to January 2024, and three follow-up assessments were completed at 1-, 3-, and 6-months. The study had obtained ethical approval from the Institutional Review Board of the corresponding author’s affiliated organization (reference number: 20230195).*

***Sample Size***

*The required minimum sample size was calculated using Cochran’s formula for estimating a single population proportion, incorporating finite population correction and a design effect coefficient [17], as shown in Equation (1). Based on previous studies [18], we assumed a prevalence of optimal PrEP adherence of 60%, a two-sided significance level of α = 0.05, and a desired precision (margin of error) of 5%. A design effect (deff) of 1.1 and a 15% anticipated loss to follow-up were also applied. The final minimum sample size was calculated to be 424 participants using the PASS software.*

*n = deff × [N p̂ q̂] / [(d² / 1.96²)(N – 1) + p̂ q̂]	(1)*

***Participant Recruitment***

*The participants were recruited through Bluedhealth, the major online platform for PrEP sales in China. The staff from Bluedhealth were asked to send an internal invitation message via Bluedhealth to their users who had purchased PrEP within the past one to three months. Users who expressed an interest in the study were initially screened for eligibility. The inclusion criteria were as follows: (1) being aged 18 years or older; (2) having purchased PrEP online and being qualified for PrEP use; (3) planning to use PrEP over the next six months; and (4) providing informed consent. The exclusion criteria included having communication barriers or reading disabilities.*

**References:**

*[17]	Cochran WG. Sampling techniques. John Wiley & Sons; 1977.*

*[18]	Wang H, Wang Z, Huang X, Chen Y, Wang H, Cui S, et al. Association of HIV Preexposure Prophylaxis Use With HIV Incidence Among Men Who Have Sex With Men in China: A Nonrandomized Controlled Trial. JAMA Network Open 2022;5:e2148782. https://doi.org/10.1001/jamanetworkopen.2021.48782.*

1.2 Discuss whether lack of adherence accounted for sex with primary partners (i.e. clarify the definition of adherence).

Response: Thanks. We have clarified the definition of adherence in terms of sex with primary partners in the revised manuscript.

(Methods, page 8):

*“Adherence to ED regimen was assessed by four items. First, the participants were asked whether they had engaged in sexual activity in the past one and three months, respectively (the 1-month follow-up survey only asked this question for the past one month). Those who answered “yes” were then asked if they had taken PrEP for each sexual encounter in the corresponding time period, including those with both primary (e.g., main or long-term partners) and non-primary (e.g., casual or commercial partners) sexual partners (1 = every time, 2 = sometimes, and 3 = never).”*

1.3 Cite related papers on telemedicine approaches for PrEP delivery and add additional information on the e-consumer model.

Response: Thank you for your suggestion. We have elaborated the background of telemedicine approaches and e-consumer model of PrEP and added the relevant literature in the revised manuscript.

(Introduction, page 5):

*“Recently, telemedicine approaches for PrEP delivery have been emerging in some countries (e.g., the United States, China, and Brazil) due to its nature of convenience and privacy protection, which is particularly accelerated by the COVID-19 pandemic [10,11]. In common e-consumer models of PrEP, users who are interested in using the online platform to access to PrEP are required to complete an online medical history form, then receive remote evaluation of eligibility by a professional provider following the PrEP guidelines. If approved, a certain amount of medications (e.g., 90-day supply for daily PrEP users) are mailed directly to the clients, along with renewal instructions [10].*

*In China, e-health commerce platforms are the primary channel for purchasing PrEP currently. According to China CDC, nearly 70% of real-world PrEP users purchase PrEP online. The largest e-commerce platform is Bluedhealth operated by Blued, the most popular MSM-oriented community-based organization in Asia [12]. Users who are willing to purchase PrEP through Bluedhealth will need to submit a prescription request form and screened by a doctor remotely for eligibility. However, the virtual nature of online venues makes providing professional medication instructions and counselling difficult, which poses distinct challenges for helping consumers keep optimal medication adherence.”*

**References:**

*[10] Hughes SD, Koester KA, Engesaeth E, Hawkins MV, Grant RM. Human Enough: A Qualitative Study of Client Experience With Internet-Based Access to Pre-exposure Prophylaxis. Journal of Medical Internet Research 2021;23:e22650. https://doi.org/10.2196/22650.*

*[11]	Hoagland B, Torres TS, Bezerra DRB, Geraldo K, Pimenta C, Veloso VG, et al. Telemedicine as a tool for PrEP delivery during the COVID-19 pandemic in a large HIV prevention service in Rio de Janeiro-Brazil. Braz J Infect Dis 2020;24:360-4. https://doi.org/10.1016/j.bjid.2020.05.004.*

*[12]Zhu Y, Tian M, Yin W, Zhao D, Liu X, Xue P, et al. The compliance of HIV pre-exposure prophylaxis uptake among MSM. Chinese Journal of AIDS & STD 2023;05*

1.4 Discuss further the potential bias associated with convenience sampling.

Response: Thank you for your suggestion. We have discussed more about the associated bias of convenience sampling.

(Limitation, page 24-25):

*First, the convenience sampling method used in the study (conveniently choosing Bluedhealth as the recruitment channel and conveniently recruited the participants from Bluedhealth PrEP clients) may cause limited generalization ability and selection bias in the study. On one hand, although Bluedhealth is the most widely used e-commerce platform for PrEP purchases in China, caution is warranted when generalizing these findings to all e-consumers of PrEP in China and other settings where online PrEP services are available. On the other hand, PrEP users from Bluedhealth who chose to participate in the study may differ from non-respondents. For example, those with higher engagement in HIV prevention or better adherence may have been more willing to participate, which could lead to an overestimation of PrEP adherence.*

*********************************************************************

We thank the Editor again for reviewing the manuscript. Please do not hesitate to contact us if you had further comments.
