# Supplementary material for: Medication adherence and its associated factors among oral pre-exposure prophylaxis (PrEP) users in China: The Real-world E-consumer Cohort of PrEP study
Source: PLoS Med. 2026 Feb 26;23(2):e1004733. doi: 10.1371/journal.pmed.1004733 (PMC12944781; doi:10.1371/journal.pmed.1004733)
Supplement: Attachment — Submitted filename: Response Letter-final.docx [file pmed.1004733.s016.docx]

Dear Editor and Reviewers,

Thank you very much for your review of the manuscript PMEDICINE-D-25-02683R1. We have considered and addressed your specific comments carefully and provided a point-by-point response below. We believe the manuscript is much more informative and stronger now.

*********************************************************************

**Editor**

E1.1. Many thanks for submitting your manuscript "Medication adherence and its associated factors among oral PrEP users in China: Main results of the real-world e-consumer cohort of pre-exposure prophylaxis (RECOPE)" (PMEDICINE-D-25-02683R1) to PLOS Medicine. The paper has been reviewed by subject experts and a statistician; their comments are included below and can also be accessed here:

As you will see, the reviewers find the work of interest but have raised numerous concerns that would need to be addressed to consider a revised manuscript. In particular, the reviewers find that additional information on the study design and conduct, the representativeness of the participants, the effects of the study design on adherence, and how the data were handled and the statistical tests reported, is required, among other issues. The reviewers further find that the conclusions need to be tempered and the discussion of study limitations expanded to address the concerns raised in review. After discussing the paper with the editorial team and an academic editor with relevant expertise, I'm pleased to invite you to revise the paper in response to the reviewers’ comments.

Response: We sincerely thank the editors and reviewers for the careful review and insightful comments. We have substantially revised the manuscript to address all the comments, including

1) adding more detailed information on the study design and conduct,
2) clarifying the representativeness of the participants,
3) discussing the potential effects of the study design on PrEP adherence,
4) providing additional details on data handling and statistical analyses
5) and tempering our conclusions and expanding the discussion of study limitations.

A detailed point-by-point response is provided below.

E1.2. We note that an author is an employee of BlueHealth. Please amend your funder statement accordingly, as well as your competing interests statement.

Response: Thanks. We have revised the funding statement and competing interests statement accordingly.

Financial Disclosure: *‘The study was supported by the National Natural Science Foundation of China (grant number: 82404326) and the Capital’s Funds for Health Improvement and Research (grant number: 2024-2G-4402), both awarded to Dr. Sitong Luo. The funders had no role in study design, data collection, statistical analysis, data interpretation, manuscript writing, or the decision to submit the manuscript for publication. One of the authors, Dr. Hui Xue, is employed by BluedHealth, the e-commerce platform for PrEP sales in China which assisted in the participant recruitment in the study. BluedHealth had no role in study design, data analysis and interpretation, manuscript writing, or the decision to submit the manuscript for publication.*

Competing Interests Statement: *One of the authors, Dr. Hui Xue is employed by BluedHealth, the e-commerce platform for PrEP sales in China which assisted in the participant recruitment in the study. BluedHealth had no role in study design, data analysis and interpretation, manuscript writing, or the decision to submit the manuscript for publication. The other authors declare that they have no competing interests.*

E1.3. Please include a protocol and statistical analysis plan, if the latter is available.

Response: Thanks. We have included and uploaded our study protocol as the S1 File, which include study background, study objectives, eligibility criteria, participant recruitment, follow-up procedures, questionnaire development, planned statistical analyses, and quality control.

The statistical analysis plan is included in the protocol and specifies the primary and secondary outcomes, analysis populations, model choices, covariates and their coding, handling of repeated measures and missing-data, planned subgroup and sensitivity analyses, and software with version.

E1.4. Please explain what is meant by the ‘main results’ of RECOPE.

Response: The ‘main results’ means this manuscript reported the findings of the main research question of the RECOPE study. The RECOPE cohort was established primarily to investigate PrEP adherence and its associated factors among real-world e-consumers of oral PrEP in China, and the results are reported in this manuscript.

In addition, the cohort also collected information on other research questions such as utilization of other HIV-related services (e.g., willingness to use long-acting injectable PrEP, HIV testing behavior) and their associated factors, and the findings of these secondary research questions are planned to be reported in other papers.

E1.5. Please include a completed STROBE checklist and update the manuscript accordingly.

Response: We have uploaded the completed STROBE checklist as a supplementary file (S1 checklist) and updated the manuscript accordingly.

E1.6. Please also include the exact dates of participant enrollment.

Response: Thanks. We have included the exact dates of participant enrollment in the revised Methods section.

(page 7, lines 121–122) *‘The participant recruitment and baseline survey of the cohort were conducted* *from December 6, 2023 to January 4, 2024, and three follow-up surveys were completed at 1-, 3-, and 6-months.’*

E1.7. Please include the survey questions as supplementary information.

Response: We have uploaded the study questionnaires as the S2 File.

E1.8. Please report your survey response rates according to AAPOR recommendations https://aapor.org/standards-and-ethics/best-practices/

Response: Thanks. In the revised Results section and Figure 1, we have reported the response rate for the baseline (96.6%), 1-mon (90.1%), 3-mon (82.6%), and 6-mon (80.1%) surveys, according to the AAPOR standard definitions. To be noted, the response rates for the follow-up surveys are identical to the follow-up rates reported in the original manuscript, because the reasons for non-response to the follow-up surveys were the same as the ones for loss to follow-up (participants could not be contacted).

(page 15, lines 302–313) *‘Of the 877 individuals invited, 680 were eligible for the study and sent the baseline questionnaire (146 not respond to the study invitation, 33 not interested for participation after further communication with the recruiters, 18 had no plan to use PrEP in the future six months), and 657 completed the baseline survey (23 did not respond to the questionnaire due to break-off) and were finally enrolled in the RECOPE cohort. According to the American Association for Public Opinion Research (AAPOR)’s standard definitions, the response rate (RR) of the baseline survey was 96.6% (657/680). At the 1-, 3-, and 6-month follow-ups, 90.1% (592/657), 82.6% (543/657), and 80.1% (526/657) of the participants responded to the questionnaire surveys and successfully followed-up, with 582, 539, and 520 being included in the analysis after excluding those failing the quality check of questionnaires (Fig. 1).* *The patterns of loss to follow-up in the study were shown in S1 Table.’*

**Fig 1. Flowchart of the RECOPE study.**

E1.9. Please define how the population surveyed was sampled.

Response: Thanks. We have elaborated the sampling procedure in the revised Methods section under Participant Recruitment. In short, a convenient sampling method was used to recruit clients who had bought PrEP in the past 1-3 months at the time of the recruitment from BluedHealth, the largest online platform of PrEP sales in China.

(page 7, lines 128–141) *‘The participants were conveniently sampled and recruited through BluedHealth, the major online platform for PrEP services in China established by Blued (BlueCity Group) in 2019. Blued, launched in 2012, is the most widely used gay geosocial networking application in China and Asia. It has over 70 million registered users and more than 7 million monthly active users, approximately half of whom are based in mainland China. BluedHealth is embedded within the Blued app and provides user-friendly, accessible, and confidential HIV prevention and care services. This e-commerce platform offers sales of HIV preventive products (e.g., PrEP, post-exposure prophylaxis, condoms, HIV/syphilis/hepatitis B virus [HBV] self-test kits) and related logistics and delivery. It also provides online health education and counselling. The average number of PrEP purchases per month on BluedHealth is over 4,000 [13]. During the study period, the price of PrEP on BluedHealth was approximately 266-2280 CNY (36.7-314.5 USD) per 30-tablet bottle, varied by manufacturers, which is comparable to the in-store price in China. ’*

E1.10. Please compare characteristics of respondents and nonrespondents if possible.

Response: We thank the Editor for the suggestion. We agree that comparing characteristics between respondents and nonrespondents is very informative for understanding the findings and limitations of the study. However, as we did not collect any data from those who did not respond to the study invitation or respond to the baseline survey, we are unable to compare the characteristics between the respondents and non-respondents of the study recruitment/baseline survey, which makes it difficult to gauge the direction and extent of selection bias brought by the convenience sampling method of the study. In the revised Limitation section, we have clarified this point and acknowledged it as a limitation.

(page 29, lines 502–509) *‘First, the convenience sampling method used in the study might introduce selection bias. PrEP users from BluedHealth who were interested in the study and successfully submitted the baseline questionnaire might differ from the potentially eligible non-respondents to the study invitation/baseline survey. As information on these non-respondents were unavailable, it was difficult to gauge the direction and extent of the selection bias. It is possible that those with better PrEP adherence might be more willing to participate in the study, which could lead to an overestimation of PrEP adherence.’*

In addition, we have data of the non-respondents in the follow-up surveys and are able to examine whether the characteristics of the respondents change over time or remain constant (please refer to the response to the next comment E1.11).

E1.11. Were sequential waves of the survey sent? If yes, did the characteristics of respondents change over time or remain constant?

Response: Yes, sequential waves of survey questionnaire were sent for follow-up in the study and we are able to compare the characteristics of respondents across different waves to see whether they were changing over time or remain constant.

1) The RECOPE is a fixed cohort. We recruited the participants from December 6, 2023 to January 4, 2024, and those who successfully submitted their baseline survey questionnaire were finally included in the cohort and followed-up by sending sequential waves of questionnaires at 1-, 3-, and 6-months.
2) As mentioned in the response to comment E1.8, the follow-up rate at 1-mon (90.1%), 3-mon (82.6%), and 6-mon (80.1%) follow-ups are identical to the response rate for the follow-up survey at each wave. In the revised manuscript, we further added a supplementary S1 Table to show the patterns of loss to follow-up/survey non-respondents, and found that 27.5% of the cohort participants had at least one loss to follow-up/non-response to the follow-up surveys.

S1 Table : Patterns of loss to follow-up of the study

| **Patterns** | **n** | **% (n/657)** |
| --- | --- | --- |
| Lost to follow-up (survey non-responses) for three times | 47 | 7.1% |
| Lost to follow-up (survey non-responses) for two times | 56 | 8.5% |
| Lost to follow-up (survey non-responses) for one time | 78 | 11.9% |
| Total | 181 | 27.5% |

3) In the revised manuscript, we compared the socio-demographic and other characteristics across the four study waves. In the revised Table 1, we can see that all the socio-demographic characteristics of the respondents remain constant across different waves (p-value for comparing the distribution of each variable cross different wave were all >0.05). And as shown in the revised Table 2, some psycho-behavior characteristics were significantly different across different waves (e.g., sexual behavior, PrEP knowledge, psychological resilience), which may be largely due to the within-person variance across different time points. We also modified the Methods and Results section accordingly.

(page 13, lines 266–271): *‘First, descriptive analyses were performed to summarize the distributions of all the socio-demographic and psycho-behavioral variables across the study waves, both for the overall sample and sub-samples by regimens. The Chi-square test and t test were conducted to examine differences in the variable distributions between study waves. The prevalence of optimal PrEP adherence at each wave was calculated for ED and daily users separately.*’

(page 16, lines 315–326): *‘The distributions of socio-demographics in total and by regimens across different survey waves were presented in Table 1. For the total sample at baseline, the mean age of the study participants was 29.9 years (standard deviation [SD] = 6.4). All the participants were assigned male sex at birth and reported to be men who have sex with men (MSM). Most participants were Han ethnicity people (n = 615, 93.6%), employed (n = 527, 80.2%), having a college education or above (n = 624, 95.0%), unmarried (n = 567, 86.3%), and reporting a monthly income over 7000 CNY (n = 385, 58.6%). Of the 621 (94.5%) participants who have already initiated PrEP at baseline, 529 (85.2%) used the ED regimen and 92 (14.8%) took daily regimen. The distributions of socio-demographics in total and by regimens remained constant throughout the survey waves (all p>0.05). The socio-demographic characteristics of those who had not initiated PrEP reported in each wave were shown in S2 Table.*

(page 19, lines 331–345): ‘*Table 2 showed the distributions of sexual behaviors, PrEP-related variables, and psychosocial variables in total and by regimen types at each survey wave. In general, the majority of the total sample (78.4-90.4%) reported recent sexual activities across all survey waves. Prevalence of high-risk sexual behaviors in the total sample were 38.9%-42.7% for chemsex, 37.7-43.8% inconsistent condom use, 69.4-81.0% for having multiple sexual partners, and 7.3-9.4% for commercial sex. About 44.3-48.5% of the total sample reported their sexual role as receptive or versatile. The majority of the participants (69.3%-76.2%) demonstrated correct knowledge of PrEP medication. The mean score of the self-efficacy scale ranged 3.9-4.4. About 63.1-70.2% reported mild to severe depressive symptoms. The mean score of the resilience scale ranged 5.2-5.8. The Chi-square/t test detected a significant difference (p<0.05) in sexual activity status, number of sexual partners, PrEP knowledge, self-efficacy for PrEP adherence, and resilience scores across survey waves in the total sample and ED users. Among daily users, significant differences across waves were observed for self-efficacy for PrEP adherence, PrEP-related stigma, and resilience’*

**Table 1.** Distributions of socio-demographic characteristics in the RECOPE study.

| **Variables** | **Total** | | | | ***P ^a^*** |  | **ED regimen** | | | | ***P ^a^*** |  | **Daily regimen** | | | | ***P ^a^*** |
| --- | --- | --- | --- | --- | --- | --- | --- | --- | --- | --- | --- | --- | --- | --- | --- | --- | --- |
|  | **Baseline**  **(N=657)** | **1 month**  **(N=582)** | **3 month**  **(N=539)** | **6 month**  **(N=520)** |  |  | **Baseline**  **(n=529)** | **1 month**  **(n=458)** | **3 month**  **(n=428)** | **6 month**  **(n=422)** |  |  | **Baseline**  **(n=92)** | **1 month**  **(n=102)** | **3 month**  **(n=98)** | **6 month**  **(n=88)** |  |
| **Age (years, Mean±SD)** | 29.9±6.4 | 29.8±6.5 | 29.8±6.4 | 29.9±6.4 | 0.976 |  | 29.9±6.4 | 29.6±6.6 | 29.7 ±6.4 | 29.72 ± 6.5 | 0.938 |  | 30.4±6.4 | 31.00± 6.1 | 30.17 ±6.0 | 31.14 ±6.1 | 0.655 |
| 18-29 | 359 (54.6) | 322 (55.3) | 297 (55.1) | 278 (53.4) |  |  | 289 (54.6) | 260 (56.8) | 240 (56.1) | 235 (55.7) |  |  | 48 (52.2) | 48 (47.1) | 50 (51.0) | 38 (43.2) |  |
| 30-39 | 246 (37.5) | 214 (36.8) | 202 (37.5) | 203 (39.1) |  |  | 198 (37.5) | 164 (35.8) | 154 (36.0) | 155 (36.8) |  |  | 37 (40.2) | 47 (46.1) | 43 (43.9) | 43 (48.9) |  |
| >40 | 52 (7.9) | 46 (7.9) | 40 (7.4) | 39 (7.5) |  |  | 42 (7.9) | 34 (7.4) | 34 (7.9) | 32 (7.6) |  |  | 7 (7.6) | 7 (6.9) | 5 (5.1) | 7 (8.0) |  |
| **Designated Sex at Birth** |  |  |  |  | N/A |  |  |  |  |  | N/A |  |  |  |  |  | N/A |
| Male | 657 (100.0) | 582 (100.0) | 539 (100.0) | 520 (100.0) |  |  | 529 (100.0) | 458 (100.0) | 428 (100.0) | 422 (100.0) |  |  | 92 (100.0) | 102 (100.0) | 98 (100.0) | 88 (100.0) |  |
| Female | 0 (0.0) | 0 (0.0) | 0 (0.0) | 0 (0.0) |  |  | 0 (0.0) | 0 (0.0) | 0 (0.0) | 0 (0.0) |  |  | 0 (0.0) | 0 (0.0) | 0 (0.0) | 0 (0.0) |  |
| **Ethnicity** |  |  |  |  | 0.963 |  |  |  |  |  | 0.977 |  |  |  |  |  | 0.965 |
| Han | 615 (93.6) | 547 (94.0) | 508 (94.2) | 491 (94.3) |  |  | 497 (94.0) | 432 (94.3) | 405 (94.6) | 398 (94.3) |  |  | 85 (92.4) | 94 (92.2) | 92 (93.9) | 82 (93.2) |  |
| Others | 42 (6.4) | 35 (6.0) | 31 (5.8) | 29 (5.7) |  |  | 32 (6.0) | 26 (5.7) | 23 (5.4) | 24 (5.7) |  |  | 7 (7.6) | 8 (7.8) | 6 (6.1) | 6 (6.8) |  |
| **Employment** |  |  |  |  | 0.978 |  |  |  |  |  | 0.926 |  |  |  |  |  | 0.540 |
| Students/Unemployed | 130 (19.8) | 119 (20.4) | 112 (20.8) | 106 (20.3) |  |  | 114 (21.6) | 105 (22.9) | 96 (22.4) | 96 (22.4) |  |  | 9 (9.8) | 10 (9.8) | 13 (13.3) | 6 (6.8) |  |
| Employed | 527 (80.2) | 463 (79.6) | 427 (79.2) | 414 (79.7) |  |  | 415 (78.4) | 353 (77.1) | 332 (77.6) | 333 (77.6) |  |  | 83 (90.2) | 92 (90.2) | 85 (86.7) | 82 (93.2) |  |
| **Education Level** |  |  |  |  | 0.999 |  |  |  |  |  | 1.000 |  |  |  |  |  | 0.952 |
| Junior high school or below | 4 (0.6) | 2 (0.3) | 3 (0.6) | 3 (0.6) |  |  | 4 (0.8) | 2 (0.4) | 3 (0.7) | 3 (0.7) |  |  | 0 (0.0) | 0 (0.0) | 0 (0.0) | 0 (0.0) |  |
| Senior high school / Vocational high school / Technical secondary school | 29 (4.4) | 24 (4.1) | 22 (4.1) | 25 (48) |  |  | 23 (4.3) | 18 (3.9) | 17 (4.0) | 18 (4.3) |  |  | 6 (6.5) | 6 (5.9) | 5 (5.1) | 7 (8.0) |  |
| College diploma (Associate degree) / Bachelor’s degree | 436 (66.4) | 384 (66.0) | 358 (66.4) | 343 (65.9) |  |  | 353 (66.7) | 309 (67.5) | 287 (67.1) | 282 (66.8) |  |  | 54 (58.7) | 58 (56.9) | 61 (62.2) | 54 (61.4) |  |
| Master’s degree or above | 188 (28.6) | 172 (29.6) | 156 (28.9) | 149 (28.7) |  |  | 149 (28.2) | 129 (28.2) | 121 (28.3) | 119 (28.2) |  |  | 32 (34.8) | 38 (37.3) | 32 (32.7) | 27 (30.7) |  |
| **Marital Status** |  |  |  |  | 0.646 |  |  |  |  |  | 0.524 |  |  |  |  |  | 0.898 |
| Married or living with a partner | 90 (13.7) | 82 (14.1) | 81 (15.0) | 84 (16.2) |  |  | 77 (14.6) | 63 (13.8) | 68 (15.9) | 72 (17.1) |  |  | 11 (12.0) | 16 (15.7) | 13 (13.3) | 12 (13.6) |  |
| Unmarried/Divorced/Separated/Widowed | 567 (86.3) | 500 (85.9) | 458 (85.0) | 436 (83.8) |  |  | 452 (85.4) | 395 (86.2) | 360 (84.1) | 350 (82.9) |  |  | 81 (88.0) | 86 (84.3) | 85 (86.7) | 76 (86.4) |  |
| **Monthly income (CNY) ^b^** |  |  |  |  | 1.000 |  |  |  |  |  | 1.000 |  |  |  |  |  | 0.961 |
| ≤3000 | 77 (11.7) | 72 (12.4) | 67 (12.4) | 61 (11.8) |  |  | 62 (11.7) | 61 (13.3) | 55 (12.9) | 54 (12.8) |  |  | 9 (9.8) | 6 (5.9) | 9 (9.2) | 5 (5.7) |  |
| 3001-5000 | 92 (14.0) | 82 (14.1) | 77 (14.3) | 75 (14.5) |  |  | 78 (14.7) | 70 (15.3) | 64 (15.0) | 67 (15.9) |  |  | 9 (9.8) | 11 (10.8) | 13 (13.3) | 8 (9.1) |  |
| 5001-7000 | 103 (15.7) | 87 (14.9) | 82 (15.2) | 78 (15.0) |  |  | 91 (17.2) | 73 (15.9) | 71 (16.6) | 68 (16.1) |  |  | 9 (9.8) | 12 (11.8) | 11 (11.2) | 10 (11.4) |  |
| 7001-10000 | 121 (18.4) | 114 (19.6) | 107 (19.9) | 105 (20.2) |  |  | 95 (18.0) | 84 (18.3) | 77 (18.0) | 82 (19.4) |  |  | 18 (19.6) | 23 (22.5) | 25 (25.5) | 20 (22.7) |  |
| 10001-15000 | 127 (19.3) | 110 (18.9) | 104 (19.3) | 104 (19.8) |  |  | 101 (19.1) | 89 (19.4) | 81 (18.9) | 80 (18.9) |  |  | 19 (20.7) | 19 (18.6) | 21 (21.4) | 22 (25.0) |  |
| 15001-20000 | 57 (8.7) | 48 (8.2) | 42 (7.8) | 35 (6.7) |  |  | 38 (7.2) | 33 (7.2) | 29 (6.8) | 21 (5.0) |  |  | 15 (16.3) | 13 (12.7) | 11 (11.2) | 12 (13.6) |  |
| >20000 | 80 (12.2) | 69 (11.9) | 60 (11.1) | 62 (11.9) |  |  | 64 (12.1) | 48 (10.5) | 51 (11.9) | 50 (11.9) |  |  | 13 (14.1) | 18 (17.6) | 8 (8.2) | 11 (12.5) |  |

^a^ The Chi-square test was used for categorical variables and t-test was used for continuous variables.

^b^ CNY, Chinese Yuan (1 CNY=0.14 USD)

**Table 2.** Distribution of sexual behaviors, PrEP-related variables, and psychosocial variables in the RECOPE study

| **Variables** | **Total** | | | | ***P ^d^*** |  | **ED regimen** | | | | ***P ^d^*** |  | **Daily regimen** | | | | ***P ^d^*** |  | **Continuous Sexually active^a^** |
| --- | --- | --- | --- | --- | --- | --- | --- | --- | --- | --- | --- | --- | --- | --- | --- | --- | --- | --- | --- |
|  | **Baseline**  **(N=657)** | **1 month**  **(N=582)** | **3 month**  **(N=539)** | **6 month**  **(N=520)** |  |  | **Baseline**  **(n=529)** | **1 month**  **(n=458)** | **3 month**  **(n=428)** | **6 month**  **(n=422)** |  |  | **Baseline**  **(n=92)** | **1 month**  **(n=102)** | **3 month**  **(n=98)** | **6 month**  **(n=88)** |  |  |  |
| **Sexually active** |  |  |  |  | **<0.001** |  |  |  |  |  | **<0.001** |  |  |  |  |  | 0.476 |  |  |
| Yes | 578 (88.0) | 454 (78.4) | 481 (89.2) | 470 (90.4) |  |  | 458 (86.6) | 339 (74.0) | 377 (88.1) | 374 (88.8) |  |  | 88 (95.7) | 100 (98.0) | 94 (95.9) | 88 (98.9) |  |  | 310 (100.0) |
| No | 79 (12.0) | 128 (21.6) | 58 (10.8) | 50 (9.6) |  |  | 71 (13.4) | 119 (26.0) | 51 (11.9) | 47 (11.2) |  |  | 4 (4.3) | 2 (2.0) | 4 (4.1) | 1 (1.1) |  |  | 0 (0.0) |
| **Chemsex^b^** |  |  |  |  | 0.564 |  |  |  |  |  | 0.338 |  |  |  |  |  | 0.803 |  |  |
| Yes | 247 (42.7) | 182 (40.1) | 202 (42.0) | 183 (38.9) |  |  | 202 (44.1) | 134 (39.5) | 157 (41.6) | 143 (38.2) |  |  | 35 (39.8) | 44 (44.0) | 44 (46.8) | 37 (42.0) |  |  | 160 (51.6) |
| No | 331 (57.3) | 272 (59.9) | 279 (58.0) | 287 (61.1) |  |  | 256 (55.9) | 205 (60.5) | 220 (58.4) | 231 (61.8) |  |  | 53 (60.2) | 56 (56.0) | 50 (53.2) | 51 (58.0) |  |  | 150 (48.4) |
| **Condom use^b^** |  |  |  |  | 0.090 |  |  |  |  |  | 0.303 |  |  |  |  |  | 0.790 |  |  |
| Inconsistent | 218 (37.7) | 201 (44.3) | 210 (43.7) | 206 (43.8) |  |  | 164 (35.8) | 141 (41.6) | 154 (40.8) | 151 (40.4) |  |  | 46 (52.3) | 56 (56.0) | 55 (58.5) | 52 (59.1) |  |  | 144 (46.5) |
| Consistent | 360 (62.3) | 253 (55.7) | 271 (56.3) | 264 (56.2) |  |  | 294 (64.2) | 198 (58.4) | 223 (59.2) | 223 (59.6) |  |  | 42 (47.7) | 44 (44.0) | 39 (41.5) | 36 (40.9) |  |  | 166 (53.5) |
| **Sexual partner^b^** |  |  |  |  | **<0.001** |  |  |  |  |  | **<0.001** |  |  |  |  |  | 0.240 |  |  |
| 1 | 110 (19.0) | 139 (30.6) | 125 (26.0) | 132 (28.1) |  |  | 84 (18.3) | 108 (31.9) | 109 (28.9) | 115 (30.7) |  |  | 17 (19.3) | 25 (25.0) | 13 (13.8) | 15 (17.0) |  |  | 53 (17.1) |
| >1 | 468 (81.0) | 315 (69.4) | 356 (74.0) | 338 (71.9) |  |  | 374 (81.7) | 231 (68.1) | 268 (71.1) | 259 (69.3) |  |  | 71 (80.7) | 75 (75.0) | 81 (86.2) | 73 (83.0) |  |  | 257 (82.9) |
| **Sexual role^b^** |  |  |  |  | 0.649 |  |  |  |  |  | 0.650 |  |  |  |  |  | 0.600 |  |  |
| Receptive or versatile | 267 (46.2) | 220 (48.5) | 222 (46.2) | 208 (44.3) |  |  | 222 (48.5) | 172 (50.7) | 176 (46.7) | 174 (46.5) |  |  | 34 (38.6) | 44 (44.0) | 44 (46.8) | 34 (38.6) |  |  | 155 (50.0) |
| Insertive | 311 (53.8) | 234 (51.5) | 259 (53.8) | 262 (55.7) |  |  | 236 (51.5) | 167 (49.3) | 201 (53.3) | 200 (53.5) |  |  | 54 (61.4) | 56 (56.0) | 50 (53.2) | 54 (61.4) |  |  | 155 (50.0) |
| **Commercial sex^b^** |  |  |  |  | 0.676 |  |  |  |  |  | 0.673 |  |  |  |  |  | 0.892 |  |  |
| Yes | 50 (8.7) | 36 (7.9) | 35 (7.3) | 44 (9.4) |  |  | 39 (8.5) | 27 (8.0) | 24 (6.4) | 31 (8.3) |  |  | 9 (10.2) | 9 (9.0) | 10 (10.6) | 11 (12.5) |  |  | 34 (11.0) |
| No | 528 (91.3) | 418 (92.1) | 446 (92.7) | 426 (90.6) |  |  | 419 (91.5) | 312 (92.0) | 353 (93.6) | 343 (91.7) |  |  | 79 (89.8) | 91 (91.0) | 84 (89.4) | 77 (87.5) |  |  | 276 (89.0) |
| **PrEP knowledge**^c^ |  |  |  |  | **0.014** |  |  |  |  |  | **0.002** |  |  |  |  |  | 0.590 |  |  |
| Correct | 455 (69.3) | 416 (71.5) | 410 (76.1) | 396 (76.2) |  |  | 390 (73.7) | 356 (77.7) | 356 (83.2) | 341 (81.0) |  |  | 42 (45.7) | 49 (48.0) | 46 (46.9) | 49 (55.1) |  |  | 233 (75.2) |
| In correct | 202 (30.7) | 166 (28.5) | 129 (23.9) | 124 (23.8) |  |  | 139 (26.3) | 102 (22.3) | 72 (16.8) | 80 (19.0) |  |  | 50 (54.3) | 53 (52.0) | 52 (53.1) | 40 (44.9) |  |  | 77 (24.8) |
| **Self-efficacy of PrEP adherence** | 3.9±1.8 | 4.2±1.8 | 4.4±1.8 | 4.3±1.9 | **<0.001** |  | 3.8±1.9 | 4.0±1.8 | 4.3±1.8 | 4.1±1.9 | **<0.001** |  | 4.6±1.6 | 5.1±1.4 | 5.1±1.3 | 5.1±1.6 | **0.041** |  | 4.1±1.7 |
| **PrEP-related stigma** | 25.1±6.9 | - | 25.0±7.1 | 24.9±6.6 | 0.084 |  | 25.3±6.7 | - | 25.4±6.9 | 24.7±6.7 | 0.275 |  | 23.2±7.3 | - | 23.3±7.4 | 25.7±6.6 | **0.011** |  | 25.1±4.6 |
| **Depressive symptoms** | 7.2±5.3 | - | 7.4±5.3 | 7.2±5.2 | 0.267 |  | 7.1±5.3 | - | 7.3±5.3 | 7.0±5.0 | 0.283 |  | 7.4±5.4 | - | 7.5±5.7 | 8.0±5.7 | 0.477 |  | 7.2±3.7 |
| None | 215 (32.7) | - | 172 (31.9) | 155 (29.8) |  |  | 175 (33.1) | - | 135 (25.3) | 129 (30.6) |  |  | 29 (31.5) | - | 30 (32.6) | 23 (25.8) |  |  | 65 (21.0) |
| Mild | 280 (42.6) | - | 233 (43.2) | 249 (47.9) |  |  | 223 (42.2) | - | 196 (37.1) | 207 (49.2) |  |  | 40 (43.5) | - | 27 (29.3) | 40 (44.9) |  |  | 103 (49.4) |
| Moderate | 97 (14.8) | - | 73 (13.5) | 67 (12.9) |  |  | 79 (14.9) | - | 61 (11.5) | 50 (11.9) |  |  | 13 (14.1) | - | 9 (9.8) | 14 (15.7) |  |  | 56 (18.1) |
| Moderate severe | 40 (6.1) | - | 43 (8.0) | 34 (6.5) |  |  | 32 (6.0) | - | 36 (6.8) | 25 (5.9) |  |  | 6 (6.5) | - | 5 (5.4) | 8 (9.0) |  |  | 20 (6.5) |
| Severe | 25 (3.8) | - | 18 (3.3) | 15 (2.9) |  |  | 20 (3.8) | - | 16 (30) | 10 (2.4) |  |  | 4 (4.3) | - | 2 (2.2) | 4 (4.5) |  |  | 16 (5.2) |
| **Resilience** | 5.2±1.9 | - | 5.7±1.7 | 5.8±1.6 | **<0.001** |  | 5.2±1.9 | - | 5.7±1.7 | 5.7±1.7 | **<0.001** |  | 5.0±2.1 | - | 5.7±1.7 | 6.0±1.6 | **<0.001** |  | 5.7±1.2 |

^a^ Chemsex, and commercial sex were classified as “yes” if participants reported engaging in these behaviors at least once across the two follow-up assessments. If participants reported Having multiple homosexual partners at least once during the two follow-ups, they were classified as “> 1”. If participants reported Inconsistent condom use at least once during the two follow-ups, they were classified as “Inconsistent”. If participants reported their sexual role as receptive or versatile at least once during the two follow-ups, they were classified as “receptive or versatile”. Self-efficacy for being adhere to PrEP, resilience, depressive symptoms, and PrEP-related stigma in the last column were assessed using the mean scores from the two follow-up assessments.

^b^ For chemsex, condom use, sexual partner, sexual role, and commercial sex, the total sample for these variables are those who have reported being sexually active for the past study duration.

^c^ PrEP-related knowledge was assessed by asking participants to identify correct dosing instructions based on their regimen (daily or ED). Only those who selected all correct options were coded as having correct PrEP adherence knowledge.

^d^ The Chi-square test was used for categorical variables and t-test was used for continuous variables.

E1.12. Please include the survey response rate in your Abstract.

Response: We have included it accordingly.

(page 2, lines 34–36) ‘Of *877 individuals invited, 680 were eligible and 657 completed the baseline survey (response rate 96.6%). The follow-up rate/response rate of the 1-, 3-, and 6-months visit was 90.1%, 82.6%, and 80.1%, respectively.*

*********************************************************************

**Academic editor**

E2.1 The issue of representativeness of the sample. The current draft does discuss this in the limitations section but I would argue that it should have its own section or paragraph in the Discussion. The reviewers point out sampling concerns (e.g. highly educated Han participants only) beyond just the convenience sampling that could also be considered within the context of China’s overall population, since most readers will not be able to judge how generalizable this study is without being informed.

Response: Thanks for the comment. In the revised Limitation part in the Discussion section, we have added a separate paragraph discussing the representativeness of the sample and included related references [12,47-49] . In short, some characteristics of the study sample were different with previous PrEP-related study in China (e.g., education and income levels were higher in the study), while some were consistent to previous PrEP-related study or the overall Chinese population (e.g., 91.11 % of the total population in China are Han Chinese)

(page 30-31, lines 535-551) *‘Furthermore, the convenience sampling from a single e-commerce platform may also lead to limited representativeness of the study sample. Caution is warranted when generalizing the study findings to all e-consumers of PrEP in China. For example, all the participants in the study were MSM. Although it is true that MSM account for the majority of PrEP users in the country, those with a heterosexual orientation were completely not captured in the study. In addition, 95% of the participants had an education level of college or above, higher than that in previous PrEP studies in China (e.g., 70.0%-84.1% received college or above education) [12,47,48]. The study also reported a higher monthly income than previous studies among Chinese MSM [12,47,48]. Some other characteristics of the study sample were comparable to previous PrEP-related studies in China or the context of overall Chinese population. For example, 93.6 % of the study participants were Han people. According to the latest National Population Census in China, Han ethnicity accounted for 91.11 % of the total population [49]. Last but not least, the study only included people who bought PrEP online. However, other PrEP delivery systems such as hospitals, clinics, and CDC are also important venues for promoting PrEP use. Future research on these dispensing venues will be needed.’*

**References:**

[12] Zhu Y, Tian M, Yin W, et al. The compliance of HIV pre-exposure prophylaxis uptake among MSM. Chinese Journal of AIDS & STD 2023;05. doi: 10.13419/j.cnki.aids.2023.05.11 [In Chinese]

<https://www.stats.gov.cn/english/PressRelease/202105/t20210510_1817185.html>

[47] Zhang, G., Yang, X., Kang, W. et al. Pre-exposure prophylaxis uptake for high-risk men who have sex with men in China: a multi-city cross-sectional survey. AIDS Res Ther. 2023;32(20). [doi: 10.1186/s12981-023-00528-w](https://doi.org/10.1186/s12981-023-00528-w)

[48] Zhang J, Xu JJ, Wang HY, et al. Preference for daily versus on-demand pre-exposure prophylaxis for HIV and correlates among men who have sex with men: the China Real-world Oral PrEP Demonstration study. J Int AIDS Soc. 2021;24(2). doi:10.1002/jia2.25667

[49] National Bureau of Statistics of China. Main Data of the Seventh National Population Census (2021).

E2.2. The point made by one of the reviewers about whether trial follow-up affected outcomes. It is always a key concern for trials with behavior (adherence or retention) as an endpoint. This should be addressed.

Response: Thanks for the comment.

1） We acknowledge that trial or cohort follow-up with behavior may affect levels of psycho-behavior variables such as medication adherence and related knowledge and perceptions. It may have an interventional effect on behaviors as the follow-up itself may play as a reminder (cue to action) for performing the behavior.
2） For example, in the study, the adherence outcome (e.g., from 41.3% to 52.6% among ED users), PrEP knowledge (from 69.3% to 76.2% among all participants), and self-efficacy of being adhere (from 3.9 to 4.3 among all participants) showed an increase to some extent from baseline to subsequent follow-ups. This may be because the follow-up itself might have reminded the participants to keep optimal adherence and to pay more attention to acquire PrEP knowledge, as well as increased their self-efficacy (interventional effect).
3） We have incorporated related discussion in the revised Limitation section.

(page 30, lines 518-524). *‘Third, the follow-up itself may have an interventional effect on psycho-behavior variables. For example, the adherence outcome, PrEP knowledge, and self-efficacy of being adhere showed an increase to some extent from baseline to subsequent follow-ups in the study. This may be because the follow-up itself might have reminded the participants to keep optimal adherence and to pay more attention to acquire PrEP knowledge, as well as increased their self-efficacy. This follow-up effect should be considered when interpreting the results.’*

*********************************************************************

**Reviewer #1**

This is an interesting and timely study that evaluates PrEP adherence delivered through an “e-commerce” platform. I have reviewed Revision 1, in which the authors addressed some prior reviewer comments. However, several areas still require clarification, particularly in the Methods and Analysis sections.

Response: We sincerely thank the reviewer for all the comments. A detailed point-by-point response to each specific comment is provided below.

**Methodology - Additional detail needed**

R1.1 While telemedicine is a common approach for PrEP delivery, its implementation varies widely. Providing more detail about BluedHealth and its telehealth model would strengthen the manuscript. General Information about BluedHealth: Please include:

- The format of the service (app or website)
- Year of establishment
- Other services offered (beyond PrEP and PEP, if applicable)
- Approximate user base or annual client volume

If this information is available in a prior publication, please cite it. I attempted to review reference 12, but it was not available in English.

Response: Thanks. We have revised the Methods section to add a detailed description about BluedHealth, including its telehealth model, format, year of establishment, other services, approximate user base, and an English-language citation [13].

(page 7, lines 128–138) ‘*The participants were conveniently sampled and recruited through BluedHealth, the major online platform for PrEP services in China established by Blued (BlueCity Group) in 2019. Blued, launched in 2012, is the most widely used gay geosocial networking application in China and Asia. It has over 70 million registered users and more than 7 million monthly active users, approximately half of whom are based in mainland China. BluedHealth is embedded within the Blued app and provides user-friendly, accessible, and confidential HIV prevention and care services. This e-commerce platform offers sales of HIV preventive products (e.g., PrEP, post-exposure prophylaxis, condoms, HIV/syphilis/hepatitis B virus [HBV] self-test kits) and related logistics and delivery. It also provides online health education and counselling. The average number of PrEP purchases per month on BluedHealth is over 4,000* *[13]……’*

**Reference:**

[13] Shan D, Xue H, Yu F, Zan X, Liu H, Liu J, Han M, Zhang D. Understanding the uptake and outcomes of non-occupational postexposure prophylaxis use through an online medical platform in China: web-based cross-sectional study. Journal of Medical Internet Research. 2023 May 19;25:e42729.

R1.2 Initial Telehealth Encounter and Follow-Up:

Lines 88-91 raise concerns about the telehealth approach: "However, the virtual nature of online ventures makes providing professional medication instructions and counseling difficult, which poses distinct challenges for helping consumers maintain optimal medication adherence."

Please elaborate on this point. Why would counseling differ from in-person visits, given that it does not require physical contact? If this was a concern from the outset, the study design should have included measures to evaluate this hypothesis. Additionally, none of the adherence barrier questions appear to address this issue.

Response: Thanks.

1) In the revised Introduction section, we have elaborated on the flow of purchasing PrEP online and why would online counseling differ from in-person visits.

(page 5, lines 89–107) *‘……**Despite its advantages in convenience and privacy protection, the current online services of PrEP present some disadvantages in providing comprehensive instructions and counseling on PrEP use, which may pose distinct challenges for maintaining optimal medication adherence. For instance, when people are willing to purchase PrEP through BluedHealth, the platform would first require them to complete an online screening questionnaire for HIV exposure risk and PrEP eligibility (e.g., recent HIV testing result, contraindications to PrEP. Then a doctor would review the screening questions remotely and issue an electronic prescription and provide a standard written instructions on PrEP use if the clients met PrEP eligibility. No other interactive counselling would be provided at this stage. If a client had questions, he/she needs to join another online counselling group on BluedHealth for further communication. In contrast, during offline clinic visits for PrEP acquisition, in-person, face-to-face, and one-on-one counselling can be directly provided by doctors during and after the visit. It allows for immediate feedback and counseling adjustments, more nuanced history-taking of PrEP users, and direct observation of nonverbal cues. Therefore, compared with offline counselling, online counselling has a poorer accessibility, more complex procedure, and less flexibility of interacting with doctors, which may negatively affect clients’ understanding about PrEP use and ultimately reduce medication adherence.’*

2) We acknowledged that the study design did not include direct measures to evaluate this hypothesis (e.g., a question asking people actual utilization/reception of online counselling and their perceived quality or satisfaction) and did not include an adherence barrier question directly asking whether limited counseling affected adherence. However, the results regarding PrEP knowledge and adherence barriers in the study may infer that the participants might not have received enough counselling on PrEP use. For example, among the event-driven users, approximately 30% did not correctly answered the PrEP knowledge questions, and the top reasons for non-adherence included “I used other HIV prevention” and “I trusted my partner.” More in-depth counseling might help correct these misunderstanding and wrong perceptions. We have now acknowledged the lack of direct measures as a limitation in the revised Discussion section.

(page 30, lines 525–531) *‘Fourth, the study assumed that the current online services of PrEP make it difficult for PrEP users to receive comprehensive instructions and professional counseling, which may pose distinct challenges for helping them maintain optimal medication adherence. However, the study did not include measurement which directly evaluate the participants’ actual reception of online counselling and their perceived quality and satisfaction of the counselling, as well as their perceived influence or importance of counseling on adherence. Future studies are suggested to address this issue.’*

R1.3 Please describe the cost of PrEP in China and through this platform. Is it more affordable than conventional options?

Response: Thanks. We have revised the Methods section to describe the cost of PrEP on BluedHealth. During our study period, the price of PrEP on BluedHealth varied by manufacturer from approximately 266 to 2280 CNY (36.7-314.5 USD) per 30-tablet bottle, which is consistent with offline pricing.

(page 7, lines 138–141) *‘……During the study period, the price of PrEP on BluedHealth was approximately 266-2280 CNY (36.7-314.5 USD) per 30-tablet bottle, varied by manufacturers, which is comparable to the in-store price in China.’*

R1.4 The incentive method could influence follow-up rates. Please explain the BluedHealth voucher in more detail. What can it be used for?

Response: We have revised the Methods section to provide a clearer description of the incentive.

(page 9, lines 172–176) *‘**A BluedHealth voucher valued 50 CNY (approximately 7 USD) was provided upon completion of each valid survey. This voucher could be redeemed on BluedHealth for HIV prevention products and services, such as PrEP medications, condoms, and HIV/syphilis/HBV testing kits.’*

R1.5 It appeared that the participants were contacted outside BluedHealth during the study. Were they required to continue follow-up via telehealth in BluedHealth? Follow-up visit rates could significantly impact adherence.

Response: Thank you for the comment. We would like to clarify that only study invitation and initial screening were performed on BluedHealth platform, and the further recruitment, informed consent, baseline survey, and follow-ups were conducted through WeChat which is the dominant messaging and social application in China (more than 1.4 billion monthly active users in 2025), comparable to iMessage or WhatsApp in Western countries. Using a convenient messaging app for follow-ups may contribute to the optimal follow-up rates in the study. We have elaborated on this point in the revised Methods section.

(page 8, lines 150–156) *‘**Potentially eligible participants were then referred to the research team via WeChat, the most widely used messaging and social application in China (over 1.4 billion monthly active users, comparable to iMessage or WhatsApp), for further screening, informed consent, baseline survey, and follow-up surveys. WeChat was chosen because it is the most ubiquitous, secure, and familiar platform for the participants, which would make the communication between participants and investigators much easier and help increase the follow-up rates.’*

**Results Section**

R1.6 For participants lost to follow-up, did they continue filling prescriptions or attending visits? (Similar concern to 1.5)

Response: Following the response to R1.5, we would like to further clarify that if a participant lost to follow-up in a certain wave, the investigators would continue to contact and invite him to participate in the subsequent waves of follow-up through WeChat.

1) In the revised manuscript , we have clarified the follow-up procedure in the Methods section.

(page 9, lines 164–172) *‘**The baseline and 1-, 3-, and 6-months follow-up surveys were conducted by completing an anonymous and self-administered e-questionnaire. The link of the e-questionnaires was sent through WeChat. The participants were asked to click the link and log into the survey platform (www.wjx.com) using their unique ID as the passcode. In each wave of the surveys, several reminders for completing the questionnaires were sent to the participants through WeChat before identifying a participant as non-response or loss to follow-up. For participants who did not respond or loss to follow-up in a certain wave of the surveys, the investigators would continue to contact and invite them to participate in the subsequent waves of follow-up.’*

2) We have revised the Table 1 to clearly show the number of non-responses/loss to follow-up and related reasons for each survey wave.

 **Fig 1. Flowchart of the RECOPE study.**

3) In the revised manuscript, we further added a S1 Table to show the patterns of loss to follow-up/survey non-responses, and found that 7.1%, 8.5%, and 11.9% of the participants had three, two, and one time(s) of loss to follow-up.

S1 Table : Patterns of loss to follow-up of the study

| **Patterns** | **n** | **% (n/657)** |
| --- | --- | --- |
| Lost to follow-up (survey non-responses) for three times | 47 | 7.1% |
| Lost to follow-up (survey non-responses) for two times | 56 | 8.5% |
| Lost to follow-up (survey non-responses) for one time | 78 | 11.9% |
| Total | 181 | 27.5% |

R1.7 What is the rationale for using 7,000 CNY as the income threshold?

Response: We thank the reviewer for pointing this out.

1) First, we would like to acknowledge that 7,000 CNY is a typo in our original Tables and results section. In our analysis, we actually used 3,000 CNY as the dichotomized threshold for income and included this binary variable in the regression analyses, because it is the national average monthly income in China. We apologize for this confusion. We have clarified it in the revised measurement section in the Methods, and corrected 7000 CNY to 3000 CNY in tables for the subsequent regression analyses.

(page 11, lines 214–221) *‘**The participants were asked about their age (in years), sex (male, female), ethnicity (Han, others), employment status (students, unemployed, employed), education level (Junior high school or below, senior high school or equivalent, bachelor degree or equivalent, master degree or above), marital status (married/living with a partner, unmarried/divorced/separated/widowed), and monthly income (≤3000, 3001-5000, 5001-7000, 7001-10000, 10001-15000, 15001-20000, >20000 CNY). Income was further recoded into a binary variable using 3000 CNY (approximately 414 USD) as the threshold as it is the national average monthly income in China [18]’*

2) In the revised Table 1, we presented all the original categories of monthly income that used in the questionnaire. As you can see, 7000 CNY is actually the median level of monthly income in the study.

**Reference:**

[18] National Bureau of Statistics of China. National Bureau of Statistics of China (English) [Internet]. Beijing: National Bureau of Statistics of China; [cited 2025 Oct 13]. Available from: <https://www.stats.gov.cn/english>

**Table 1.** Distributions of socio-demographic characteristics in the RECOPE study.

| **Variables** | **Total** | | | | ***P ^a^*** |  | **ED regimen** | | | | ***P ^a^*** |  | **Daily regimen** | | | | ***P ^a^*** |
| --- | --- | --- | --- | --- | --- | --- | --- | --- | --- | --- | --- | --- | --- | --- | --- | --- | --- |
|  | **Baseline**  **(N=657)** | **1 month**  **(N=582)** | **3 month**  **(N=539)** | **6 month**  **(N=520)** |  |  | **Baseline**  **(n=529)** | **1 month**  **(n=458)** | **3 month**  **(n=428)** | **6 month**  **(n=422)** |  |  | **Baseline**  **(n=92)** | **1 month**  **(n=102)** | **3 month**  **(n=98)** | **6 month**  **(n=88)** |  |
| **Age (years, Mean±SD)** | 29.9±6.4 | 29.8±6.5 | 29.8±6.4 | 29.9±6.4 | 0.976 |  | 29.9±6.4 | 29.6±6.6 | 29.7 ±6.4 | 29.72 ± 6.5 | 0.938 |  | 30.4±6.4 | 31.00± 6.1 | 30.17 ±6.0 | 31.14 ±6.1 | 0.655 |
| 18-29 | 359 (54.6) | 322 (55.3) | 297 (55.1) | 278 (53.4) |  |  | 289 (54.6) | 260 (56.8) | 240 (56.1) | 235 (55.7) |  |  | 48 (52.2) | 48 (47.1) | 50 (51.0) | 38 (43.2) |  |
| 30-39 | 246 (37.5) | 214 (36.8) | 202 (37.5) | 203 (39.1) |  |  | 198 (37.5) | 164 (35.8) | 154 (36.0) | 155 (36.8) |  |  | 37 (40.2) | 47 (46.1) | 43 (43.9) | 43 (48.9) |  |
| >40 | 52 (7.9) | 46 (7.9) | 40 (7.4) | 39 (7.5) |  |  | 42 (7.9) | 34 (7.4) | 34 (7.9) | 32 (7.6) |  |  | 7 (7.6) | 7 (6.9) | 5 (5.1) | 7 (8.0) |  |
| **Designated Sex at Birth** |  |  |  |  | N/A |  |  |  |  |  | N/A |  |  |  |  |  | N/A |
| Male | 657 (100.0) | 582 (100.0) | 539 (100.0) | 520 (100.0) |  |  | 529 (100.0) | 458 (100.0) | 428 (100.0) | 422 (100.0) |  |  | 92 (100.0) | 102 (100.0) | 98 (100.0) | 88 (100.0) |  |
| Female | 0 (0.0) | 0 (0.0) | 0 (0.0) | 0 (0.0) |  |  | 0 (0.0) | 0 (0.0) | 0 (0.0) | 0 (0.0) |  |  | 0 (0.0) | 0 (0.0) | 0 (0.0) | 0 (0.0) |  |
| **Ethnicity** |  |  |  |  | 0.963 |  |  |  |  |  | 0.977 |  |  |  |  |  | 0.965 |
| Han | 615 (93.6) | 547 (94.0) | 508 (94.2) | 491 (94.3) |  |  | 497 (94.0) | 432 (94.3) | 405 (94.6) | 398 (94.3) |  |  | 85 (92.4) | 94 (92.2) | 92 (93.9) | 82 (93.2) |  |
| Others | 42 (6.4) | 35 (6.0) | 31 (5.8) | 29 (5.7) |  |  | 32 (6.0) | 26 (5.7) | 23 (5.4) | 24 (5.7) |  |  | 7 (7.6) | 8 (7.8) | 6 (6.1) | 6 (6.8) |  |
| **Employment** |  |  |  |  | 0.978 |  |  |  |  |  | 0.926 |  |  |  |  |  | 0.540 |
| Students/Unemployed | 130 (19.8) | 119 (20.4) | 112 (20.8) | 106 (20.3) |  |  | 114 (21.6) | 105 (22.9) | 96 (22.4) | 96 (22.4) |  |  | 9 (9.8) | 10 (9.8) | 13 (13.3) | 6 (6.8) |  |
| Employed | 527 (80.2) | 463 (79.6) | 427 (79.2) | 414 (79.7) |  |  | 415 (78.4) | 353 (77.1) | 332 (77.6) | 333 (77.6) |  |  | 83 (90.2) | 92 (90.2) | 85 (86.7) | 82 (93.2) |  |
| **Education Level** |  |  |  |  | 0.999 |  |  |  |  |  | 1.000 |  |  |  |  |  | 0.952 |
| Junior high school or below | 4 (0.6) | 2 (0.3) | 3 (0.6) | 3 (0.6) |  |  | 4 (0.8) | 2 (0.4) | 3 (0.7) | 3 (0.7) |  |  | 0 (0.0) | 0 (0.0) | 0 (0.0) | 0 (0.0) |  |
| Senior high school / Vocational high school / Technical secondary school | 29 (4.4) | 24 (4.1) | 22 (4.1) | 25 (48) |  |  | 23 (4.3) | 18 (3.9) | 17 (4.0) | 18 (4.3) |  |  | 6 (6.5) | 6 (5.9) | 5 (5.1) | 7 (8.0) |  |
| College diploma (Associate degree) / Bachelor’s degree | 436 (66.4) | 384 (66.0) | 358 (66.4) | 343 (65.9) |  |  | 353 (66.7) | 309 (67.5) | 287 (67.1) | 282 (66.8) |  |  | 54 (58.7) | 58 (56.9) | 61 (62.2) | 54 (61.4) |  |
| Master’s degree or above | 188 (28.6) | 172 (29.6) | 156 (28.9) | 149 (28.7) |  |  | 149 (28.2) | 129 (28.2) | 121 (28.3) | 119 (28.2) |  |  | 32 (34.8) | 38 (37.3) | 32 (32.7) | 27 (30.7) |  |
| **Marital Status** |  |  |  |  | 0.646 |  |  |  |  |  | 0.524 |  |  |  |  |  | 0.898 |
| Married or living with a partner | 90 (13.7) | 82 (14.1) | 81 (15.0) | 84 (16.2) |  |  | 77 (14.6) | 63 (13.8) | 68 (15.9) | 72 (17.1) |  |  | 11 (12.0) | 16 (15.7) | 13 (13.3) | 12 (13.6) |  |
| Unmarried/Divorced/Separated/Widowed | 567 (86.3) | 500 (85.9) | 458 (85.0) | 436 (83.8) |  |  | 452 (85.4) | 395 (86.2) | 360 (84.1) | 350 (82.9) |  |  | 81 (88.0) | 86 (84.3) | 85 (86.7) | 76 (86.4) |  |
| **Monthly income (CNY) ^b^** |  |  |  |  | 1.000 |  |  |  |  |  | 1.000 |  |  |  |  |  | 0.961 |
| ≤3000 | 77 (11.7) | 72 (12.4) | 67 (12.4) | 61 (11.8) |  |  | 62 (11.7) | 61 (13.3) | 55 (12.9) | 54 (12.8) |  |  | 9 (9.8) | 6 (5.9) | 9 (9.2) | 5 (5.7) |  |
| 3001-5000 | 92 (14.0) | 82 (14.1) | 77 (14.3) | 75 (14.5) |  |  | 78 (14.7) | 70 (15.3) | 64 (15.0) | 67 (15.9) |  |  | 9 (9.8) | 11 (10.8) | 13 (13.3) | 8 (9.1) |  |
| 5001-7000 | 103 (15.7) | 87 (14.9) | 82 (15.2) | 78 (15.0) |  |  | 91 (17.2) | 73 (15.9) | 71 (16.6) | 68 (16.1) |  |  | 9 (9.8) | 12 (11.8) | 11 (11.2) | 10 (11.4) |  |
| 7001-10000 | 121 (18.4) | 114 (19.6) | 107 (19.9) | 105 (20.2) |  |  | 95 (18.0) | 84 (18.3) | 77 (18.0) | 82 (19.4) |  |  | 18 (19.6) | 23 (22.5) | 25 (25.5) | 20 (22.7) |  |
| 10001-15000 | 127 (19.3) | 110 (18.9) | 104 (19.3) | 104 (19.8) |  |  | 101 (19.1) | 89 (19.4) | 81 (18.9) | 80 (18.9) |  |  | 19 (20.7) | 19 (18.6) | 21 (21.4) | 22 (25.0) |  |
| 15001-20000 | 57 (8.7) | 48 (8.2) | 42 (7.8) | 35 (6.7) |  |  | 38 (7.2) | 33 (7.2) | 29 (6.8) | 21 (5.0) |  |  | 15 (16.3) | 13 (12.7) | 11 (11.2) | 12 (13.6) |  |
| >20000 | 80 (12.2) | 69 (11.9) | 60 (11.1) | 62 (11.9) |  |  | 64 (12.1) | 48 (10.5) | 51 (11.9) | 50 (11.9) |  |  | 13 (14.1) | 18 (17.6) | 8 (8.2) | 11 (12.5) |  |

^a^ The Chi-square test was used for categorical variables and t-test was used for continuous variables.

^b^ CNY, Chinese Yuan (1 CNY=0.14 USD)

R1.8 The study used PHQ-9 for depression screening. Given the range from mild to severe depression, consider classifying participants by severity rather than a binary yes/no.

Response: Thanks. In the revised Methods section and Table 2, we now report the distribution of PHQ-9 severity using the standard five categories for descriptive presentation. For the subsequent regression analyses, we would like to clarify that we used the continuous variable of PHQ-9 in analysis to avoid information loss and to provide more stable estimates given the sparse counts in the higher-severity bands.

(page 13, lines 254–258) *‘**A summative score ranging from 0 to 27 was created by adding up the score of each item, and were categorized into standard severity groups for descriptive analysis: none/minimal (0-4), mild (5-9), moderate (10-14), moderately severe (15-19), and severe (20-27) (α in this study =0.90). In the regression analyses, the continuous score of PHQ-9 was used to minimize information loss.’*

**Table 2.** Distributions of sexual behaviors, PrEP-related variables, and psychosocial variables in the RECOPE study

| **Variables** | **Total** | | | | ***P ^d^*** |  | **ED regimen** | | | | ***P ^d^*** |  | **Daily regimen** | | | | ***P ^d^*** |  | **Continuous Sexually active^a^** |
| --- | --- | --- | --- | --- | --- | --- | --- | --- | --- | --- | --- | --- | --- | --- | --- | --- | --- | --- | --- |
|  | **Baseline**  **(N=657)** | **1 month**  **(N=582)** | **3 month**  **(N=539)** | **6 month**  **(N=520)** |  |  | **Baseline**  **(n=529)** | **1 month**  **(n=458)** | **3 month**  **(n=428)** | **6 month**  **(n=422)** |  |  | **Baseline**  **(n=92)** | **1 month**  **(n=102)** | **3 month**  **(n=98)** | **6 month**  **(n=88)** |  |  |  |
| **Sexually active** |  |  |  |  | **<0.001** |  |  |  |  |  | **<0.001** |  |  |  |  |  | 0.476 |  |  |
| Yes | 578 (88.0) | 454 (78.4) | 481 (89.2) | 470 (90.4) |  |  | 458 (86.6) | 339 (74.0) | 377 (88.1) | 374 (88.8) |  |  | 88 (95.7) | 100 (98.0) | 94 (95.9) | 88 (98.9) |  |  | 310 (100.0) |
| No | 79 (12.0) | 128 (21.6) | 58 (10.8) | 50 (9.6) |  |  | 71 (13.4) | 119 (26.0) | 51 (11.9) | 47 (11.2) |  |  | 4 (4.3) | 2 (2.0) | 4 (4.1) | 1 (1.1) |  |  | 0 (0.0) |
| **Chemsex^b^** |  |  |  |  | 0.564 |  |  |  |  |  | 0.338 |  |  |  |  |  | 0.803 |  |  |
| Yes | 247 (42.7) | 182 (40.1) | 202 (42.0) | 183 (38.9) |  |  | 202 (44.1) | 134 (39.5) | 157 (41.6) | 143 (38.2) |  |  | 35 (39.8) | 44 (44.0) | 44 (46.8) | 37 (42.0) |  |  | 160 (51.6) |
| No | 331 (57.3) | 272 (59.9) | 279 (58.0) | 287 (61.1) |  |  | 256 (55.9) | 205 (60.5) | 220 (58.4) | 231 (61.8) |  |  | 53 (60.2) | 56 (56.0) | 50 (53.2) | 51 (58.0) |  |  | 150 (48.4) |
| **Condom use^b^** |  |  |  |  | 0.090 |  |  |  |  |  | 0.303 |  |  |  |  |  | 0.790 |  |  |
| Inconsistent | 218 (37.7) | 201 (44.3) | 210 (43.7) | 206 (43.8) |  |  | 164 (35.8) | 141 (41.6) | 154 (40.8) | 151 (40.4) |  |  | 46 (52.3) | 56 (56.0) | 55 (58.5) | 52 (59.1) |  |  | 144 (46.5) |
| Consistent | 360 (62.3) | 253 (55.7) | 271 (56.3) | 264 (56.2) |  |  | 294 (64.2) | 198 (58.4) | 223 (59.2) | 223 (59.6) |  |  | 42 (47.7) | 44 (44.0) | 39 (41.5) | 36 (40.9) |  |  | 166 (53.5) |
| **Sexual partner^b^** |  |  |  |  | **<0.001** |  |  |  |  |  | **<0.001** |  |  |  |  |  | 0.240 |  |  |
| 1 | 110 (19.0) | 139 (30.6) | 125 (26.0) | 132 (28.1) |  |  | 84 (18.3) | 108 (31.9) | 109 (28.9) | 115 (30.7) |  |  | 17 (19.3) | 25 (25.0) | 13 (13.8) | 15 (17.0) |  |  | 53 (17.1) |
| >1 | 468 (81.0) | 315 (69.4) | 356 (74.0) | 338 (71.9) |  |  | 374 (81.7) | 231 (68.1) | 268 (71.1) | 259 (69.3) |  |  | 71 (80.7) | 75 (75.0) | 81 (86.2) | 73 (83.0) |  |  | 257 (82.9) |
| **Sexual role^b^** |  |  |  |  | 0.649 |  |  |  |  |  | 0.650 |  |  |  |  |  | 0.600 |  |  |
| Receptive or versatile | 267 (46.2) | 220 (48.5) | 222 (46.2) | 208 (44.3) |  |  | 222 (48.5) | 172 (50.7) | 176 (46.7) | 174 (46.5) |  |  | 34 (38.6) | 44 (44.0) | 44 (46.8) | 34 (38.6) |  |  | 155 (50.0) |
| Insertive | 311 (53.8) | 234 (51.5) | 259 (53.8) | 262 (55.7) |  |  | 236 (51.5) | 167 (49.3) | 201 (53.3) | 200 (53.5) |  |  | 54 (61.4) | 56 (56.0) | 50 (53.2) | 54 (61.4) |  |  | 155 (50.0) |
| **Commercial sex^b^** |  |  |  |  | 0.676 |  |  |  |  |  | 0.673 |  |  |  |  |  | 0.892 |  |  |
| Yes | 50 (8.7) | 36 (7.9) | 35 (7.3) | 44 (9.4) |  |  | 39 (8.5) | 27 (8.0) | 24 (6.4) | 31 (8.3) |  |  | 9 (10.2) | 9 (9.0) | 10 (10.6) | 11 (12.5) |  |  | 34 (11.0) |
| No | 528 (91.3) | 418 (92.1) | 446 (92.7) | 426 (90.6) |  |  | 419 (91.5) | 312 (92.0) | 353 (93.6) | 343 (91.7) |  |  | 79 (89.8) | 91 (91.0) | 84 (89.4) | 77 (87.5) |  |  | 276 (89.0) |
| **PrEP knowledge**^c^ |  |  |  |  | **0.014** |  |  |  |  |  | **0.002** |  |  |  |  |  | 0.590 |  |  |
| Correct | 455 (69.3) | 416 (71.5) | 410 (76.1) | 396 (76.2) |  |  | 390 (73.7) | 356 (77.7) | 356 (83.2) | 341 (81.0) |  |  | 42 (45.7) | 49 (48.0) | 46 (46.9) | 49 (55.1) |  |  | 233 (75.2) |
| In correct | 202 (30.7) | 166 (28.5) | 129 (23.9) | 124 (23.8) |  |  | 139 (26.3) | 102 (22.3) | 72 (16.8) | 80 (19.0) |  |  | 50 (54.3) | 53 (52.0) | 52 (53.1) | 40 (44.9) |  |  | 77 (24.8) |
| **Self-efficacy of PrEP adherence** | 3.9±1.8 | 4.2±1.8 | 4.4±1.8 | 4.3±1.9 | **<0.001** |  | 3.8±1.9 | 4.0±1.8 | 4.3±1.8 | 4.1±1.9 | **<0.001** |  | 4.6±1.6 | 5.1±1.4 | 5.1±1.3 | 5.1±1.6 | **0.041** |  | 4.1±1.7 |
| **PrEP-related stigma** | 25.1±6.9 | - | 25.0±7.1 | 24.9±6.6 | 0.084 |  | 25.3±6.7 | - | 25.4±6.9 | 24.7±6.7 | 0.275 |  | 23.2±7.3 | - | 23.3±7.4 | 25.7±6.6 | **0.011** |  | 25.1±4.6 |
| **Depressive symptoms** | 7.2±5.3 | - | 7.4±5.3 | 7.2±5.2 | 0.267 |  | 7.1±5.3 | - | 7.3±5.3 | 7.0±5.0 | 0.283 |  | 7.4±5.4 | - | 7.5±5.7 | 8.0±5.7 | 0.477 |  | 7.2±3.7 |
| None | 215 (32.7) | - | 172 (31.9) | 155 (29.8) |  |  | 175 (33.1) | - | 135 (25.3) | 129 (30.6) |  |  | 29 (31.5) | - | 30 (32.6) | 23 (25.8) |  |  | 65 (21.0) |
| Mild | 280 (42.6) | - | 233 (43.2) | 249 (47.9) |  |  | 223 (42.2) | - | 196 (37.1) | 207 (49.2) |  |  | 40 (43.5) | - | 27 (29.3) | 40 (44.9) |  |  | 103 (49.4) |
| Moderate | 97 (14.8) | - | 73 (13.5) | 67 (12.9) |  |  | 79 (14.9) | - | 61 (11.5) | 50 (11.9) |  |  | 13 (14.1) | - | 9 (9.8) | 14 (15.7) |  |  | 56 (18.1) |
| Moderate severe | 40 (6.1) | - | 43 (8.0) | 34 (6.5) |  |  | 32 (6.0) | - | 36 (6.8) | 25 (5.9) |  |  | 6 (6.5) | - | 5 (5.4) | 8 (9.0) |  |  | 20 (6.5) |
| Severe | 25 (3.8) | - | 18 (3.3) | 15 (2.9) |  |  | 20 (3.8) | - | 16 (30) | 10 (2.4) |  |  | 4 (4.3) | - | 2 (2.2) | 4 (4.5) |  |  | 16 (5.2) |
| **Resilience** | 5.2±1.9 | - | 5.7±1.7 | 5.8±1.6 | **<0.001** |  | 5.2±1.9 | - | 5.7±1.7 | 5.7±1.7 | **<0.001** |  | 5.0±2.1 | - | 5.7±1.7 | 6.0±1.6 | **<0.001** |  | 5.7±1.2 |

^a^ Chemsex, and commercial sex were classified as “yes” if participants reported engaging in these behaviors at least once across the two follow-up assessments. If participants reported Having multiple homosexual partners at least once during the two follow-ups, they were classified as “> 1”. If participants reported Inconsistent condom use at least once during the two follow-ups, they were classified as “Inconsistent”. If participants reported their sexual role as receptive or versatile at least once during the two follow-ups, they were classified as “receptive or versatile”. Self-efficacy for being adhere to PrEP, resilience, depressive symptoms, and PrEP-related stigma in the last column were assessed using the mean scores from the two follow-up assessments.

^b^ For chemsex, condom use, sexual partner, sexual role, and commercial sex, the total sample for these variables are those who have reported being sexually active for the past study duration.

^c^ PrEP-related knowledge was assessed by asking participants to identify correct dosing instructions based on their regimen (daily or ED). Only those who selected all correct options were coded as having correct PrEP adherence knowledge.

^d^ The Chi-square test was used for categorical variables and t-test was used for continuous variables.

R1.9 Figures and Data Presentation: Figure 2 has poor resolution and adds limited value. Consider revising, for example by combining graphs (e.g., comparing event-driven and daily PrEP in the same figure). PrEP adherence often varies within individuals over time. Consider presenting this variability using a heatmap to illustrate adherence patterns.

Response: Thanks. We have modified Figure 2 per your suggestion. It now has four sub-figures, 1) 2a shows the prevalence of optimal adherence in the past 1 month among ED users, 2) 2b shows the prevalence of optimal adherence in the past 3 month among ED users, 3) 2c shows the proportions of ED users who had consistent adherence, partially consistent adherence, and fully non-adherence, 4) 2d shows the prevalence of optimal adherence in the past one month of daily users. We also added a heatmap indicator (0%-100%) on the right side to illustrate the variability.

**Fig 2. Prevalence of optimal PrEP Adherence by PrEP regimens and outcome definitions in the RECOPE study.**

**Discussion**

R1.10 The discussion should be consolidated and organized by theme. Currently, similar points are repeated in different sections/paragraphs.

Response: We thank the reviewer for this comment. In the revised manuscript, we have improved the clarity and flow of the Discussion by adding thematic subheadings and streamlined repeated statements to reduce redundancy while retaining all key points. These headings highlight the main themes of the study findings —such as PrEP adherence (by event-driven and daily regimen), psychosocial and behavioral correlates of adherence (self-efficacy, high-risk behaviors, stigma, and age), study limitations, and conclusions.

R1.11 The first paragraph aligns with the introduction, but subsequent sections lack coherence. For example, while 94% of participants were already using PrEP at baseline and most answered PrEP-related questions correctly, what evidence supports the statement: "inadequate instruction and counseling on medication use among e-consumers" (line 340)?

Response: Thanks.

1) We have revised the Discussion to improve coherence and to link each interpretive statement to supporting evidence.
2) Regarding the statement “inadequate instruction and counseling on medication use among e-consumers,” we have clarified that our data provided some indirect evidence supporting it. Although 94% of the participants were already using PrEP at baseline, there were still about one quarter of the event-driven users and half of the daily users who did not correctly answer all the PrEP knowledge questions. In addition, the top self-reported reasons for non-adherence among ED users were “I used other HIV prevention” and “I trusted my partner.” These findings indicate substantial gaps in regimen-specific knowledge, risk appraisal, and practical skills for aligning PrEP use among the participants, suggesting an inadequate instruction and counseling on medication use among e-consumers.

(page 25, lines 424–430) *‘Some possible explanations for the suboptimal adherence in the RECOPE study include potential gaps in instruction and counseling on medication use among e-consumers (indicated by the findings that about one quarter of the ED users did not correctly answer all the knowledge questions, and the top self-reported reasons for non-adherence included ‘I used other HIV prevention’ and ‘I trusted my partner’), the complexity of the ED regimen, lack of a systematic reminder mechanism, and difficulty in predicting sexual activity…….’*

**Minor Comments**

R1.12 Please confirm the age range in Table 1. "28-29" appears to be an error; likely "18-29."

Response: Thanks. We have corrected this typo to “18-29” accordingly (see revised Table 1).

R1.13 Ensure consistent citation formatting. Some references are difficult to track. If a reference is only available in Chinese, please indicate this clearly.

Response: Thanks. We have carefully checked and standardized all citation formatting throughout the manuscript and reference list to ensure consistency with journal style. For references that are only available in Chinese, we have clearly indicated “(in Chinese)” to make it explicit.

*********************************************************************

**Reviewer #2**

Thanks for the opportunity to read your manuscript. My role is statistical reviewer, so I have focused on the design, data, and analysis that are presented. I have put general comments first, followed by questions relevant to a specific section of the manuscript (with a page/line reference).

This study estimates adherence to PrEP in MSM from China who sourced PrEP from an electronic platform. Data is a from a prospective cohort of consumers, recruited in 2023 and 2024 from online platform for PrEP, and with follow-up data collection at 1, 3, and 6 months. The recruitment rate for the study was good (657/680 eligible participants) and a response rate at 6 months of 80% was achieved. Adherence was defined as reporting taking PrEP before each sexual encounter inline with '2-1-1' dosing, or taking daily PrEP. A range of personal characteristics and psycho-social variables were collected at baseline, as well as sexual behaviour and knowledge, stigma, and self-efficacy around PrEP use. Prevalence of adherence was reported stratified by type of PrEP use at each data collection point. A regression model estimating the association between different personal factors and adherence in the ED users, examining 1-month, 3-month, and consistent adherence.

Response: Thank you for all your helpful comments for the statistical analysis. We have carefully considered them and provided a point-by-point response below.

R2.1 It looks like for the regression models, an odds ratio for a model with just each factor and time was estimated, as well as an overall multivariable model with all factors included. A drawback to the multivariable model is that the interpretation of odds ratios because more difficult, because depending on the purported relationship between these, they will be a mix of 'total' effects and 'direct' effects (i.e. table 2 fallacy, https://doi.org/10.1093/aje/kws412). E.g., if age influences self-efficacy, then the odds ratio of age and self-efficacy from the multivariable model have different interpretations. Given the study aims are not causal and are more descriptive, I would consider just presenting the odds ratios from the univariate models.

Response: Thanks.

1) First, we agree that a drawback of multivariable model is the difficulties in interpreting the strength of associations such as odds ratio (Westreich & Greenland, Am J Epidemiol, 2013). However, the primary aim of the regression analysis in the study was to explore whether the exposure factors of interest were significantly and independently associated with the adherence outcome, which means we focused more on the significance of association (indicated by 95% CI of OR and p-value) rather than the strength of association.
2) Second, although univariate regression analysis can provide information on the significance of association, however, it cannot provide information on significance of independent association, as there may exist confounders of the associations. As shown in the correlation figure below, several pairs of sociodemographic and behavioral variables in the study showed significant correlations, indicating confounding should not be ignored when examining the independent association between an exposure variable and the outcome.

For example, PrEP-related stigma is significantly correlated with both the adherence and self-efficacy, and self-efficacy is significantly correlated with adherence. The significant association between self-efficacy and adherence shown in the univariate regression may be due to either the independent effect of self-efficacy on adherence or the confounding bias introduced by stigma. Therefore, we choose to further perform a multivariable regression to examine the independent association between self-efficacy and adherence with stigma and other potential confounders (covariates) being controlled.

3) In the revised Statistical analysis section in the manuscript, we have made it explicit that the three regression models were fitted to examine the statistical significance of crude and independent associations between exposure variables of interest and the adherence outcomes.

(page 14, lines 272–285) ‘*Second, regression analyses were performed to examine the statistical significance of crude and independent associations between all the exposure variables of interest (socio-demographics, psycho-behavior variables) and the adherence outcomes. For ED users, three regression models were fitted for the three adherence outcomes: (1)* ***model 1*** *included a univariate generalized linear mixed model (GLMM) with a logit link and random intercept to evaluate the statistical significance of crude association between each exposure variable and the past three-month adherence, as well as a multivariable GLMM including all exposure variables of interest in order to evaluate the significance of their independent associations with the outcome after controlling potential confounders (covariates); (2)* ***model 2*** *were similar univariate and multivariable GLMM for the past one-month adherence outcome; (3)* ***model 3*** *included a univariate and a multivariable logistic regression model for the consistent adherence outcome, in which baseline socio-demographics and averaged time-varying values at 3- and 6-months for other exposure variables were included as independent variables.…….’*

**R2.2** P6, L116: What was the reason to include the design effect adjustment, and what parameters were used to estimate this design effect?

Response: Thanks.

1) We included the design effect adjustment in the sample size calculation, mainly because a convenience sampling method, rather than a random sampling method, was used in the study to recruit e-consumers of PrEP (mostly MSM) which is a marginalized population in China. The convenience recruitment procedure might result in that people with similar characteristics might be more likely to be recruited in the study. Therefore, we introduced a design effect of 1.1 as a conservative safeguard to assure sufficient sample size/statistical power.
2) Following previous HIV-related studies among MSM [1,2], we applied the standard formula deff=1+(m-1)×ICC to calculate the design effect and assumed an intraclass correlation coefficient (ICC) of 0.01, yielding a final design effect of 1.1.
3) Importantly, our final sample size (N=657 at baseline) far exceeds the planned sample size (424), so the robustness of the study results is not sensitive to the assumption of design effect.
4) We have incorporated the above statements in the Study Protocol (S1 File)

**References:**

[1] Sulaberidze, L., Mirzazadeh, A., Chikovani, I., Shengelia, N., Tsereteli, N., & Gotsadze, G. (2016). Population Size Estimation of Men Who Have Sex with Men in Tbilisi, Georgia; Multiple Methods and Triangulation of Findings. PloS one, 11(2), e0147413. https://doi.org/10.1371/journal.pone.0147413.

[2] Wendland, E., Vieira, B. A., Eidt, G., Ikeda, M. L. R., de Souza, F. M. A., Tonini, M. L., Gaspar, P. C., Hallal, R. C., Moherdaui, F., Pereira, G. F. M., & Bidinotto, A. B. (2025). A serological household survey on social determinants of the generalized HIV epidemic in southern Brazil. Scientific reports, 15(1), 25476. https://doi.org/10.1038/s41598-025-06764-6.

**R2.3** P7, L134: I would move the information about recruitment and participant flow to the results.

Response: Thanks. We have moved the information per your suggestion.

(page 15, lines 302–312) *‘Of the 877 individuals invited, 680 were eligible for the study and sent the baseline questionnaire (146 did not respond to the study invitation, 33 were not interested for participation after further communication with the recruiters, 18 had no plan to use PrEP in the future six months), and 657 completed the baseline survey (23 did not respond to the questionnaire due to break-off) and were finally enrolled in the RECOPE cohort. According to the American Association for Public Opinion Research (AAPOR)’s standard definitions, the response rate of the baseline survey was 96.6% (657/680). At the 1-, 3-, and 6-month follow-ups, 90.1% (592/657), 82.6% (543/657), and 80.1% (526/657) of the participants responded to the questionnaire surveys and were successfully followed-up, with 582, 539, and 520 being included in the analysis after excluding those failing the quality check of questionnaires (Fig. 1). ’*

**Fig 1. Flowchart of the RECOPE study.**

**R2.4** P12, L232: Was an interaction between time-point and factors included? Or was the effect of the covariate assumed to be constant over the study?

Response: Thanks. In our analysis, we did not include interaction terms between time-point and exposure variables of interest, mainly because,

1) we assumed that the effects of exposure variables on PrEP adherence would remain relatively stable during the limited number of repeated measurement in the study (four surveys over six months). This assumption is supported by prior longitudinal PrEP studies [26].
2) For Model 1 and Model 2 in the analysis, we used GLMM with a logit link, in which the fixed effects represent the average (population-level) association of exposure variables with adherence, while the random effects accommodate subject-specific deviations and account for within-participant correlation across repeated measures in different survey waves. Compared with marginal models (e.g., generalized estimating equations, GEE), GLMM permits subject-specific inference and better handles hierarchical structure [27]. Inclusion of multiple interaction terms (e.g. time × exposure variable) in GLMM when the number of repeated measurement is small may introduce issues like overfitting, inflated standard errors, convergence issues, and difficulty in interpretation. One should balance model complexity and interpretability, especially in longitudinal designs with a limited number of repeated measures [28].
3) We have incorporated related statements in the revised statistical section.

(page 14, lines 289–301) *‘All the above GLMM included fixed effects representing the average (population-level) association of the exposure variables with adherence outcomes, and random effects accounting for within-participant correlation of repeated measures across different survey waves. The models did not include interaction terms between time-points and the exposure variables, as it was assumed that the effects of exposure variables on PrEP adherence would remain relatively stable due to limited number of repeated measurement in the study (four survey waves over six months) [26–28] ……’*

**Reference:**

[26] Chu ZX, Jin X, Ye ZH, et al. Real-time digital intervention on oral pre-exposure prophylaxis adherence among MSM: randomized controlled trial. npj Digit Med. 2025;8:349.

[27] Twisk JWR. Applied Longitudinal Data Analysis for Epidemiology: A Practical Guide. 2nd ed. Cambridge University Press; 2013.

[28] Cheng J, Edwards LJ, Maldonado-Molina MM, Komro KA, Muller KE. Real longitudinal data analysis for real people: building a good enough mixed model. Stat Med. 2010;29(4):504–520.

**R2.4** P12, L233:What covariates were included in these models?

Response: Thanks. As mentioned in the response to R2.1, the primary aim of the regression analysis in the study was to widely explore whether the exposure variables of interest (socio-demographics and psycho-behavioral variables) were significantly and independently associated with the adherence outcome. In the multivariable regression analysis, we included all the exposure variables of interest in the model; when interpreting the odds ratio (95% CI and p-value) of one exposure variable, the other exposure variables included in the regression could be considered as covariates (potential confounders).

(page 14, lines 272–281) ‘*Second, regression analyses were performed to examine the statistical significance of crude and independent associations between all the exposure variables of interest (socio-demographics, psycho-behavior variables) and the adherence outcomes. For ED users, three regression models were fitted for the three adherence outcomes: (1)* ***model 1*** *included a univariate generalized linear mixed model (GLMM) with a logit link and random intercept to evaluate the statistical significance of crude association between each exposure variable and the past three-month adherence, as well as a multivariable GLMM including all exposure variables of interest in order to evaluate the significance of their independent associations with the outcome after controlling potential confounders (covariates)…….’*

*********************************************************************

**Reviewer #3**

This paper describes self-reported adherence to PrEP among a convenience sample of people obtaining PrEP online in China. Optimal (i.e., complete) adherence to event-driven PrEP was suboptimal (41-53%) and better (84-88%) for daily PrEP. Key factors identified included age, self-efficacy, and types of sexual behavior. Strengths of the paper include the real-world context, repeated measures, and sample size. Limitations include the reliance on subjective information, lack of representative sampling, and lack of attention to longitudinal prevention needs. Overall, the paper is of interest and adds to the literature on "real-world" adherence to PrEP; however, the take-home messages about challenges in PrEP adherence are well-established.

Response: We thank the reviewer for the careful review. We have carefully considered and addressed them. A detailed point-by-point response is below.

**Some overarching questions**

**R3.1** The study included only MSM despite MSM not being an inclusion criterion. This point is worth noting in the paper. The sample was also highly educated and Han. If online dispensing is the primary means for getting PrEP, other delivery systems in China are needed.

Response: Thanks.

1) We confirm that being MSM is not an inclusion criterion. In the study, all the participants were MSM. Although MSM account for the majority of PrEP users in China, however, we acknowledge that it also indicated a limited representativeness of the study sample, as those who had a heterosexual orientation were completely not captured in the study.
2) Other socio-demographic characteristics, such as ethnicity and education, also provided information on representativeness of the study sample. For example, some characteristics of the study sample were different with previous PrEP-related study in China (e.g., education level and income level were higher in the study), while some were consistent to previous PrEP-related study or the overall Chinese population (e.g., 91.11 % of the total population in China are Han Chinese, similar to the proportion of Han people in the study sample).
3) We agree with the reviewer that although online dispensing is the primary means for getting PrEP in China, research on other delivery systems in China are still needed.
4) In the revised Limitation in the Discussion section, we have added a separate paragraph discussing the representativeness of the sample and highlighting the need for study on other delivery systems in China.

(page 30-31, lines 535–551) *‘Furthermore, the convenience sampling from a single e-commerce platform may also lead to limited representativeness of the study sample. Caution is warranted when generalizing the study findings to all e-consumers of PrEP in China. For example, all the participants in the study were MSM. Although it is true that MSM account for the majority of PrEP users in the country, those with a heterosexual orientation were completely not captured in the study. In addition, 95% of the participants had an education level of college or above, higher than that in previous PrEP studies in China (e.g., 70.0%-84.1% received college or above education) [12,47,48]. The study also reported a higher monthly income than previous studies among Chinese MSM [12,47,48]. Some other characteristics of the study sample were comparable to previous PrEP-related studies in China or the context of overall Chinese population. For example, 93.6 % of the study participants were Han people. According to the latest National Population Census in China, Han ethnicity accounted for 91.11 % of the total population [49]. Last but not least, the study only included people who bought PrEP online. However, other PrEP delivery systems such as hospitals, clinics, and CDC are also important venues for promoting PrEP use. Future research on these dispensing venues will be needed.’*

**References:**

[12] Zhu Y, Tian M, Yin W, et al. The compliance of HIV pre-exposure prophylaxis uptake among MSM. Chinese Journal of AIDS & STD 2023;05. doi: 10.13419/j.cnki.aids.2023.05.11 [In Chinese]

[47] Zhang, G., Yang, X., Kang, W. et al. Pre-exposure prophylaxis uptake for high-risk men who have sex with men in China: a multi-city cross-sectional survey. AIDS Res Ther. 2023;32(20). [doi: 10.1186/s12981-023-00528-w](https://doi.org/10.1186/s12981-023-00528-w)

[48] Zhang J, Xu JJ, Wang HY, et al. Preference for daily versus on-demand pre-exposure prophylaxis for HIV and correlates among men who have sex with men: the China Real-world Oral PrEP Demonstration study. J Int AIDS Soc. 2021;24(2). doi:10.1002/jia2.25667

[49] National Bureau of Statistics of China. Main Data of the Seventh National Population Census (2021). https://www.stats.gov.cn/english/PressRelease/202105/t20210510_1817185.html

**R3.2** If participants were using condoms (a primary cited reason for not taking PrEP), is PrEP adherence needed? Condoms are an effective HIV prevention tool. I recommend the authors consider the concept of prevention-effective adherence, in which PrEP use is aligned with potential risk exposure (see Haberer et al, AIDS 2015).

Response: Thanks for the suggestion. We agree with the reviewer that the concept of “prevention-effective adherence” proposed by Haberer et al is worthy for consideration, which emphasizes evaluating medication use in relation to periods of HIV risk and other prevention tools [1]. However, we still think PrEP adherence is needed even if the participants reported they had used condoms in the study. That’s because:

1) the self-reported condom use did not mean that the participants had actually used condoms correctly throughout the entire sexual intercourse due to reporting bias and their misunderstanding about ‘correct use of condoms’, and thus did not mean they had a low risk of HIV infection and didn’t need a good PrEP adherence. The prevention-effective adherence may under-address the actual HIV risk of the participants in the study.
2) both the international and China guideline emphasize a combination use of PrEP and other preventive measures such as condoms, and emphasize the necessity and importance of keeping optimal adherence of PrEP regardless of use of other preventive measures such us condoms [2-4]. The guidelines did not state that PrEP adherence is not necessary if a PrEP user was using condoms. The authors from China CDC also strongly recommended not to report prevention-effective adherence in the study, which may lead to some misunderstandings by the real-world PrEP users in China and increase their risk of HIV infection (e.g., they may both not use condoms correctly although they think they did and not take PrEP correctly).
3) the concept of “prevention-effective adherence” was developed mainly for daily PrEP use. In our study, the majority of the participants used event-driven PrEP, and their patterns of sexual activities are very complex, which makes the measurement of prevention-effective adherence more difficult and harder to interpret.

**References:**

[1] Haberer JE, Bangsberg DR, Baeten JM, Curran K, Koechlin F, Amico KR, et al. Defining success with HIV pre-exposure prophylaxis: a prevention-effective adherence paradigm. AIDS 2015;29:1277.

[2] WHO. Condoms. https://www.who.int/news-room/questions-and-answers/item/condoms

[3] US Preventive Services Task Force. Prevention of Human Immunodeficiency Virus (HIV) Infection: Preexposure Prophylaxis. https://www.uspreventiveservicestaskforce.org/uspstf/recommendation/prevention-of-human-immunodeficiency-virus-hiv-infection-pre-exposure-prophylaxis

[4] Xu, J. J., Huang, X. J., Liu, X. C., Wang, L. M., Chen, Y. K., Wang, H., Zhang, F. J., Wu, H., Li, T. S., Han, M. J., Zhao, F., Ding, H. B., Duan, J. Y., Sheng, G. S., & Shang, H. (2020). Consensus statement on human immunodeficiency virus pre-exposure prophylaxis in China. Chinese medical journal, 133(23), 2840–2846.

In the revised discussion section, we discussed that why ‘prevention-effective adherence’ was not used and suggest using it in future studies if more rigorous measurement of condoms use and other HIV risk exposure variables are used.

(page 26, lines 430–440) ‘……*Additionally, the definition of optimal adherence used in this study (complete compliance with the ‘2-1-1’ dosing schedule for each sex activity in the past one/three months) was stringent and did not consider actual risk exposure to HIV. For example, the most commonly reported reason for non-adherence was use of condoms, which infers that some non-adhere ED users might still be able to protect themselves from HIV infection due to correct condom use. However, since self-reported measures of condom use in the study might introduce recall bias and social desirability bias, it is difficult to evaluate the real exposure risk of HIV in the study. Future studies may consider to calculate prevention-effective adherence in which PrEP use is aligned with potential risk exposure [31], if more rigorous measurement of condoms use and other HIV risk exposure variables are used.* ’

**Reference:**

[31] Haberer JE, Bangsberg DR, Baeten JM, Curran K, Koechlin F, Amico KR, et al. Defining success with HIV pre-exposure prophylaxis: a prevention-effective adherence paradigm. AIDS 2015;29:1277.

**R3.3** While the article is largely clear and well-written, the grammar should be reviewed by a native English speaker.

Response: Thanks. The revised manuscript has been thoroughly reviewed and edited by a native English speaker to ensure grammatical accuracy.

**Some detailed comments follow:**

**Abstract**

**R3.4** Line 7: I would remove "first ever", which sounds sensational (in the Abstract and in the paper).

Response: Thanks. We have removed the “first ever” from the Abstract andmain text.

**R3.5** Line 9: Add "modifying" after "potential".

Response: We have revised the wording to “potential modifying factors” accordingly.

**R3.6** Results: I would report the adherence rates for daily PrEP as well.

Response: We thank the reviewer for this helpful suggestion.

1) In the revised abstract, we have now added the adherence rates for daily PrEP users.

(page 2, lines 39–40) *‘The adherence rate among daily PrEP users at each wave ranged 97.1-98.7%.’*

2) In the revised Methods section, we have introduced the calculation of adherence rate for daily users.

(page 10-11, lines 205–207) ‘*……The adherence rate in the past month of each daily user was also calculated following the formular:*

*Adherence rate (%) =* $\frac{30-number of missing pills}{30}$ *×100%*’

3) In the revised Results section, we have added the results of adherence rates and added a Table S3 to present it.

(page 22, lines 373–378) *‘Among the daily regimen users, the prevalence of optimal adherence (and the median [IQR] number of pills missed in the past month) was 83.7% (2 [1.5]), 81.4% (2 [2.0]), 87.8% (2 [1.0]), and 84.3% (2 [2.0]) in the four survey waves, respectively (Fig 2d). The mean adherence rates in the past one month were 98.7% (SD = 4.3), 97.8% (SD = 10.2), 99.3% (SD = 2.4), and 97.1% (SD = 13.1) at baseline and 1-, 3-, and 6-months follow-ups, respectively (S4 Table).’*

S4 Table: Adherence among PrEP users across four study visits

| PrEP regimen | Visit | N | Optimal adherence n (%)^a^ | Adherence rate (%; mean±SD)^b^ |
| --- | --- | --- | --- | --- |
| Daily | Baseline | 92 | 77 (83.7) | 98.7 ± 4.3 |
|  | 1 month | 102 | 83 (81.4) | 97.8 ± 10.2 |
|  | 3 month | 98 | 86 (87.8) | 99.3 ± 2.4 |
|  | 6 month | 89 | 75 (84.3) | 97.1 ± 13.1 |
| ED (past one month) | Baseline | 273 | 154 (41.3) | N/A |
|  | 1 month | 339 | 146 (43.1) | N/A |
|  | 3 month | 307 | 139 (45.3) | N/A |
|  | 6 month | 304 | 160 (52.6) | N/A |
| ED (past three month) | Baseline | 458 | 177 (38.6) | N/A |
|  | 3 month | 377 | 163 (43.3) | N/A |
|  | 6 month | 374 | 165 (44.1) | N/A |

a: “Optimal adherence” was defined as taking all prescribed doses without any missed doses in the past month for participants on the daily PrEP regimen, and as strict compliance with the recommended “2-1-1” dosing schedule (two pills 2–24 hours before sex, one pill 24 hours after, and one pill 48 hours after) for those on the event-driven (ED) PrEP regimen.

b: Adherence rate (%) = (30-missed doses)/30 × 100.

**Introduction**

**R3.7** Line 66: Adherence was a key issue in the clinical trials as well (e.g., the low adherence in FEM-PrEP and VOICE showed no efficacy; high documented adherence in Partners PrEP was a key factor in the approval and normative recommendations of PrEP). I would not over-emphasize its importance in the real-world (vs trials).

Response: Thanks. We agree that PrEP adherence is important in both clinical trials and real-world use. We have revised the statements accordingly.

(page 4, lines 69–72) *‘**The success of PrEP is highly dependent on its medication adherence in both clinical/implementation trials and real-world settings. A meta-analysis suggests that every 10% decline in PrEP adherence may reduce its effectiveness by approximately 13% [7]…….’*

**Reference:**

[7] Murchu EO, Marshall L, Teljeur C, Harrington P, Hayes C, Moran P, et al. Oral pre-exposure prophylaxis (PrEP) to prevent HIV: a systematic review and meta-analysis of clinical effectiveness, safety, adherence and risk compensation in all populations. BMJ Open 2022;12:e048478. <https://doi.org/10.1136/bmjopen-2020-048478>.

**R3.8** Line 69: While percent adherence certainly has a role in its effectiveness, it is also important to align PrEP use with potential exposure (see my comment above). Risk is not constant and may explain how seemingly low PrEP adherence can still be effective in HIV prevention.

Response: As explained in the response to comment R3.2, we did not consider the align of PrEP use with potential exposure to HIV in the study for several reasons (e.g., self-reported measures on condom use and other preventive measures might be biased, the current official guidelines of PrEP do not mention the adherence is unnecessary if using condoms, and the majority of the study sample were ED users). We have suggested future research to better address this issue in the revised Discussion section.

**R3.9** Line 90: I wonder why being online makes medication instructions and counseling difficult. The authors themselves argue it is more convenient and less stigmatized.

Response: Thanks. In the revised Introduction section, we have elaborated on the flow of purchasing PrEP online and why would online makes medication instructions and counseling difficult.

(page 5, lines 89–107) *‘……Despite its advantages in convenience and privacy protection, the current online services of PrEP present some disadvantages in providing comprehensive instructions and counseling on PrEP use, which may pose distinct challenges for maintaining optimal medication adherence. For instance, when people are willing to purchase PrEP through BluedHealth, the platform would first require them to complete an online screening questionnaire for HIV exposure risk and PrEP eligibility (e.g., recent HIV testing result, contraindications to PrEP) [13]. Then a doctor would review the screening questions remotely and issue an electronic prescription and provide a standard written instructions on PrEP use if the clients met PrEP eligibility. No other interactive counselling would be provided at this stage. If a client had questions, he/she needs to join another online counselling group on BluedHealth for further communication. In contrast, during offline clinic visits for PrEP acquisition, in-person, face-to-face, and one-on-one counselling can be directly provided by doctors during and after the visit. It allows for immediate feedback and counseling adjustments, more nuanced history-taking of PrEP users, and direct observation of nonverbal cues. Therefore, compared with offline counselling, online counselling has a poorer accessibility, more complex procedure, and less flexibility of interacting with doctors, which may negatively affect clients’ understanding about PrEP use and ultimately reduce medication adherence.…….’*

**Methods**

**R3.10** Line 108: Please indicate the corresponding author's affiliated organization directly.

Response: Thanks. We have indicated the corresponding author’s affiliated organization directly as the “Institutional Review Board of Tsinghua University” in the revised manuscript. (page 7, lines 123–125)

**R3. 11** Line 118: I don't know that the equation for a sample size calculation is necessary for this type of publication (i.e., the described methods and reference seem sufficient).

Response: Thanks. Per the Editor’s suggestion, we have moved the sample size calculation to the supplementary file of study protocol (S1 File).

**R3. 12** Lines 125-127: What does being "qualified for PrEP use" mean? How did the authors establish whether someone had "communication barriers or reading disabilities"? It seems that not completing the baseline survey was also considered as an exclusion criterion (line 137), which should be more clearly explained with the other exclusion criteria.

Response: We thank the reviewer for the comments.

- **Definition of “qualified for PrEP use.”** According to the 2023 Chinese expert consensus and WHO guidelines, being “qualified for PrEP use” means that the individual is HIV-negative and at high risk of acquiring HIV [1]. This typically includes people who, in the past few months, have had condomless anal or vaginal sex, have an HIV-positive partner whose viral load is not confirmed as suppressed, have been newly diagnosed with a sexually transmitted infection, inject drugs and share equipment, or have repeatedly used post-exposure prophylaxis. Before starting PrEP, medical evaluation should also exclude acute HIV infection and contraindications to PrEP drugs (such as significant renal impairment) and document HBV status and, for women, pregnancy status. Only individuals meeting these behavioral-risk and medical-safety criteria are considered qualified for PrEP use under these guidelines. We have added these details to the study protocol (S1 File).
- **Assessment of communication or reading barriers.** The assessment on whether the potential participants had communicating or reading barriers was completed by recruiters from BluedHealth during study invitation and initial screening (e.g., being unable to read the invitation message or difficult to communicate with the recruiters) and further checked by researchers when they were referred to the study team for further screening. We have added these details to the study protocol (S1 File).
- **Non-completion of the baseline survey.** We would like to clarify that during the study recruitment (e.g. information shown in the invitation message and recruitment flyers), fail to complete baseline questionnaire was not listed as an exclusion criterion. We exclude those who were eligible and agreed to participate yet failed to complete the baseline survey (because they no longer responded to our contact) from the final cohort enrollment, as we could not contact these people anymore and did not have any information on them. The revised Results section and Figure 1 clearly show the recruitment and follow-up process.

(page 15, lines 302–312) *‘Of the 877 individuals invited, 680 were eligible for the study and sent the baseline questionnaire (146 did not respond to the study invitation, 33 were not interested for participation after further communication with the recruiters, 18 had no plan to use PrEP in the future six months), and 657 completed the baseline survey (23 did not respond to the questionnaire due to break-off) and were finally enrolled in the RECOPE cohort. According to the American Association for Public Opinion Research (AAPOR)’s standard definitions, the response rate of the baseline survey was 96.6% (657/680). At the 1-, 3-, and 6-month follow-ups, 90.1% (592/657), 82.6% (543/657), and 80.1% (526/657) of the participants responded to the questionnaire surveys and were successfully followed-up, with 582, 539, and 520 being included in the analysis after excluding those failing the quality check of questionnaires (Fig. 1).’*

**Fig 1. Flowchart of the RECOPE study**

**Reference:**

[1] Xu, J. J., Huang, X. J., Liu, X. C., Wang, L. M., Chen, Y. K., Wang, H., Zhang, F. J., Wu, H., Li, T. S., Han, M. J., Zhao, F., Ding, H. B., Duan, J. Y., Sheng, G. S., & Shang, H. (2020). Consensus statement on human immunodeficiency virus pre-exposure prophylaxis in China. Chinese medical journal, 133(23), 2840–2846. https://doi.org/10.1097/CM9.0000000000001181

**R3. 13** Line 132: I understand the consenting procedures, but "oral" does not seem to be the appropriate descriptor (perhaps "anonymous consent"?).

Response: Thanks. We have modified the description accordingly in the revised Methods section.

(page 8, lines 158–161) *‘……. Those who were eligible and agreed to join the study were then asked to provide an anonymous informed consent by clicking the agreement button in an e-informed consent form sent by the researcher via WeChat to preserve anonymity.’*

**R3.14** Lines 142 and 149-150: I would expect to see the follow-up rates/sample sizes in the Results section. I am also confused how the follow-up rate was 90.1% at baseline when completion of the survey was a requirement for enrollment.

Response: As mentioned in the response to R3.12, we have modified the description of follow-up rates in text and Figure 1 and moved them to the Results section. We would also like to clarify that 90.1% is the 1-mon follow-up rate.

**R3. 15** Line 168: It appears the baseline adherence depended on 1 month of PrEP use, but the enrollment criteria did not specify that requirement. Did all participants have at least 1 month of PrEP use prior to enrollment?

Response: Thanks.

1) First, we would to confirm that PrEP use in the past 1 month or 3 month was not an enrollment criterion in the study.
2) Second, when calculating the outcome variables (past 1-month adherence, past 3-month adherence) at baseline, those who had never initiated PrEP and those who had no sexual activities during the corresponding period (for ED users) were treated as missing data and excluded from the calculation. For example, among the 657 participants included in the baseline analysis, 36 had never initiated PrEP and 621 had already initiated PrEP (529 ED users and 92 daily users). Among the 529 ED users at baseline, only 373 had reported sexual activities in the past 1 month and 458 had sex in the past 3 month, and they were thus included as the denominator when calculating the prevalence of optimal adherence in the past 1-month and 3-months.
3) We have clarified it in the revised Methods section.

(page 9-10, lines 181–187) *‘Adherence to ED regimen was assessed by four items. First, the participants were asked whether they had engaged in sexual activity in the past one and three months, respectively (the 1-month follow-up survey only asked this question for the past one month). Those who answered “no” or reported to have never initiated PrEP in a previous question were treated as missing data for PrEP adherence. Those who answered “yes” were then asked if they had taken PrEP for each sexual encounter in the corresponding time period…….”*

**R3. 16** Line 173: The tone of the question, "Have you ever failed to take PrEP on time and in a correct dosage?" sounds judgmental. I suspect there may be substantial social desirability in the self-report (for both daily and ED dosing), which may need more discussion than a brief mention in the limitations section.

Response: Thanks.

1) The wording “Have you ever failed to take PrEP on time and in a correct dosage?” in our original manuscript was a translation issue. The original question in Chinese aimed to neutrally capture whether participants had any deviations from recommended dosing without implying judgment. To avoid misunderstanding, we have performed a translation and back translation again of the questionnaire (S2 File), and revised the wording of the question to: *“In the past one, did you have any occasions when you did not take PrEP on time or following the recommended dosage?”* (page 10, lines 200–202).
2) We acknowledge that this question together with other sensitive questions might induce social desirability bias to the study. We have elaborated on it in the revised limitation section.

(page 29-30, lines 510–517) ‘*Second, the reliance on self-reported measures to assess PrEP adherence and other socio-demographic and psycho-behavioral variables may introduce recall and social desirability biases. To minimize these biases, we rigorously designed the questionnaire, carefully delivered informed consent, conducted anonymous surveys, and performed quality checks. Nevertheless, the self-reported measures may still lead to an overestimation of PrEP adherence in the study. Future studies using more objective measurements (e.g., smart pill box, ecological momentary assessment of PrEP adherence via digital health platform) are warranted.’*

**R3. 17** Line 177: Was a list of reasons given for possible non-adherence, or were the questions open-ended?

Response: Thanks. In the questionnaires, we provided a list of reasons for non-adherence (e.g., I didn't know the correct way of taking PrEP; I forgot to take PrEP; I don't have access to PrEP in a timely manner; I experience adverse side effects) and an open-ended option (Other, please specify _______). We have modified the statement in the revised Methods section.

(page 11, lines 208–211) *‘In addition, all non-adherent participants were then asked to report reasons for missing doses. A list of reasons (e.g., I didn't know the correct way of taking PrEP; I forgot to take PrEP; I don't have access to PrEP in a timely manner; I experience adverse side effects) together with an open-ended option (Other, please specify) were provided.’*

**R3. 18** Lines 218 and 221: Do the Cronbach alphas refer to the data in this study or from a validation cohort (e.g., in the literature)?

Response: Thanks. We confirm that the Cronbach alphas refer to the data in this study. We have clarified it in the revised Methods section. For example:

(page 12, lines 238–242) *‘PrEP-related stigma was measured by the 11-item Chinese version of HIV PrEP Stigma Scale……. A summative score ranging from 11 to 55 (α in this study = 0.81) was calculated by summing item scores (1 = strongly disagree, 5 = strongly agree), with a higher score indicating greater stigma.’*

**R3. 19** Line 229: It seems like the sample size was large enough for at least univariable regression among daily users. The authors may wish to present that data.

Response: Thank you for your suggestion. We have incorporated a new univariable regression model per your suggestion.

1) We have mentioned it in the revised statistical analysis section

(page 14, lines 285–288) *‘**Due to the small sample size (n = 96) of daily users, a univariable GLMM was fitted to examine the significance of association between each exposure variable and the adherence rate in the past month.’*

2) We have added a supplementary table to show the full results of the regression.

S7 Table. Univariable linear mixed-effects regression results for adherence rate (%) among daily PrEP users

| **Variables** | **Daily users** | | |
| --- | --- | --- | --- |
|  | ***β*** | **95% CI** | ***P*-value** |
| Age | -0.03 | (-0.22, 0.16) | 0.753 |
| Ethnicity (Others vs. Han) | 0.94 | (-2.47, 4.34) | 0.586 |
| Monthly income (>3000 CNY vs. ≤3000 CNY) | -0.67 | (-3.70, 2.37) | 0.665 |
| Marital status (Unmarried/Divorced/Separated/Widowed vs. Married/Living with a Partner) | -3.51 | (-6.30, -0.72) | **0.014** |
| Education (College and above vs. High school or below) | -1.43 | (-5.08, 2.21) | 0.437 |
| Employment status (Employed vs. Students/Unemployed) | 2.87 | (-2.31, 8.06) | 0.278 |
| Knowledge of event-driven regimen (Correct vs. Incorrect) | -0.56 | (-2.28, 1.16) | 0.525 |
| Having multiple homosexual partners in the past three months (Multiple homosexual partners vs. One sex partner) | 1.82 | (-0.36, 3.99) | 0.102 |
| Sexual role in the past three months (Receptive or versatile vs. Insertive) | 0.38 | (-1.67, 2.43) | 0.718 |
| Chemsex in the past three months (Having chemsex vs. No chemsex) | 0.37 | (-1.54, 2.28) | 0.705 |
| Condom use in the past three months (Inconsistent vs. Consistent use) | 0.20 | (-1.76, 2.16) | 0.839 |
| Commercial sex in the past three months (Having commercial sex vs. No commercial sex) | 0.77 | (-1.95, 3.50) | 0.578 |
| Self-efficacy of being adhere to PrEP | 0.64 | (0.05, 1.23) | **0.034** |
| Resilience | -0.19 | (-0.70, 0.32) | 0.457 |
| Depressive symptoms | -0.01 | (-0.17, 0.17) | .0.978 |
| PrEP-related stigma | 0.01 | (-0.12, 0.14) | 0.866 |

3) We briefly presented the results in the revised Results section.

(page 23, lines 397–400) ‘*In the univariable regression analyses using adherence rate as a continuous outcome for daily users (S7 Table), no significant associations were observed for most exposure variables, except for marital status (b = −3.51, 95% CI: −6.30 to −0.72, p = 0.014) and self-efficacy (b = 0.64, 95% CI: 0.05 to 1.23, p = 0.034).’*

4) Discussion of these findings were incorporated in the revised manuscript.

(page 27, lines 456–458)*‘Self-efficacy played as a consistently significant positive predictor of PrEP adherence among both ED and daily users across all the models using different outcome definitions in the study, aligning with previous research findings’*

**R3.20** Were all variables used in the multivariable models? Please clarify and if some were excluded, provide selection process.

Response: Thanks. In the multivariable regression analysis, we included all the exposure variables of interest in the model. We have clarified it in the revised Methods section.

(page 14, lines 272–285) *‘Second, regression analyses were performed to examine the statistical significance of crude and independent associations between all the exposure variables of interest (socio-demographics, psycho-behavior variables) and the adherence outcomes. For ED users, three regression models were fitted for the three adherence outcomes: (1) model 1 included a univariate generalized linear mixed model (GLMM) with a logit link and random intercept to evaluate the statistical significance of crude association between each exposure variable and the past three-month adherence, as well as a multivariable GLMM including all exposure variables of interest in order to evaluate the significance of their independent associations with the outcome after controlling potential confounders (covariates); (2) model 2 were similar univariate and multivariable GLMM for the past one-month adherence outcome; (3) model 3 included a univariate and a multivariable logistic regression model for the consistent adherence outcome, in which baseline socio-demographics and averaged time-varying values at 3- and 6-months for other exposure variables were included as independent variables.…….’*

**Results**

**R3. 21** Figure 1 should separate out the reasons for ineligibility and refusal to participate in the study. Also, is a cascade-style of presenting participation in the cohort appropriate? For example, did any participants miss a 1-month follow-up survey but then complete a 3- or 6-month follow-up survey?

Response: Thanks for your comment.

1) In the revised Figure 1, we have separated out reasons for ineligibility and removed the cascade-style which is actually in appropriate, as the participants who were lost to follow-up at 1-mon follow-up would still be contacted and invited to join the 3- and 6-follow-up surveys.

**Fig 1. Flowchart of the RECOPE study**

2) We further added a supplementary S1 Table to show the patterns of loss to follow-up, and found that 27.5% of the cohort participants had at least one loss to follow-up/non-response to the follow-up surveys.

S1 Table: Patterns of loss to follow-up of the study

| **Patterns** | **n** | **% (n/657)** |
| --- | --- | --- |
| Lost to follow-up (survey non-responses) for three times | 47 | 7.1% |
| Lost to follow-up (survey non-responses) for two times | 56 | 8.5% |
| Lost to follow-up (survey non-responses) for one time | 78 | 11.9% |
| Total | 181 | 27.5% |

**R3.22:** Table 2 was difficult to read given its size. I would reformat it to make it easier to digest. I would also present depression as none, mild, or moderate/severe. Reporting mild to severe as one category is hard to interpret.

Response: Thanks. We have revised Table 2 per your suggestion and comments from other reviewers.

**Table 2.** Distribution of sexual behaviors, PrEP-related variables, and psychosocial variables in the RECOPE study

| **Variables** | **Total** | | | | ***P ^d^*** |  | **ED regimen** | | | | ***P ^d^*** |  | **Daily regimen** | | | | ***P ^d^*** |  | **Continuous Sexually active^a^** |
| --- | --- | --- | --- | --- | --- | --- | --- | --- | --- | --- | --- | --- | --- | --- | --- | --- | --- | --- | --- |
|  | **Baseline**  **(N=657)** | **1 month**  **(N=582)** | **3 month**  **(N=539)** | **6 month**  **(N=520)** |  |  | **Baseline**  **(n=529)** | **1 month**  **(n=458)** | **3 month**  **(n=428)** | **6 month**  **(n=422)** |  |  | **Baseline**  **(n=92)** | **1 month**  **(n=102)** | **3 month**  **(n=98)** | **6 month**  **(n=88)** |  |  |  |
| **Sexually active** |  |  |  |  | **<0.001** |  |  |  |  |  | **<0.001** |  |  |  |  |  | 0.476 |  |  |
| Yes | 578 (88.0) | 454 (78.4) | 481 (89.2) | 470 (90.4) |  |  | 458 (86.6) | 339 (74.0) | 377 (88.1) | 374 (88.8) |  |  | 88 (95.7) | 100 (98.0) | 94 (95.9) | 88 (98.9) |  |  | 310 (100.0) |
| No | 79 (12.0) | 128 (21.6) | 58 (10.8) | 50 (9.6) |  |  | 71 (13.4) | 119 (26.0) | 51 (11.9) | 47 (11.2) |  |  | 4 (4.3) | 2 (2.0) | 4 (4.1) | 1 (1.1) |  |  | 0 (0.0) |
| **Chemsex^b^** |  |  |  |  | 0.564 |  |  |  |  |  | 0.338 |  |  |  |  |  | 0.803 |  |  |
| Yes | 247 (42.7) | 182 (40.1) | 202 (42.0) | 183 (38.9) |  |  | 202 (44.1) | 134 (39.5) | 157 (41.6) | 143 (38.2) |  |  | 35 (39.8) | 44 (44.0) | 44 (46.8) | 37 (42.0) |  |  | 160 (51.6) |
| No | 331 (57.3) | 272 (59.9) | 279 (58.0) | 287 (61.1) |  |  | 256 (55.9) | 205 (60.5) | 220 (58.4) | 231 (61.8) |  |  | 53 (60.2) | 56 (56.0) | 50 (53.2) | 51 (58.0) |  |  | 150 (48.4) |
| **Condom use^b^** |  |  |  |  | 0.090 |  |  |  |  |  | 0.303 |  |  |  |  |  | 0.790 |  |  |
| Inconsistent | 218 (37.7) | 201 (44.3) | 210 (43.7) | 206 (43.8) |  |  | 164 (35.8) | 141 (41.6) | 154 (40.8) | 151 (40.4) |  |  | 46 (52.3) | 56 (56.0) | 55 (58.5) | 52 (59.1) |  |  | 144 (46.5) |
| Consistent | 360 (62.3) | 253 (55.7) | 271 (56.3) | 264 (56.2) |  |  | 294 (64.2) | 198 (58.4) | 223 (59.2) | 223 (59.6) |  |  | 42 (47.7) | 44 (44.0) | 39 (41.5) | 36 (40.9) |  |  | 166 (53.5) |
| **Sexual partner^b^** |  |  |  |  | **<0.001** |  |  |  |  |  | **<0.001** |  |  |  |  |  | 0.240 |  |  |
| 1 | 110 (19.0) | 139 (30.6) | 125 (26.0) | 132 (28.1) |  |  | 84 (18.3) | 108 (31.9) | 109 (28.9) | 115 (30.7) |  |  | 17 (19.3) | 25 (25.0) | 13 (13.8) | 15 (17.0) |  |  | 53 (17.1) |
| >1 | 468 (81.0) | 315 (69.4) | 356 (74.0) | 338 (71.9) |  |  | 374 (81.7) | 231 (68.1) | 268 (71.1) | 259 (69.3) |  |  | 71 (80.7) | 75 (75.0) | 81 (86.2) | 73 (83.0) |  |  | 257 (82.9) |
| **Sexual role^b^** |  |  |  |  | 0.649 |  |  |  |  |  | 0.650 |  |  |  |  |  | 0.600 |  |  |
| Receptive or versatile | 267 (46.2) | 220 (48.5) | 222 (46.2) | 208 (44.3) |  |  | 222 (48.5) | 172 (50.7) | 176 (46.7) | 174 (46.5) |  |  | 34 (38.6) | 44 (44.0) | 44 (46.8) | 34 (38.6) |  |  | 155 (50.0) |
| Insertive | 311 (53.8) | 234 (51.5) | 259 (53.8) | 262 (55.7) |  |  | 236 (51.5) | 167 (49.3) | 201 (53.3) | 200 (53.5) |  |  | 54 (61.4) | 56 (56.0) | 50 (53.2) | 54 (61.4) |  |  | 155 (50.0) |
| **Commercial sex^b^** |  |  |  |  | 0.676 |  |  |  |  |  | 0.673 |  |  |  |  |  | 0.892 |  |  |
| Yes | 50 (8.7) | 36 (7.9) | 35 (7.3) | 44 (9.4) |  |  | 39 (8.5) | 27 (8.0) | 24 (6.4) | 31 (8.3) |  |  | 9 (10.2) | 9 (9.0) | 10 (10.6) | 11 (12.5) |  |  | 34 (11.0) |
| No | 528 (91.3) | 418 (92.1) | 446 (92.7) | 426 (90.6) |  |  | 419 (91.5) | 312 (92.0) | 353 (93.6) | 343 (91.7) |  |  | 79 (89.8) | 91 (91.0) | 84 (89.4) | 77 (87.5) |  |  | 276 (89.0) |
| **PrEP knowledge**^c^ |  |  |  |  | **0.014** |  |  |  |  |  | **0.002** |  |  |  |  |  | 0.590 |  |  |
| Correct | 455 (69.3) | 416 (71.5) | 410 (76.1) | 396 (76.2) |  |  | 390 (73.7) | 356 (77.7) | 356 (83.2) | 341 (81.0) |  |  | 42 (45.7) | 49 (48.0) | 46 (46.9) | 49 (55.1) |  |  | 233 (75.2) |
| In correct | 202 (30.7) | 166 (28.5) | 129 (23.9) | 124 (23.8) |  |  | 139 (26.3) | 102 (22.3) | 72 (16.8) | 80 (19.0) |  |  | 50 (54.3) | 53 (52.0) | 52 (53.1) | 40 (44.9) |  |  | 77 (24.8) |
| **Self-efficacy of PrEP adherence** | 3.9±1.8 | 4.2±1.8 | 4.4±1.8 | 4.3±1.9 | **<0.001** |  | 3.8±1.9 | 4.0±1.8 | 4.3±1.8 | 4.1±1.9 | **<0.001** |  | 4.6±1.6 | 5.1±1.4 | 5.1±1.3 | 5.1±1.6 | **0.041** |  | 4.1±1.7 |
| **PrEP-related stigma** | 25.1±6.9 | - | 25.0±7.1 | 24.9±6.6 | 0.084 |  | 25.3±6.7 | - | 25.4±6.9 | 24.7±6.7 | 0.275 |  | 23.2±7.3 | - | 23.3±7.4 | 25.7±6.6 | **0.011** |  | 25.1±4.6 |
| **Depressive symptoms** | 7.2±5.3 | - | 7.4±5.3 | 7.2±5.2 | 0.267 |  | 7.1±5.3 | - | 7.3±5.3 | 7.0±5.0 | 0.283 |  | 7.4±5.4 | - | 7.5±5.7 | 8.0±5.7 | 0.477 |  | 7.2±3.7 |
| None | 215 (32.7) | - | 172 (31.9) | 155 (29.8) |  |  | 175 (33.1) | - | 135 (25.3) | 129 (30.6) |  |  | 29 (31.5) | - | 30 (32.6) | 23 (25.8) |  |  | 65 (21.0) |
| Mild | 280 (42.6) | - | 233 (43.2) | 249 (47.9) |  |  | 223 (42.2) | - | 196 (37.1) | 207 (49.2) |  |  | 40 (43.5) | - | 27 (29.3) | 40 (44.9) |  |  | 103 (49.4) |
| Moderate | 97 (14.8) | - | 73 (13.5) | 67 (12.9) |  |  | 79 (14.9) | - | 61 (11.5) | 50 (11.9) |  |  | 13 (14.1) | - | 9 (9.8) | 14 (15.7) |  |  | 56 (18.1) |
| Moderate severe | 40 (6.1) | - | 43 (8.0) | 34 (6.5) |  |  | 32 (6.0) | - | 36 (6.8) | 25 (5.9) |  |  | 6 (6.5) | - | 5 (5.4) | 8 (9.0) |  |  | 20 (6.5) |
| Severe | 25 (3.8) | - | 18 (3.3) | 15 (2.9) |  |  | 20 (3.8) | - | 16 (30) | 10 (2.4) |  |  | 4 (4.3) | - | 2 (2.2) | 4 (4.5) |  |  | 16 (5.2) |
| **Resilience** | 5.2±1.9 | - | 5.7±1.7 | 5.8±1.6 | **<0.001** |  | 5.2±1.9 | - | 5.7±1.7 | 5.7±1.7 | **<0.001** |  | 5.0±2.1 | - | 5.7±1.7 | 6.0±1.6 | **<0.001** |  | 5.7±1.2 |

^a^ Chemsex, and commercial sex were classified as “yes” if participants reported engaging in these behaviors at least once across the two follow-up assessments. If participants reported Having multiple homosexual partners at least once during the two follow-ups, they were classified as “> 1”. If participants reported Inconsistent condom use at least once during the two follow-ups, they were classified as “Inconsistent”. If participants reported their sexual role as receptive or versatile at least once during the two follow-ups, they were classified as “receptive or versatile”. Self-efficacy for being adhere to PrEP, resilience, depressive symptoms, and PrEP-related stigma in the last column were assessed using the mean scores from the two follow-up assessments.

^b^ For chemsex, condom use, sexual partner, sexual role, and commercial sex, the total sample for these variables are those who have reported being sexually active for the past study duration.

^c^ PrEP-related knowledge was assessed by asking participants to identify correct dosing instructions based on their regimen (daily or ED). Only those who selected all correct options were coded as having correct PrEP adherence knowledge.

^d^ The Chi-square test was used for categorical variables and t-test was used for continuous variables.

**R3.23:** I suggest the authors give the actual adherence rates for daily PrEP, not just the dichotomized data. A linear approach would also enable predictive analysis (see my comment above).

Response: Thanks. As mentioned in the response to your previous comments R3.6 and R3.19, we have added the actual adherence rates and a univariable regression model on it in the revised manuscript.

**R3.24:** The authors may want to look at ED PrEP use with partner types (e.g., main vs casual) and compare the findings to similar data from elsewhere (e.g., Jongen et al JIAS 2021).

Response: We thank the reviewer for this suggestion. However, we are unable to look at ED PrEP use with partner types in this study, as we did not have detail data on PrEP use by partner types. We only have data on the number of sexual partners and whether they had commercial sex in the past 1 or 3 months. We hope to collect this interesting data in our future study.

**R3.25** Table 3. I would give the actual p-values for each variable rather than categorize them as <0.05, <0.01, or <0.001.

Response: Thanks. We have modified Table 3 accordingly.

**Table 3** Factors associated with optimal PrEP adherence in the past three month based on baseline and 3- and 6-month follow-up data among ED users.

| **Variables** | **Model 1 on optimal adherence in past three months (Univariate analysis)** | | |  | **Model 1 on optimal adherence in past three months (Multivariable analysis)** | | |
| --- | --- | --- | --- | --- | --- | --- | --- |
|  | **OR** | **95% CI** | ***P*** |  | **aOR** | **95% CI** | ***P*** |
| Age | 1.01 | (0.98, 1.03) | 0.728 |  | 1.02 | (0.98, 1.06) | 0.239 |
| Ethnicity (Others vs. Han) | 1.42 | (0.99, 2.03) | 0.860 |  | 1.01 | (0.41, 2.49) | 0.984 |
| Monthly income (>3000 CNY vs. ≤3000 CNY) | 0.89 | (0.54, 1.46) | 0.317 |  | 0.67 | (0.41, 2.49) | 0.217 |
| Marital status (Unmarried/Divorced/Separated/Widowed vs. Married/Living with a Partner) | 0.67 | (0.35, 1.27) | 0.157 |  | 0.75 | (0.38, 1.46) | 0.386 |
| Education (College and above vs. High school or below) | 0.71 | (0.49, 1.01) | 0.685 |  | 1.19 | (0.42, 3.39) | 0.999 |
| Employment status (Employed vs. Students/Unemployed) | 0.95 | (0.48, 1.88) | 0.847 |  | 1.04 | (0.56, 1.92) | 0.889 |
| Knowledge of event-driven regimen (Correct vs. Incorrect) | 1.44 | (0.85, 2.44) | 0.206 |  | 1.62 | (0.98, 2.69) | 0.055 |
| Having multiple homosexual partners in the past three months (Multiple homosexual partners vs. One sex partner) | 0.35 | (0.22, 0.56) | **<0.001** |  | 0.50 | (0.28, 0.88) | **0.015** |
| Sexual role in the past three months (Receptive or versatile vs. Insertive) | 1.39 | (0.85, 2.26) | 0.145 |  | 1.56 | (1.01, 2.41) | **0.044** |
| Chemsex in the past three months (Having chemsex vs. No chemsex) | 0.40 | (0.25, 0.64) | **<0.001** |  | 0.72 | (0.46, 1.12) | 0.130 |
| Condom use in the past three months (Inconsistent vs. Consistent use) | 0.92 | (0.59, 1.43) | 0.829 |  | 0.96 | (0.61, 1.49) | 0.774 |
| Commercial sex in the past three months (Having commercial sex vs. No commercial sex) | 0.56 | (0.24, 1.26) | 0.210 |  | 1.73 | (0.81, 3.70) | 0.163 |
| Self-efficacy of being adhere to PrEP | 1.88 | (1.65, 2.15) | **<0.001** |  | 1.51 | (1.31, 1.75) | **<0.001** |
| Resilience | 1.02 | (0.91, 1.14) | 0.775 |  | 0.95 | (0.84, 1.07) | 0.272 |
| Depressive symptoms | 0.97 | (0.93, 1.01) | 0.140 |  | 0.97 | (0.93, 1.02) | 0.256 |
| PrEP-related stigma | 0.98 | (0.95, 1.01) | 0.127 |  | 0.96 | (0.93, 0.99) | **0.039** |

**R3.26** Line 355: The high adherence among daily users could be due to social desirability bias (see my comment above). If true, however, it seems the conclusion may be to expand access to daily PrEP, not further educational programs, etc (although I agree those are still needed for people not accessing PrEP or adhering well).

Response: Thanks. We have modified the statements accordingly per your suggestion.

(page 26-27, lines 446–451)‘*……A possible reason is that in these previous trials, participants obtained PrEP free of charge, whereas in the present study, the participants were real-world clients who perceived themselves as individuals at high risk of HIV and bought the medication out-of-pocket. As a result, they may take medication adherence more seriously. Another possible reason for the high adherence among the daily PrEP users is the social desirability bias introduced by self-administered survey questionnaires……’*

**R3.27:** The authors state "urgent needs" at least three times. I would be selective in prioritizing truly urgent action.

Response: Thanks. We have carefully reviewed the manuscript and revised the phrasing to ensure that the use of “urgent” is reserved only for the most critical public health implications.

**R3.28** Line 413: Can the authors explain what they mean by "intelligent monitoring approaches"?

Response: Thanks. We have explained it in the revised manuscript accordingly.

(page 29-30, lines 510–517) ‘*Second, the reliance on self-reported measures to assess PrEP adherence and other socio-demographic and psycho-behavioral variables may introduce recall and social desirability biases……. Future studies using more objective measurements (e.g., smart pill box, ecological momentary assessment of PrEP adherence via digital health platform) are warranted.’*

**R3.29:** As noted above, I suggest the authors comment on adherence in relation to potential variable need for PrEP use over time.

Response: Thanks for your suggestion. As mentioned in the responses to comment R3.2 and R3.8, we did not consider the align of PrEP use with potential exposure to HIV in the study for several reasons (e.g., self-reported measures on condom use and other preventive measures might be biased, the current official guidelines of PrEP do not mention the adherence is unnecessary if using condoms, and the majority of the study sample were ED users). We have suggested future research to better address this issue in the revised Discussion section.

(page 26, lines 431–440) *‘……Additionally, the definition of optimal adherence used in this study (complete compliance with the ‘2-1-1’ dosing schedule for each sex activity in the past one/three months) was stringent and did not consider actual risk exposure to HIV. For example, the most commonly reported reason for non-adherence was use of condoms, which infers that some non-adhere ED users might still be able to protect themselves from HIV infection due to correct condom use. However, since self-reported measures of condom use in the study might introduce recall bias and social desirability bias, it is difficult to evaluate the real exposure risk of HIV in the study. Future studies may consider to calculate prevention-effective adherence in which PrEP use is aligned with potential risk exposure [31], if more rigorous measurement of condoms use and other HIV risk exposure variables are used.’*

**Reference:**

[31] Haberer JE, Bangsberg DR, Baeten JM, Curran K, Koechlin F, Amico KR, et al. Defining success with HIV pre-exposure prophylaxis: a prevention-effective adherence paradigm. AIDS 2015;29:1277.

*********************************************************************

We thank the Editor and Reviewer again for reviewing the manuscript. Please do not hesitate to contact us if you had further comments.
