# Supplementary material for: Medication adherence and its associated factors among oral pre-exposure prophylaxis (PrEP) users in China: The Real-world E-consumer Cohort of PrEP study
Source: PLoS Med. 2026 Feb 26;23(2):e1004733. doi: 10.1371/journal.pmed.1004733 (PMC12944781; doi:10.1371/journal.pmed.1004733)
Supplement: Attachment — Submitted filename: response letter1227-final.docx [file pmed.1004733.s017.docx]

Dear Editor and Reviewers,

Thank you very much for your review of the manuscript PMEDICINE-D-25-02683R1. We have further considered and addressed your specific comments carefully and provided a point-by-point response below. We believe the manuscript is much more informative and stronger now.

*********************************************************************

**Editor**

E1.1. Many thanks for submitting your manuscript "Medication adherence and its associated factors among oral PrEP users in China: Main results of the real-world e-consumer cohort of pre-exposure prophylaxis (RECOPE)" (PMEDICINE-D-25-02683R2) to PLOS Medicine. The paper has been reviewed by subject experts and a statistician; their comments are included below and can also be accessed here:

As you will see, the reviewers find the revised manuscript improved and addresses most of their concerns. The remaining comments of reviewers 2, 3 and the academic editor are appended below. Please note that we require a response and/or qualification in the text regarding the statistical reviewer’s concerns, more in depth discussion of potential selection bias and of the role of other HIV prevention strategies, and their implications of these issues for your results and conclusions. Please also ensure that your conclusions reflect event driven PrEP adherence. After discussing the paper with the editorial team and an academic editor with relevant expertise, I’m pleased to invite you to revise the paper in response to the reviewers’ comments. We plan to send the revised paper to some or all of the original reviewers, and we cannot provide any guarantees at this stage regarding publication.

Response: We sincerely thank the Editor and the editorial team for providing us the opportunity to revise the paper. We have carefully reviewed all the comments and have addressed each of them in the revised manuscript.

1) Regarding the statistical reviewer’s concern, we have clarified the purpose and interpretation of the multivariable models and modified the corresponding description in the Methods and Discussion to avoid causal interpretation.
2) We have elaborated our discussion of potential selection bias to provide a clearer explanation of how self-selection into study participation and continued follow-up may have influenced the adherence estimates, and we have now explicitly described the possible direction of this bias.
3) We have substantially strengthened the discussion of the role of alternative HIV prevention strategies, as it had been reported as the top reason for non-adherence among ED users in the study. The revised text now recognizes that in addition to PrEP, other prevention strategies such as condom use can also contribute to HIV prevention among PrEP users.
4) We also revised the Conclusion to ensure that it accurately reflects our findings by specifying that low medication adherence was found among ED PrEP users, while adherence to the daily regimen remained high.

E1.2. Please include a completed CROSS checklist with your revised manuscript (https://www.equator-network.org/reporting-guidelines/a-consensus-based-checklist-for-reporting-of-survey-studies-cross/).

Response: We have completed the CROSS checklist and submitted it as a supplementary file with the revised manuscript.

E1.3. In addition to these revisions, you may need to complete some formatting changes, which you will receive in a follow up email. A member of our team will be in touch with a set of requests shortly. If you do not receive a separate email within a few days, please assume that checks have been completed, and no additional changes are required.

Response: Thanks. Since we haven’t received the separate email, thus we assume no additional changes are required.

E1.4. When you upload your revision, please include a point-by-point response that addresses all of the reviewer and editorial points, indicating the changes made in the manuscript and either an excerpt of the revised text or the location (eg: page and line number) where each change can be found. Please also be sure to check the general editorial comments at the end of this letter and include these in your point-by-point response. When you resubmit your paper, please include a clean version of the paper as the main article file and a version with changes tracked as a marked-up manuscript. It may also be helpful to check the guidelines for revised papers at http://journals.plos.org/plosmedicine/s/revising-your-manuscript for any that apply to your paper.

Response: As requested, we have submitted a clean version of the manuscript, a marked-up version with tracked changes, and a complete point-by-point response indicating the location of each revision by page and line number.

*********************************************************************

**Academic editor**

E2.1. I continue to feel that the issue of sampling selection bias is under-emphasized, in particular the concern that respondents were more likely to be enthusiastic about PrEP and adherent to it than non-respondents. The statement "It is possible that those with better PrEP adherence might be more willing to participate in the study" is not sufficient, in my view. Please consider in more depth how such a bias would affect their results. If respondents represent the best adherers, then true adherence in the overall population would likely be lower than reported, not higher.

Response: We thank the academic editor for the comment. We agree that the selection bias needs further elaboration. In the revised limitation section, we have discussed in more detail how the selection bias would affect the results of the study per the editor’s suggestions.

**Page29, line506-514** “*First, the convenience sampling method used in the study might introduce selection bias. The respondents of the study might be more enthusiastic about PrEP and HIV prevention and thus had better medication adherence than the non-respondents. In other words, those with better PrEP adherence might be more willing to participate in the study. Therefore, the PrEP adherence level reported in this study might be higher than that of the overall real-world users who bought PrEP online in China. The PrEP adherence might be overestimated in the study. But as no information were available for the non-respondents, the exact direction and magnitude of selection bias could not be gauged.”*

E2.2. The authors are too dismissive of alternative prevention strategies, such as condoms. The reviewer who mentioned that there are multiple ways to prevent HIV infection and that risk has to be considered holistically was correct. The manuscript dismisses the finding that the "top self reported reasons for non-adherence included ‘I used other HIV prevention’." The rest of that paragraph goes on to dismiss self-reported condom use as unreliable, while fully accepting self-reported PrEP use. This seems particularly problematic for ED PrEP, where condom use seems like a reasonable substitute for PrEP. I would suggest that the authors consider the potential trading off of prevention methods as a valid reason for not using ED Prep, rather than an example of non-adherence, and think through the implications accordingly.

Response: Thank you for this insightful comment. After further careful consideration, we now agree with the editor and the reviewer that our earlier statements are too dismissive of alternative prevention strategies, such as condom use. In the revised manuscript, we acknowledged that other HIV prevention strategies such as using condoms correctly and consistently is a reasonable substitute for PrEP and have considered the potential trading off of prevention methods as a valid reason for not using ED PrEP.

**Page26-26, line430-449** “*Some possible explanations for the suboptimal adherence among ED users in the RECOPE study include potential gaps in instruction and counseling on medication use among e-consumers (indicated by the findings that about one quarter of the ED users did not correctly answer all the knowledge questions), use of other promising HIV prevention strategies (indicated by the finding that the top self-reported reasons for non-adherence included ‘I used other HIV prevention’ ), the complexity of the ED regimen, lack of a systematic reminder mechanism, and difficulty in predicting sexual activity. The findings imply that future interventions on PrEP adherence should address these important issues. To be noted, the most commonly reported reason for non-adherence among ED users in the study was use of other prevention strategies such as condoms, which infers that some non-adhere ED users might still be able to protect themselves from HIV infection. Condoms, when used correctly and consistently, are safe and highly effective in preventing transmission of most sexually transmitted infections, including HIV [32], and can be considered as a reasonable substitute of PrEP and reduce risk of HIV infection. Future studies may consider to calculate prevention-effective adherence in which PrEP use is aligned with potential risk exposure [33]. Meanwhile, the finding indicates that in order to fully protect people from HIV, it is also important to integrate health education of other HIV prevention strategies (e.g., correct and consistent use of condom, partner viral suppression [U=U]) into future PrEP services and interventions.”*

*References:*

*[32] WHO. Condoms [Internet]. 2025 [cited 2025 Oct 15]. Available from:* <https://www.who.int/news-room/questions-and-answers/item/condoms>

*[33] Haberer JE, Bangsberg DR, Baeten JM, Curran K, Koechlin F, Amico KR, et al. Defining success with HIV pre-exposure prophylaxis: a prevention-effective adherence paradigm. AIDS 2015;29:1277. https://doi.org/10.1097/QAD.0000000000000647.*

E2.3. The first sentence of the conclusion is not accurate, as adherence to daily oral PrEP was rather high. The results need to be consistently limited to ED PrEP, to avoid a very misleading takeaway message.

Response: Thanks. We have revised the first sentence of the Conclusion to clarify that low adherence was observed among ED PrEP users , while adherence to the daily regimen remained high.

**Page31, line555-562** *“The RECOPE study revealed the medication adherence of ED PrEP users was suboptimal, while that of the daily PrEP users was relatively high. Since ED users accounted for the majority of real-world e-consumers of PrEP in China, the low medication adherence may be detrimental to the progress of HIV prevention and control in the country and the globe. Future targeted interventions are suggested to enhance comprehension of medication instructions, strengthen self-efficacy, and mitigate PrEP-related stigma. Special attention should be given to PrEP users who engage in high-risk sexual behaviors and have a younger age.”* *********************************************************************

**Reviewer #2**

R2.1. Thanks for the revised manuscript and responses to my original review. The updates to the methods and results have clarified most of the queries from my original review.

Response: We thank the reviewer for the original and further review. We have addressed the remaining comments point-by-point below and revised the manuscript accordingly.

R2.2. My main outstanding issue is the interpretation of odds ratios from the multivariable model. It is reasonable to want to understand which covariates are associated with adherence without confounding, but the output (coefficients and p-values) of multivariable model with all covariates included assumes a very particular causal model where none of the covariates are mediators of other others. The approach used is more like a prognostic factor study (e.g. doi:10.1371/journal.pmed.1001380) and if this the aim, regression models this should be made clear. Otherwise, the causal model needs to be proposed to ensure that direct effects are not confused with total effects in the model output.

Response: Thanks for this comment. We agree with the reviewer that the aim of our multivariable models is more align with the prognostic factor study. We aimed to examine whether the exposure variables of interest would be statistically associated with the outcome variable after controlling for potential confounders, so as to identify targets for future interventions on PrEP adherence, but not aim to build a causal model to make causal analysis. In the revised manuscript, we have further revised the Methods section to clarify that the multivariable regression analyses were conducted to identify prognostic factors of the outcome variables, so as to inform future intervention design.

**Page14, line276-280** *“Second, regression analyses were performed to identify prognostic factors of PrEP adherence by examining statistical significance of crude and adjusted associations between the exposure variables (socio-demographic and psycho-behavioral measures) and the adherence outcomes. The prognosis factor strategy is useful for helping identify targets and enhancing design of future interventions on PrEP adherence [26]. ”*

*Reference:*

*[26] Riley, R. D., Hayden, J. A., Steyerberg, E. W., Moons, K. G., Abrams, K., Kyzas, P. A., Malats, N., Briggs, A., Schroter, S., Altman, D. G., Hemingway, H., & PROGRESS Group (2013). Prognosis Research Strategy (PROGRESS) 2: prognostic factor research. PLoS medicine, 10(2), e1001380.*

R2.3. L324. It would be more accurate to say that ‘there was no evidence of a difference (p>0.05) in the distributions of socio-demographics overall and by regimen throughout the survey waves.

Response: Thanks. We have revised the sentence to more accurately reflect the interpretation of the p-values accordingly.

**Page16, line331-333** *“There was no evidence of a difference (all p > 0.05) in the distributions of socio-demographic characteristics, either overall or by regimen, across the survey waves.”*

R2.4. L341. I would rephrase this as "There was good evidence (p<0.05) of a difference in sexual activity status, …."

Response: Thanks. We have rephrased the statement accordingly.

**Page19, line349-353** *“There was good evidence (p < 0.05) of a difference in sexual activity status, number of sexual partners, PrEP knowledge, self-efficacy for PrEP adherence, and resilience scores across survey waves in the total sample and among ED users. Among daily users, there was also good evidence (p < 0.05) of differences across waves in self-efficacy for PrEP adherence, PrEP-related stigma, and resilience.”*

*********************************************************************

**Reviewer #3**

R3.1. I appreciate the opportunity to re-review this paper, which is much improved. I have the following remaining concerns.

Response: We thank the reviewer for re-reviewing our revised manuscript. We have further addressed your comments below and revised the manuscript accordingly to the suggestions.

R3.2. Abstract- When reporting percentages, I would provide the actual numbers as well. Specifically, when presenting the prevalence of optimal adherence, it is not clear which denominators were used (e.g., 680 eligible, 657 completing baseline survey, or the number responding at each time point). Alternatively, the authors could clarify the denominator in the text.

Response: Thanks. In the revised abstract, we have provided all the actual numbers especially the denominators of the prevalence. We have clarified the actual numbers in the main text as well in the revised Results section.

**Page2-3, line33-42** *“**Of 877 individuals invited, 680 were eligible and 657 completed the baseline survey (response rate 96.6%). The follow-up response rate of the 1-, 3-, and 6-months visit was 90.1% (592/657), 82.6% (543/657), and 80.1% (526/657), respectively. Among the 621 participants who had initiated PrEP at baseline, 529 (85.2%) used the ED regimen, 92 (14.8%) used the daily regimen. Among ED users, the prevalence of optimal adherence at the four timepoints was 41.3%* *(154/373), 43.1%* *(146/339), 45.3%* *(139/307), and 52.6%* *(160/304), respectively. Among daily users, the prevalence of optimal adherence was 83.7% (77/92), 81.4% (83/102), 87.8% (86/98), 84.3% (75/89) at each survey wave, respectively. Within this group, the mean adherence rates in the past one month ranged from 97.1% to 98.7% across waves.”*

R3.3. Fig 1 flowchart- I appreciate the revisions, but I would add a footnote to the figure indicating that some participants completed the survey at different time points and refer to the S1 Table. To this point, in the S1 Table, I would reserve use of the term "lost to follow-up" for participants who could no longer be reached in the study. I believe the authors could substitute "missing" for each data time point.

Response: Thanks. We have added a footnote to Figure 1 noting that some participants completed surveys at different time points and directing readers to S1 Table. In S1 Table, we have replaced the term “lost to follow-up” with “missing survey responses”.

**Page16, line321-322** *Note: Some participants completed surveys at different time points; detailed patterns of missing responses are provided in S1 Table.*

**S1 Table****.** Patterns of Missing Survey Responses Across Study Visits

| **Patterns** | **n** | **% (n/657)** |
| --- | --- | --- |
| Missing survey responses at three waves | 47 | 7.1% |
| Missing survey responses at two waves | 56 | 8.5% |
| Missing survey responses at one wave | 78 | 11.9% |
| Total | 181 | 27.5% |

R3.4. I respect the authors’ decision not to incorporate the concept of prevention-effective adherence, and I appreciate the suggestion of considering it in future analysis. However, the rationale that use of "other HIV prevention" may not be correct seems paternalistic and inconsistent. While indeed, real-world use of condoms may not be effective, participants reporting "other HIV prevention" may have done so because of confidence in their use of condoms. They may have also been referring to U=U, which is well acknowledged as effective (i.e., the HPTN 052 study). The authors accept the accuracy of other forms of self-report (e.g., self-reported sex for determining baseline adherence... see R3.15). Additionally, the WHO recommendation for using condoms with PrEP (cited by the authors) is primarily based on the potential for other STIs.

Response: Thank you for this insightful comment. After further careful consideration, we now agree with the reviewer that our earlier statements are too dismissive of alternative prevention strategies, such as condom use. In the revised manuscript, we have acknowledged that other HIV prevention strategies such as using condoms correctly and consistently is a reasonable substitute for PrEP and have considered the potential trading off of prevention methods as a valid reason for not using ED PrEP.

**Page25-26, line430-449 “***Some possible explanations for the suboptimal adherence among ED users in the RECOPE study include potential gaps in instruction and counseling on medication use among e-consumers (indicated by the findings that about one quarter of the ED users did not correctly answer all the knowledge questions), use of other promising HIV prevention strategies (indicated by the finding that the top self-reported reasons for non-adherence included ‘I used other HIV prevention’ ), the complexity of the ED regimen, lack of a systematic reminder mechanism, and difficulty in predicting sexual activity. The findings imply that future interventions on PrEP adherence should address these important issues. To be noted, the most commonly reported reason for non-adherence among ED users in the study was use of other prevention strategies such as condoms, which infers that some non-adhere ED users might still be able to protect themselves from HIV infection. Condoms, when used correctly and consistently, are safe and highly effective in preventing transmission of most sexually transmitted infections, including HIV [32], and can be considered as a reasonable substitute of PrEP and reduce risk of HIV infection. Future studies may consider to calculate prevention-effective adherence in which PrEP use is aligned with potential risk exposure [33]. Meanwhile, the finding indicates that in order to fully protect people from HIV, it is also important to integrate health education of other HIV prevention strategies (e.g., correct and consistent use of condom, partner viral suppression [U=U]) into future PrEP services and interventions.”*

*References:*

*[32] WHO. Condoms [Internet]. 2025 [cited 2025 Oct 15]. Available from:* [*https://www.who.int/news-room/questions-and-answers/item/condoms*](https://www.who.int/news-room/questions-and-answers/item/condoms)

*[33] Haberer JE, Bangsberg DR, Baeten JM, Curran K, Koechlin F, Amico KR, et al. Defining success with HIV pre-exposure prophylaxis: a prevention-effective adherence paradigm. AIDS 2015;29:1277. https://doi.org/10.1097/QAD.0000000000000647.*

R3.5. R3.26 (pg 70) The high adherence to daily PrEP seems like it should be considered as a recommended for individuals who might be interested in it. At a minimum, the conclusion in the main paper that PrEP adherence was low should be qualified to low event driven adherence (as it is in the Abstract).

Response: Thanks. We have revised the first sentence of the Conclusion to clarify that low adherence was observed among ED PrEP users, while adherence to the daily regimen remained high.

**Page31, line555-562** *“The RECOPE study revealed the medication adherence of ED PrEP users was suboptimal, while that of the daily PrEP users was relatively high. Since ED users accounted for the majority of real-world e-consumers of PrEP in China, the low medication adherence may be detrimental to the progress of HIV prevention and control in the country and the globe. Future targeted interventions are suggested to enhance comprehension of medication instructions, strengthen self-efficacy, and mitigate PrEP-related stigma. Special attention should be given to PrEP users who engage in high-risk sexual behaviors and have a younger age.”*

*********************************************************************

GENERAL EDITORIAL REQUESTS

* At this stage, we ask that you include a short, non-technical Author Summary of your research to make findings accessible to a wide audience that includes both scientists and non-scientists. The Author Summary should immediately follow the Abstract in your revised manuscript. This text is subject to editorial change and should be distinct from the scientific abstract. Ideally each sub-heading should contain 2-3 single sentence, concise bullet points containing the most salient points from your study. In the final bullet point of ‘What Do These Findings Mean?’ Please include the main limitations of the study in non-technical language.

Response: Please see the Author Summary below:

- Why was this study done?
  - Oral HIV pre-exposure prophylaxis (PrEP) is highly effective in preventing HIV acquisition only when users keep an optimal medication adherence.
  - Currently, the majority of real-world PrEP users in China buy the medication online, yet little is known about their medication adherence.
  - The cohort study aimed to evaluate the level and associated factors of PrEP adherence among real-world e-consumers in China.
- What did the researchers do and find?
  - We recruited 657 e-consumers of PrEP from the largest online platform for PrEP services in China and collected their baseline, 1-, 3-, and 6-months follow-up survey data.
  - We found PrEP adherence was low among event-driven (ED) PrEP users who accounted for the majority of real-world e-consumers, while the adherence was high among daily PrEP users.
  - Age, homosexual role, number of sexual partners, chemsex, self-efficacy of being adhere to PrEP, and PrEP-related stigma were factors significantly associated with PrEP adherence among the ED users.
- What do these findings mean?
  - There is an urgent need to improve medication adherence of e-consumers of ED PrEP in China.
  - Future target interventions can incorporate components of enhancing users’ understanding of medication instructions, promoting self-efficacy of being adhere, and alleviating PrEP-related stigma.
  - Selection bias, recall bias, social desirability bias, confounding bias, and limited representativeness were the main limitations of the study.

* Please confirm that your title complies with PLOS Medicine's style. Your title must be nondeclarative and not a question. It should begin with main concept if possible. "Effect of" should be used only if causality can be inferred, i.e., for an RCT. Please place the study design ("A randomized controlled trial," "A retrospective study," "A modelling study," etc.) in the subtitle (ie, after a colon).

Response: We have confirmed that our title complies with PLOS Medicine's style.

* Please confirm that your abstract complies with our requirements, including format (three sections: Background, Methods and Findings, and Conclusions) and providing all the information relevant to this study type <https://journals.plos.org/plosmedicine/s/submission-guidelines#loc-abstract>

Response: We have confirmed that our abstract complies with PLOS Medicine's requirements.

* Please ensure that the Introduction ends with a clear description of the study question or hypothesis.

Response: We have confirmed that the Introduction ends with a clear description of the study question or hypothesis.

* Please ensure that all abbreviations are defined at first use throughout the text.

Response: We have confirmed that all abbreviations are defined at first use throughout the text.

* Please confirm that all numbers presented in the abstract are present and identical to numbers presented in the main manuscript text.

Response: We have confirmed that all numbers presented in the abstract are present and identical to numbers presented in the main manuscript text.

**Please use the active voice throughout and request the assistance of a native English speaker to correct the grammar, phrasing and spelling throughout the text.

Response: We have used the active voice throughout and request the assistance of a native English speaker to correct the grammar, phrasing and spelling throughout the text.

* Please remove the 'conclusions' subheading from the discussion. Please also remove any other subheadings from the discussion.

Response: We have removed all the subheadings from the discussion.

"* Statistical reporting: Please revise throughout the manuscript, including tables and figures.
- Please report statistical information as follows to improve clarity for the reader ""22% (95% CI [13,28]; p</=)"".
- Please separate upper and lower bounds with commas instead of hyphens as the latter can be confused with reporting of negative values.
- Please repeat statistical definitions (HR, CI etc.) for each set of parentheses."

Response: We have revised statistical reporting format throughout the manuscript, including tables and figures.

* In the last sentence of the Abstract Methods and Findings section, please describe the main limitation(s) of the study's methodology.

Response: We have described the main limitation(s) of the study's methodology in the last sentence of the Abstract Methods and Findings section

* In the abstract, please include the important dependent variables that are adjusted for in the analyses.

Response: We have included the important dependent variables that are adjusted for in the analyses.

* We ask every co-author listed on the manuscript to fill in a contributing author statement, making sure to declare all competing interests. If any of the co-authors have not filled in the statement, we will remind them to do so when the paper is revised. If all statements are not completed in a timely fashion this could hold up the re-review process. If new competing interests are declared later in the revision process, this may also hold up the submission. Should there be a problem getting one of your co-authors to fill in a statement we will be in contact. Please do not add or remove authors without first discussing this with the handling editor. You can see our competing interests policy here: <http://journals.plos.org/plosmedicine/s/competing-interests>.

Response: We are currently following up with the co-authors to ensure that all contributing author statements and competing interest declarations are completed as soon as possible.

* For authors with ties to industry, please indicate whether any of the interests has a financial stake in the results of the current study.

Response: We confirm that there were no interests that have a financial stake in the results of the current study for the author with ties to industry.

* It appears that one or more study authors is affiliated with one or more of the agencies that funded the study. Thus, the statement “The funders had no role in study design, data collection and analysis, decision to publish, or preparation of the manuscript” does not apply. Please revise the Financial Disclosure accordingly, as in "[Author name] is [author's role] at [funding agency]. The funders had no other role in study design…..”

Response: We have further reviewed the affiliation details of all authors and confirm that no author is affiliated with agencies that funded the study. The study was founded by the National Natural Science Foundation of China (grant number: 82404326) and the Capital’s Funds for Health Improvement and Research (grant number: 2024-2G-4402). No authors are affiliated with the two funders.

Only one of the authors, Dr. Hui Xue is employed by BluedHealth, the e-commerce platform for PrEP sales in China. BluedHealth did not fund the study but just assisted in the participant recruitment in the study. It had no role in study design, data analysis and interpretation, manuscript writing, or the decision to submit the manuscript for publication. The other authors declare that they have no competing interests.

* The funding statement should include: specific grant numbers, initials of authors who received each award, URLs to sponsors’ websites. Also, please state whether any sponsors or funders (other than the named authors) played any role in study design, data collection and analysis, the decision to publish, or preparation of the manuscript. If they had no role in the research, include this sentence: “The funders had no role in study design, data collection and analysis, decision to publish, or preparation of the manuscript.”

Response: We have revised the Funding Statement to include specific grant numbers, the initials of authors who received each award, URLs to the funders’ websites, and a clear statement regarding the role of the funders in the study.

* Please upload any figures associated with your paper as individual TIF or EPS files with 300dpi resolution at resubmission; please read our figure guidelines for more information on our requirements: http://journals.plos.org/plosmedicine/s/figures. While revising your submission, we strongly recommend that you use PLOS's NAAS tool (https://ngplosjournals.pagemajik.ai/artanalysis) to test your figure files. NAAS can convert your figure files to the TIFF file type and meet basic requirements (such as print size, resolution), or provide you with a report on issues that do not meet our requirements and that NAAS cannot fix.

Response: We have uploaded all figures as individual TIFF or EPS files with 300 dpi resolution at resubmission and will check the files using the PLOS NAAS tool to ensure compliance with the figure guidelines.

* Please ensure that the paper adheres to the PLOS Data Availability Policy (see http://journals.plos.org/plosmedicine/s/data-availability), which requires that all data underlying the study's findings be provided in a repository or as Supporting Information. For data residing with a third party, authors are required to provide instructions with contact information (web or email address) for obtaining the data. Please note that a study author cannot be the contact person for the data. PLOS journals do not allow statements supported by "data not shown" or "unpublished results." For such statements, authors must provide supporting data or cite public sources that include it.

Response: Thanks. We confirm that all data underlying the findings described are fully available, without restriction, and all relevant data are within the manuscript and its Supporting Information files.

* If the data are not freely available, please describe briefly the ethical, legal, or contractual restriction that prevents you from sharing it. Please also include an appropriate contact (web or email address) for inquiries (this cannot be a study author).

Response: Thanks. We confirm that all data underlying the findings described are fully available, without restriction, and all relevant data are within the manuscript and its Supporting Information files.

* We expect all researchers with submissions to PLOS in which author-generated code underpins the findings in the manuscript to make all author-generated code available without restrictions upon publication of the work. In cases where code is central to the manuscript, we may require the code to be made available as a condition of publication. Authors are responsible for ensuring that the code is reusable and well documented. Please make any custom code available, either as part of your data deposition or as a supplementary file. Please add a sentence to your data availability statement regarding any code used in the study, e.g. "The code used in the analysis is available from Github [URL] and archived in Zenodo [DOI link]" Please review our guidelines at https://journals.plos.org/plosmedicine/s/materials-software-and-code-sharing and ensure that your code is shared in a way that follows best practice and facilitates reproducibility and reuse. Because Github depositions can be readily changed or deleted, we encourage you to make a permanent DOI'd copy (e.g. in Zenodo) and provide the URL.

Response: Thanks. We confirm that all the statistical analyses were performed by standard procedure in the R software which had been fully described in the manuscript and there were no author-generated code in the study.

* Please ensure that the study is reported according to the CROSS guideline and include the completed CROSS checklist as Supporting Information. When completing the checklist, please use section and paragraph numbers, rather than page numbers. Please add the following statement, or similar, to the Methods: "This study is reported as per CROSS guideline (S1 Checklist).
Response: We have revised the manuscript to report the study in accordance with the CROSS guideline, added the required statement to the Methods, and included the completed CROSS checklist as Supporting Information.

*Please report your survey response rates according to AAPOR recommendations <https://aapor.org/standards-and-ethics/best-practices/> Please define how the population surveyed was sampled. Please compare characteristics of respondents and nonrespondents if possible. Were sequential waves of the survey sent? If yes, did the characteristics of respondents change over time or remain constant? Please include the survey response rate in your Abstract.

Response: We have revised the manuscript to report the survey response rate according to AAPOR recommendations, clarified the sampling approach, added information on sequential survey waves, compared respondents and nonrespondents where possible, and included the response rate in the Abstract.
