# Supplementary material for: Medication adherence and its associated factors among oral pre-exposure prophylaxis (PrEP) users in China: The Real-world E-consumer Cohort of PrEP study
Source: PLoS Med. 2026 Feb 26;23(2):e1004733. doi: 10.1371/journal.pmed.1004733 (PMC12944781; doi:10.1371/journal.pmed.1004733)
Supplement: Attachment — Submitted filename: response letter-final-clean.docx [file pmed.1004733.s018.docx]

Dear Editor and Reviewers,

Thank you very much for your review and acceptance of the manuscript PMEDICINE-D-25-02683R3. We have further addressed the remaining issues.

*********************************************************************

**Editor**

E1.1 Title: Please revise to “Medication adherence and its associated factors among oral pre-exposure prophylaxis (PrEP) users in China: The Real-world E-consumer Cohort of PrEP (RECOPE) study”

Response: Thanks. We revised the title as suggested.

E1.2 Line 23: Revise “The cohort study” to “This cohort study”

Response: Thanks. We revised the wording as suggested.

**Page 2, line 22-23** *This cohort study aimed to evaluate PrEP adherence and its associated factors among e-consumers in China.*

E1.3 Line 32: please rephrase or complete the sentence. “were fitted” to what?

Response: Thanks. We clarified the purpose of the statistical analyses by specifying what the models were fitted to examine.

**Page 2, line31-32** *Generalized linear mixed-effects models and logistic regression were fitted to identify prognostic factors of PrEP adherence.*

E1.4 Line 33: Please remove paragraph break.

Response: Thanks. We removed the paragraph break as suggested.

E1.5 Line 29: were participants asked to complete e-questionnaires at 1, 3 and 6 months? If yes, please so state here.

Response: Thanks. We have added the follow-up timepoints as suggested.

**Page** **27, line** **27-28** *Anonymous self-administered e-questionnaires were used at baseline and 1-, 3-, and 6-month follow-ups to collect data on PrEP adherence and potentially associated factors.*

E1.6 Line 35: what ‘visit’ does this refer to? Please revise. Do you mean timepoints?

Response: Thanks. We replaced “visit” with “timepoints” as suggested.

**Page** **2, line** **33-35** *The follow-up response rate at the 1-, 3-, and 6-month timepoints was 90.1% (592/657), 82.6% (543/657), and 80.1% (526/657), respectively.*

E1.7 Line 48: use ‘account’ instead of ‘accounted’.

Response: Thanks. We revised the wording as suggested.

**Page 3, line 48-49** *ED PrEP users who account for the majority of real-world e-consumers in China.*

E1.8 Line 49: use ‘targeted’ instead of ‘target’

Response: Thanks. We revised the wording as suggested.

**Page 3, line 49-51** *Targeted interventions are suggested to prioritize enhancing users’ understanding of medication instructions, promoting self-efficacy of maintaining adherence, and alleviating PrEP-related stigma.*

E1.9 Line 51: replace ‘being adhere’ with ‘being adherent’ or preferably by ‘maintaining adherence’

Response: Thanks. We revised the wording as suggested.

**Page 3, line 50-51** *…promoting self-efficacy of maintaining adherence*

E1.10 Please move the Author Summary to after the Abstract.

Response: Thanks. We moved the Author Summary to after the Abstract.

E1.11 Please correct the grammar in Figure 1 (first box upper right). Please keep verb tense constant (use past tense), use ‘mo’ instead of ‘mon’ as an abbreviation for month, please clarify/revise use of ‘break-off’.

Response: Thanks. We corrected the grammar in Figure 1, unified verb tense to past tense, replaced “mon” with “month,” and revised the term “break-off” to improve clarity.

E1.12 Please correct grammar in Fig. 2c panel title “continuous adherence during among ED users”

Response: Thanks. We have changed the title of Figure 2c to “Continuous adherence among ED users”.

E1.13 Please do not use ‘visit’ for x axis titles in Figure 2 or elsewhere if what you mean is timepoint.

Response: Thanks. We replaced “visit” with “timepoint” in Figure 2

E1.14 Line 66: replace ‘toll of HIV’ with ‘tool for HIV’

Response: Thanks. We revised the wording as suggested.

**Page 5, line 90-91** *…a highly effective biomedical prevention tool of HIV*

E1.15 Line 77: delete “we can only locate”

Response: Thanks. We deleted the phrase as suggested.

**Page 5, line 102-103** *In China, a retrospective study showed a poor adherence rate as low as 50% in an on-site PrEP promotion trial.*

E1.16 Line 78: delete “which”

Response: Thanks. We deleted the word as suggested.

**Page 5, line 102-103** *In China, a retrospective study showed a poor adherence rate as low as 50% in an on-site PrEP promotion trial.*

E1.17 Line 83: revise “which is particularly accelerated” to “and whose uptake was accelerated”

Response: Thanks. We revised the wording as suggested.

**Page 6, line 106-107***…and* *whose uptake was accelerated by the COVID-19 pandemic.*

E1.18 Line 103: replace “needs to” with “must”

Response: Thanks. We revised the wording as suggested.

**Page 6-7, line 126-127** *If a client had questions, he/she must join another online counselling group on BluedHealth for further communication.*

E1.19 Line 108-109: suggest deleting “a poorer accessibility” and starting with ‘a more complex procedure’

Response: Thanks. We revised the sentence as suggested.

**Page 7, line 131-134** *Therefore, compared with offline counselling, online counselling has a more complex procedure and less flexibility of interacting with doctors, which may negatively affect clients’ understanding about PrEP use and ultimately reduce medication adherence.*

E1.20 Line 112-114: claim of primacy—if other studies have now been performed evaluating medication adherence among e-consumers of PrEP, please delete this sentence.

Response: Thanks. We deleted this claim of primacy as suggested.

E1.21 Line 115: ‘trails’ should be ‘trials’

Response: Thanks. We corrected the spelling error as suggested.

**Page 7, line 135-137** *Existing evidence on PrEP adherence were mostly from clinical/implementation/promotional trials or from real-world users who obtained PrEP from traditional offline channels.*

E1.22 Line 118: replace “easier access” with “greater accessibility”

Response: Thanks. We revised the wording as suggested.

**Page 7, line 138-140** *…oral PrEP is likely to remain a major option for the foreseeable future because of its significantly lower price and greater accessibility.*

E1.23 Line 129: replace ‘STORBE’ with ‘STROBE’

Response: Thanks. We revised the wording as suggested.

**Page 8, line 152-153** *This study is reported as per the STROBE guideline (S1 Checklist) and the CROSS Checklist (S2 Checklist).*

E1.24 Lines 132-145: This section reads as an advertisement for BluedHealth. Please remove any marketing material language and phrasing.

Response: Thanks. We previously included this section based on a comment of a reviewer who asked us to better explain the recruitment platform and the online PrEP purchasing process. We now have revised the text to avoid marketing-style language and phrasing.

**Page 8, line 155-158** *The participants were conveniently sampled and recruited through BluedHealth, the major online platform for PrEP services in China. BluedHealth provides HIV prevention and care services, such as offering sales of HIV preventive products (e.g., PrEP, post-exposure prophylaxis, condoms, HIV/syphilis/hepatitis B virus self-test kits) and related logistics and delivery. The average number of PrEP purchases per month on BluedHealth is over 4,000 [13]. During the study period, the price of PrEP on BluedHealth ranged from approximately 266 to 2280 CNY (36.7-314.5 USD) per 30-tablet bottle, depending on the manufacturer, and was comparable to the in-store price in China.*

Reference*:* *Shan D, Xue H, Yu F, Zan X, Liu H, Liu J, et al. Understanding the Uptake and Outcomes of Non-occupational Postexposure Prophylaxis Use Through an Online Medical Platform in China: Web-Based Cross-sectional Study. Journal of Medical Internet Research 2023;25:e42729. https://doi.org/10.2196/42729.*

E1.25 Line 312: explain 'break-off'

Response: Thanks. We replaced “break-off” with a more specific and standard expression.

**Page 16, line 328-329** *…and 657 completed the baseline survey (23 did not respond to the baseline questionnaire)*

E1.26 Line 378: should be 'non-adherent' not 'non-adhere'

Response: Thanks. We corrected the term as suggested.

**Page 23, line 397-398** *In addition, we further examined the patterns of missing doses among non-adherent ED users in the past month at each survey wave (S3 Table).*

E1.27 Line 441: should be 'non-adherent' not 'non-adhere'

Response: Thanks. We corrected the term as suggested.

**Page28, line 468-469** *which implies that some non-adherent ED users might still be able to protect themselves from HIV infection.*

E1.28 Line 452: delete 'all'

Response: Thanks. We deleted the word as suggested.

**Page 28, line 477-478** *The prevalence of optimal PrEP adherence among the daily users in the study was above 80%...*

E1.29 Line 473: please correct grammar. "suggested incorporate' should be "suggested to incorporate".

Response: Thanks. We corrected the grammar as suggested.

**Page 29, line 497-498** *Future PrEP adherence interventions are suggested to incorporate self-efficacy enhancement strategies…*

E1.30 Line 485: remove "fortunately" as may be considered demeaning. Suggest replacing with "notably"

Response: Thanks. We revised the wording as suggested.

**Page 30, line 510-511** *Notably, participants who identified their sexual role as receptive or versatile…*

E1.31 Line 499: delete "Additionally,"

Response: Thanks. We deleted the word as suggested.

**Page 30, line 524-525** *Older ED PrEP users showed a higher prevalence of consistent adherence in this study, consistent with findings from a study in the U.S.*

E1.32 Line 514-515: delete "were unavailable" due to redundancy. "But as no information were available " should be "But as no information was available"

Response: Thanks. We deleted the redundant wording and corrected the grammar as suggested.

**Page 31, line 537-538** *But as no information was available for the non-respondents, the exact direction and magnitude of selection bias could not be gauged.*

E1.33 Line 525: should be "being adherent"

Response: Thanks. We revised the wording as suggested.

**Page 31-32, line 547-549** *For example, the adherence outcome, PrEP knowledge, and self-efficacy of being adherent showed an increase to some extent from baseline to subsequent follow-ups in the study.*

E1.34 Line 526-529: please correct grammar in this sentence.

Response: Thanks. We corrected the grammar as suggested.

**Page 32, line 549-552** *Possible explanations include that repeated follow-up surveys may have reinforced adherence behaviors, encouraged participants to acquire PrEP-related knowledge, and enhanced their self-efficacy.*

E1.35 Line 533: measurement should be plural

Response: Thanks. We revised the wording as suggested.

**Page 32, line 556-558** *However, the study did not include measurements that directly evaluate the participants’ actual reception of online counselling and their perceived quality and satisfaction of the counselling, as well as their perceived influence or importance of counseling on adherence.*

E1.36 Line 558: should be "accounts" not "accounted"

Response: Thanks. We revised the wording as suggested.

**Page 33, line 579-580** *Since ED users accounts for the majority of real-world e-consumers of PrEP in China…*

E1.37 Please reduce the repetition of ideas and concepts in the Discussion.

Response: Thanks. We have carefully read through the Discussion section and reduce the repetition of ideas and concepts.

E1.38 For each table, define the abbreviations used in the footnotes.

Response: Thanks. We defined all abbreviations used in each table in the corresponding table footnotes.

E1.39 Please make formatting style consistent across the top of Tables 1 and 2.

Response: Thanks. We have ensured that the formatting style across the top of Tables 1 and 2 is consistent.

E1.40 Please avoid use of homosexual and use same sex instead (e.g. lines 39, 402, Table 2 footnotes, Table 3, Author summary. Please check the supplementary information and revise if necessary.).

Response: Thanks. We replaced the term “homosexual” with “same-sex” throughout the manuscript and supplementary information to ensure appropriate and consistent terminology.

E1.41 Please check throughout for grammatical errors.

Response: Thanks. We carefully reviewed the manuscript and corrected grammatical errors throughout the main text, tables, figures, and supplementary materials.

E1.42 Please seek the assistance of a native English speaker when revising your manuscript.

Response: Thanks. The manuscript was revised with the assistance of a native English speaker to improve language clarity and readability.

E1.43 Please do not use the term ‘visit’ for survey questionnaire timepoints in the main manuscript or the supplementary documents.

Response: Response: Thanks. We replaced the term “visit” with “timepoint” throughout the main manuscript and the supplementary documents to ensure consistent and accurate terminology.

E1.44 Please clarify whether written or oral informed consent was obtained and add to Methods.

Response: Thanks. We clarified that oral informed consent was obtained and added this information to the Methods.

**Page 9, line 180-184** *Those who were eligible and agreed to join the study were then asked to provide an oral informed consent by clicking the agreement button in an e-informed consent form sent by the researcher via WeChat to preserve anonymity. Each enrolled participant was assigned a unique ID for linking baseline and follow-up surveys.*

* If any included images of branded products are included in the manuscript files, please confirm that you have appropriate permission or you may wish to remove these images. For more information, please see our guidelines: https://journals.plos.org/plosmedicine/s/figures#loc-licenses-and-copyright and <https://journals.plos.org/plosmedicine/s/licenses-and-copyright>

Response: We confirm that no images of branded products are included in the manuscript files.

* Please review your text for claims of novelty or primacy (e.g. 'for the first time') and remove this language. In addition, please check that any use of statistical terms (such as trend or significant) are supported by the data, and if not please remove them.

Response: We confirm that we have removed any language claiming of novelty or primacy, and we also reviewed the manuscript and revised statistical terminology to ensure it is supported by the data.

When completing the CROSS checklist, please use section and paragraph numbers, rather than page numbers. Note that page numbers are currently used.

Response: We updated the CROSS checklist to use section and paragraph numbers instead of page numbers.

* Please compare characteristics of respondents and nonrespondents if possible.

Response: Due to limited available information on nonrespondents, a formal comparison could not be conducted and this has been acknowledged as a limitation.

* If sequential waves of the survey were sent, please specify whether the characteristics of respondents changed over time or remained constant.

Response: Thanks. We have examined whether respondent characteristics differed across sequential survey waves. Socio-demographic characteristics remained largely constant over time, whereas sexual behaviors, PrEP-related variables, and psychosocial variables varied across timepoints; these patterns are presented in Tables 1 and 2.

Please report your survey response rates according to AAPOR recommendations https://aapor.org/standards-and-ethics/best-practices/

Response: Thanks. We confirmed that the survey response rates were calculated and reported in accordance with AAPOR recommendations and are presented in Figure 1.

* Please provide titles and legends for all figures and tables (including those in Supporting Information files). Please define all acronyms used in each figure or table in its corresponding legend.

Response: Thanks. We provided titles and legends for all figures and tables, including those in the Supporting Information files, and defined all acronyms used in each figure or table in the corresponding legends.

*********************************************************************

**Reviewer #2**

R2.1 The changes to the manuscript that position the multivariable regression analyses as prognosis factor research resolve my query from the last review.

Response: We thank the reviewer for all the comments and suggestions.

*********************************************************************
